# Supplementary material for: Renal coenzyme A (CoA) production from VB5 fuels stem cell proliferation and tumor growth
Source: Nat Commun. 2026 Apr 18;17:5383. doi: 10.1038/s41467-026-71716-1 (PMC13276073; doi:10.1038/s41467-026-71716-1)
Supplement: Supplementary file 1 — Supplementary Information [file 41467_2026_71716_MOESM1_ESM.pdf]

Supplementary Information for

Renal Coenzyme A (CoA) Production from VB5 Fuels Stem Cell Proliferation and Tumor Growth

Ting Miao[1,^], Ying Liu[1], Mujeeb Qadiri[1], Amaury Dasseux[2], John M Asara[3], Yanhui Hu[1], Xiaomei Sun[1], Luz del Carmen Pliego-Alcántara[2], Christian C. Dibble[2,^], Norbert Perrimon[1,4,^]

[1] Department of Genetics, Blavatnik Institute, Harvard Medical School, Boston, MA, USA.

[2] Department of Pathology, Cancer Research Institute, Beth Israel Deaconess Medical Center, Harvard Medical School, Boston, MA, USA

[3] Mass Spectrometry Core, Beth Israel Deaconess Medical Center and Department of Medicine, Harvard Medical School, Boston, MA USA

[4] Howard Hughes Medical Institute, Boston, MA, USA

^Correspondence:

Ting Miao: ting_miao@hms.harvard.edu

Norbert Perrimon: perrimon@genetics.med.harvard.edu

Christian C. Dibble: ccdibble@bidmc.harvard.edu

Containing:

Figs. S1 to S6

Supplementary Table 1-4

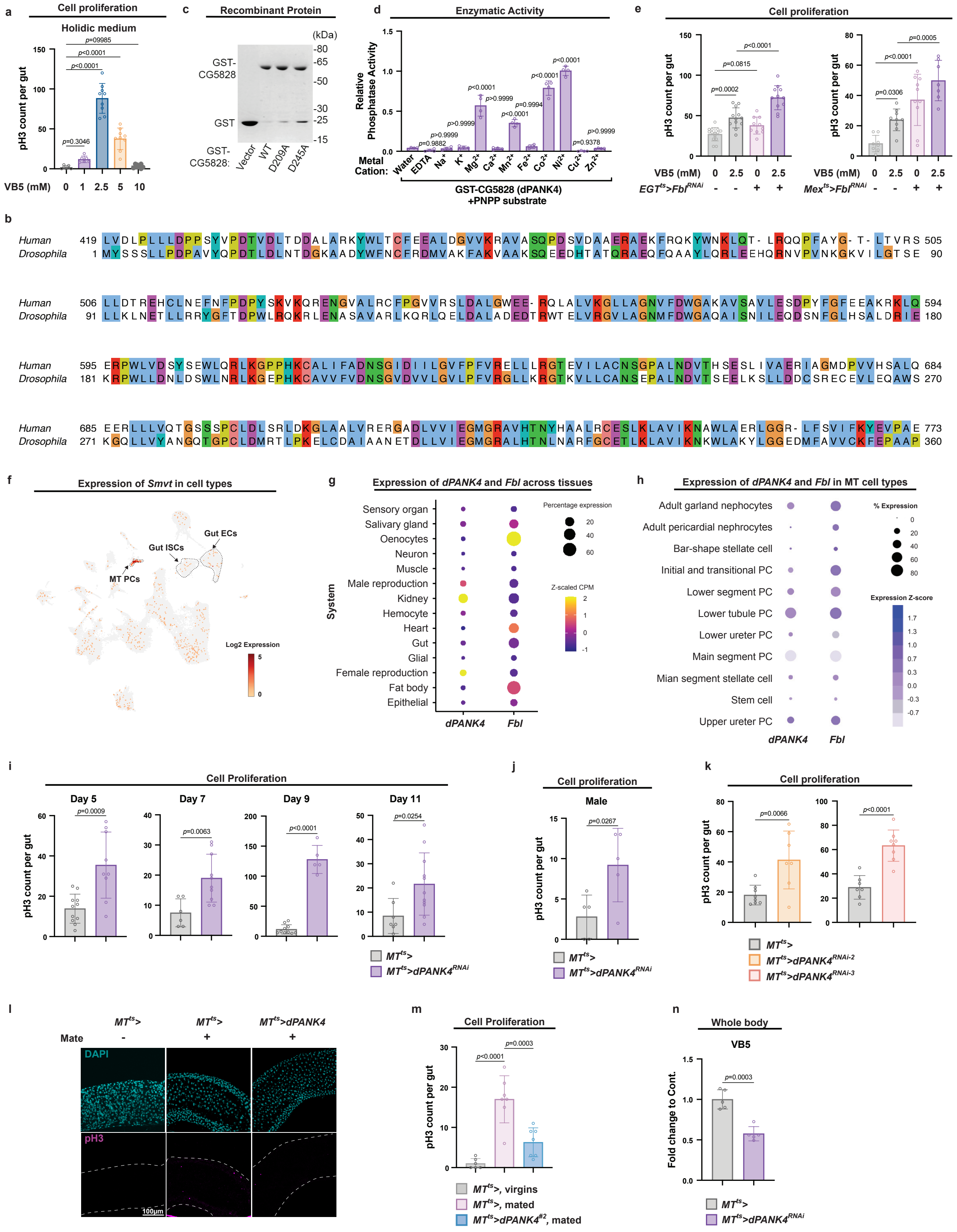
a
Cell proliferation
Holidic medium
pH3 count per gut
VB5 (mM)
c
Recombinant Protein
(kDa)
GST-CG5828
GST
GST-CG5828:
Vector
WT
D209A
D245A
d
Enzymatic Activity
Relative Phosphatase Activity
Metal Cation:
GST-CG5828 (dPANK4) +PNPP substrate
e
Cell proliferation
pH3 count per gut
VB5 (mM)
EGTts>FblRNAi
Mexts>FblRNAi
b
Human
Drosophila
f
Expression of Smvt in cell types
Gut ECs
Gut ISCs
MT PCs
Log2 Expression
g
Expression of dPANK4 and Fbl across tissues
Percentage expression
Z-scaled CPM
System
Sensory organ
Salivary gland
Oenocytes
Neuron
Muscle
Male reproduction
Kidney
Hemocyte
Heart
Gut
Glial
Female reproduction
Fat body
Epithelial
dPANK4
Fbl
h
Expression of dPANK4 and Fbl in MT cell types
% Expression
Expression Z-score
Adult garland nephrocytes
Adult pericardial nephrocytes
Bar-shape stellate cell
Initial and transitional PC
Lower segment PC
Lower tubule PC
Lower ureter PC
Main segment PC
Mian segment stellate cell
Stem cell
Upper ureter PC
i
Cell Proliferation
Day 5
Day 7
Day 9
Day 11
pH3 count per gut
MTts>
MTts>dPANK4RNAi
j
Cell proliferation
Male
k
Cell proliferation
MTts>dPANK4RNAi-2
MTts>dPANK4RNAi-3
l
Mate
MTts>dPANK4
DAPI
pH3
100μm
m
Cell Proliferation
MTts>, virgins
MTts>, mated
MTts>dPANK4#2, mated
n
Whole body
VB5
Fold change to Cont.

Fig. S1 | Related to Figure 1 and 2. a, Quantification of pH3$^+$ cells per midgut from flies with VB5 dietary supplementation at 0, 1, 2.5, 5, or 10 mM on holidic medium. n = 7, 6, 9, 12, 9, 9, and 10 in 0, 1, 2.5, 5, and 10 mM, respectively. **b,** Full length amino acid alignment between human PANK4 (NCBI Reference Sequence: NP_060686.3) and *Drosophila* CG5828 (dPANK4; NCBI Reference Sequence: NP_608907.1). **c,** Coomassie stained SDS-PAGE of vector and GST-CG5828 purification showing relative quantities used in reactions shown in **Fig. 2c**. **d,** Metal cation dependence of CG5828/dPANK4 phosphatase activity. Metal cation preference was determined as in **Fig. 2c** with PNPP substrate and chloride salts of indicated metal cations (0.5mM). For statistical analysis, the relative activities of the GST-CG5828 in chloride salts or EDTA are compared with that in water. n = 4. **e,** Quantification of pH3$^+$ cells per midgut in control versus ISC/EB-specific (*EGT^{ts}*>; left) or EC-specific (*Mex^{ts}*>; right) *Fbl* knockdown flies under VB5 supplementation. n = 14, 12, 10, and 11 in *EGT^{ts}*> (0 mM), *EGT^{ts}*> (2.5 mM), *EGT^{ts}> Fbl-RNAi* (0 mM), and *EGT^{ts}> Fbl-RNAi* (2.5 mM), respectively. n = 8, 9, 10, and 7 in *Mex^{ts}*> (0 mM), *Mex^{ts}*> (2.5 mM), *Mex^{ts}> Fbl-RNAi* (0 mM), and *Mex^{ts}> Fbl-RNAi* (2.5 mM), respectively. **f.** UMAP visualization of expression levels of *Smvt* across all cell clusters; data retrieved from published snRNA-seq. Malpighian tubule (MT) principal cells (PCs), gut intestinal stem cells (ISCs), and gut enterocytes (ECs) are outlined with dashed lines. **g,** Expression patterns of *dPANK4* and *Fbl* across tissues based on percentage of expressing cells and Z-scaled counts per million (CPM); data from FlyAtlas single-nucleus RNA-seq (snRNA-seq) database. **h,** Expression of *dPANK4* and *Fbl* in cell types of the MTs, represented by percent-expressing cells and Z-scored expression; data from published MT snRNA-seq dataset. PC, principal cell. **i,** Quantification of pH3$^+$ cells per midgut at day 5, 7, 9, and 11 in control and MT-specific *dPANK4* knockdown flies. n = 11 and 9 in *MT^{ts}*> and *MT^{ts}>dPANK4-RNAi* at Day 5, respectively. n = 6 and 11 in *MT^{ts}*> and *MT^{ts}>dPANK4-RNAi* at Day 7, respectively. n = 10 and 5 in *MT^{ts}*> and *MT^{ts}>dPANK4-RNAi* at Day 9, respectively. n = 7 and 11 in *MT^{ts}*> and *MT^{ts}>dPANK4-RNAi* at Day 11, respectively. **j,** pH3$^+$ cell counts in midguts from male flies with or without MT-specific *dPANK4* knockdown at day 10. n = 5. **k,** pH3$^+$ cell counts in midguts of flies with or without MT-specific dPANK4 *knockdown* at day 10 using two additional independent RNAi lines. n = 9 and 7 in *MT^{ts}*> and *MT^{ts}>dPANK4-RNAi-#2*, respectively. n = 7 and 8 in *MT^{ts}*> and *MT^{ts}>dPANK4-RNAi-#3*, respectively. **l,** Representative gut images from virgin and mated female flies with or without MT-specific *dPANK4* overexpression at day 7. In the pH3 panel, the guts are outlined with dashed lines. **m,** Quantification of pH3$^+$ cells per midgut from virgin and mated female flies with or without MT-specific *dPANK4* overexpression at day 7 using an independent *dPANK4* overexpression line. n = 6, 7 and 7 in *MT^{ts}*> (virgins), *MT^{ts}*> (mated), and *MT^{ts}>dPANK4-#2 MT^{ts}*> (mated), respectively. **n,** Relative metabolite levels of VB5 in control and MT-specific *dPANK4* knockdown flies at day 9, retrieved from whole-body metabolomics analysis. n = 5. Statistical significance assessed by one-way ANOVA (**a, d, e, m)** and unpaired two-sided Student's *t*-test (**i, j, k, n**) and. Error bars indicate s.d., with means at the center. Source data are provided as a Source Data file.

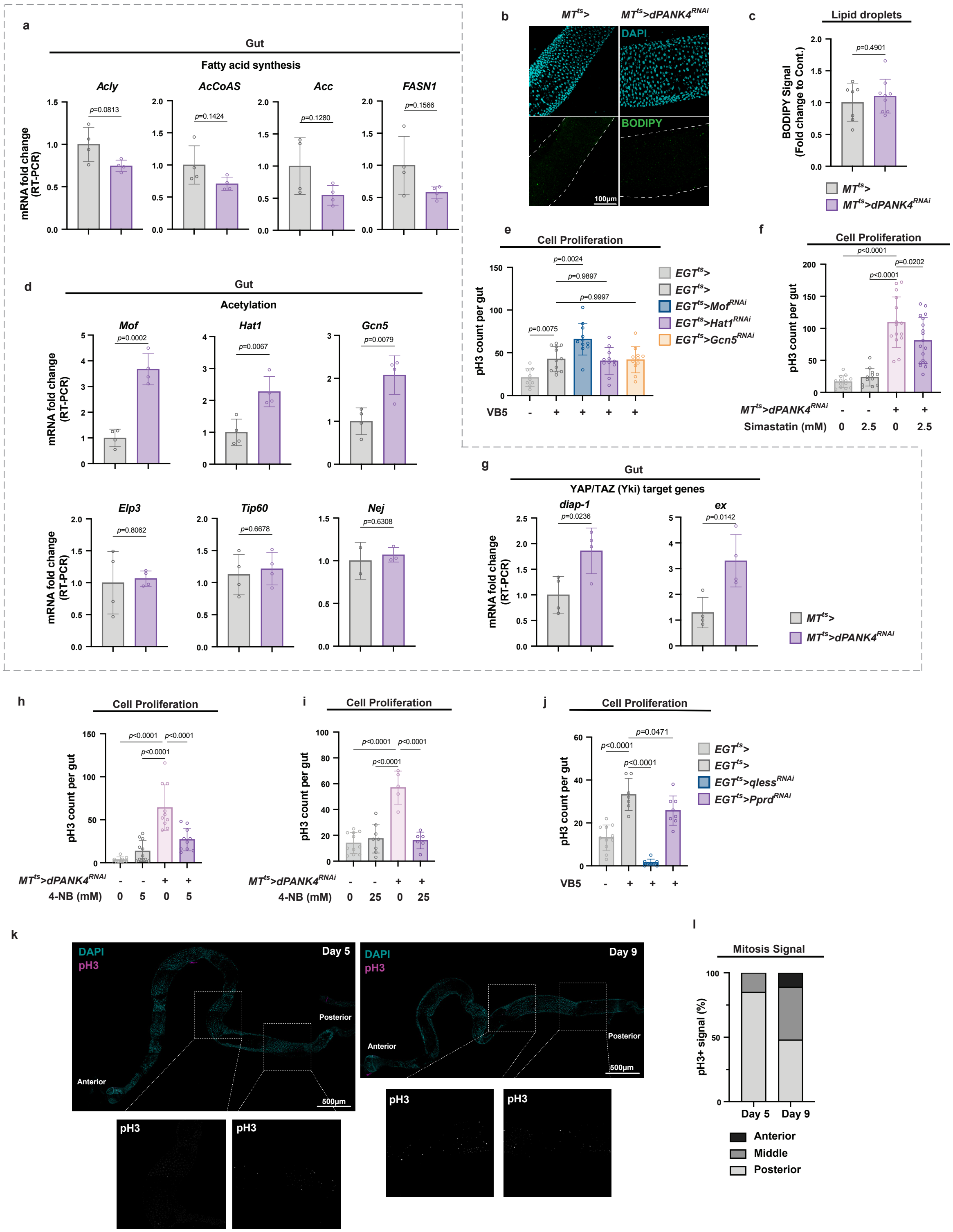
a
Gut
Fatty acid synthesis
Acly
AcCoAS
Acc
FASN1
p=0.0813
p=0.1424
p=0.1280
p=0.1566
mRNA fold change (RT-PCR)
b
MTts>
MTts>dPANK4RNAi
DAPI
BODIPY
100μm
c
Lipid droplets
p=0.4901
BODIPY Signal (Fold change to Cont.)
MTts>
MTts>dPANK4RNAi
d
Gut
Acetylation
Mof
Hat1
Gcn5
p=0.0002
p=0.0067
p=0.0079
Elp3
Tip60
Nej
p=0.8062
p=0.6678
p=0.6308
mRNA fold change (RT-PCR)
e
Cell Proliferation
p=0.0024
p=0.9897
p=0.9997
p=0.0075
pH3 count per gut
VB5
EGTts>
EGTts>
EGTts>MofRNAi
EGTts>Hat1RNAi
EGTts>Gcn5RNAi
f
Cell Proliferation
p<0.0001
p=0.0202
p<0.0001
pH3 count per gut
MTts>dPANK4RNAi
Simastatin (mM) 0 2.5 0 2.5
g
Gut
YAP/TAZ (Yki) target genes
diap-1
ex
p=0.0236
p=0.0142
mRNA fold change (RT-PCR)
MTts>
MTts>dPANK4RNAi
h
Cell Proliferation
p<0.0001
p<0.0001
p<0.0001
pH3 count per gut
MTts>dPANK4RNAi
4-NB (mM) 0 5 0 5
i
Cell Proliferation
p<0.0001
p<0.0001
p<0.0001
pH3 count per gut
MTts>dPANK4RNAi
4-NB (mM) 0 25 0 25
j
Cell Proliferation
p=0.0471
p<0.0001
p<0.0001
pH3 count per gut
VB5
EGTts>
EGTts>
EGTts>qlessRNAi
EGTts>PprdRNAi
k
DAPI
pH3
Day 5
Day 9
Anterior
Posterior
500μm
pH3
l
Mitosis Signal
pH3+ signal (%)
Day 5
Day 9
Anterior
Middle
Posterior

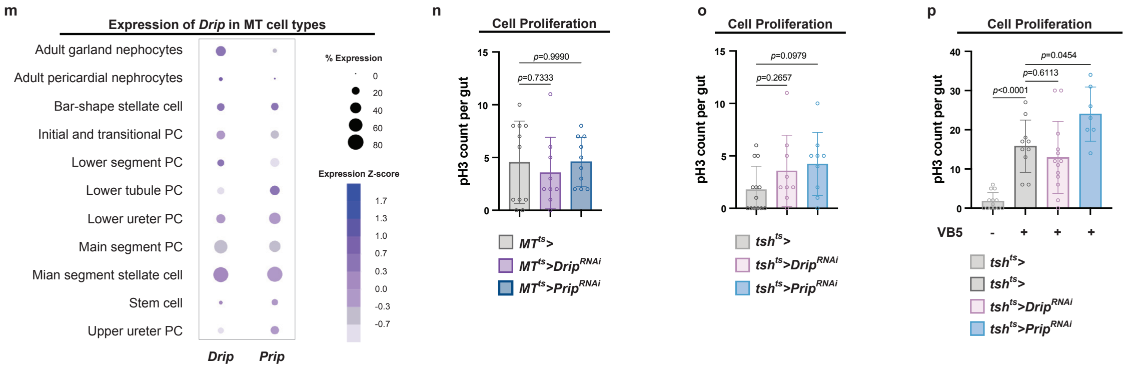

m
Expression of Drip in MT cell types
Adult garland nephocytes
Adult pericardial nephrocytes
Bar-shape stellate cell
Initial and transitional PC
Lower segment PC
Lower tubule PC
Lower ureter PC
Main segment PC
Mian segment stellate cell
Stem cell
Upper ureter PC
Drip
Prip
% Expression
0
20
40
60
80
Expression Z-score
1.7
1.3
1.0
0.7
0.3
0.0
-0.3
-0.7
n
Cell Proliferation
pH3 count per gut
p=0.9990
p=0.7333
MTts>
MTts>DripRNAi
MTts>PripRNAi
o
Cell Proliferation
pH3 count per gut
p=0.0979
p=0.2657
tshts>
tshts>DripRNAi
tshts>PripRNAi
p
Cell Proliferation
pH3 count per gut
p=0.0454
p=0.6113
p<0.0001
VB5
-
+
+
+
tshts>
tshts>
tshts>DripRNAi
tshts>PripRNAi

Fig. S2 | Related to Figure 3. a, qRT-PCR analysis of lipogenic genes (*Acly, AcCoAS, Acc,* and *FASN1*) mRNA levels in guts from control and MT-specific *dPANK4* knockdown flies at day 9. n = 4. Acly, ATP citrate lyase, ortholog of ATPCL; AcCoAS, acetyl-CoA synthetase, ortholog of ACSS1/2; ACC, acetyl-CoA carboxylase; FASN1, fatty acid synthase 1. **b, c,** Representative images (**b**) and quantification (**c**) of BODIPY lipid staining in guts from control and MT *dPANK4* knockdown flies at day 9. In the BODIPY panel, the guts are outlined with dashed lines. n = 7 and 9 in *MT^{ts}>* and *MT^{ts}>dPANK4-RNAi*, respectively. **d,** qRT-PCR analysis of histone acetyltransferase genes (*Mof, HAT1, Gcn5, Elp3, Tip60,* and *Nej*) mRNA levels in guts from control and MT *dPANK4* knockdown flies at day 9. n = 4. Nej, ortholog of CBP/p300. **e,** Quantification of $pH3^+$ cells per midgut in flies with gut-specific knockdown of *HAT1, Mof,* or *Gcn5* under VB5 supplementation. n = 9, 12, 11, 12, and 11 in *EGT^{ts}>* [VB5 (-)], *EGT^{ts}>* [VB5 (+)], *EGT^{ts}> Mof-RNAi* [VB5 (+)], *EGT^{ts}> Hat1-RNAi* [VB5 (+)], and *EGT^{ts}> Gcn5-RNAi* [VB5 (+)], respectively. **f,** Quantification of $pH3^+$ cells per midgut in flies with or without MT-specific *dPANK4* knockdown at day 10. Flies were fed on DMSO or simvastatin-containing (2.5 mM) diet for 5 days. n = 13, 12, 15, and 18 in *MT^{ts}>* (0 mM), *MT^{ts}>* (2.5 mM), *MT^{ts}>dPANK4-RNAi* (0 mM), and *MT^{ts}>dPANK4-RNAi* (2.5mM), respectively. **g,** qRT-PCR analysis of YAP/TAZ (Yki) target genes (*diap-1* and *expanded*) mRNA levels in guts from control and MT-specific *dPANK4* knockdown flies at day 9. n = 4. **h-i,** Quantification of $pH3^+$ cells per midgut in flies with or without MT *dPANK4* depletion, fed on diet supplemented with 5 mM (**h**) or 25 mM (**i**) 4-nitrobenzoic acid (4-NB). n = 10, 11, 10, and 10 in *MT^{ts}>* (0 mM), *MT^{ts}>* (5 mM), *MT^{ts}>dPANK4-RNAi* (0 mM), and *MT^{ts}>dPANK4-RNAi* (5mM) in **h**, respectively. n = 11, 8, 5, and 6 in *MT^{ts}>* (0 mM), *MT^{ts}>* (25 mM), *MT^{ts}>dPANK4-RNAi* (0 mM), and *MT^{ts}>dPANK4-RNAi* (25mM) in **i**, respectively. **j,** $pH3^+$ cell quantification in control flies or flies with gut-specific knockdown of *qless* or *Pprd* under VB5 supplementation. n = 11, 7, 8, and 8 in *EGT^{ts}>* [VB5 (-)], *EGT^{ts}>* [VB5 (+)], *EGT^{ts}> qless-RNAi* [VB5 (+)], and *EGT^{ts}> Pprd-RNAi* [VB5 (+)], respectively. **k,** Representative whole midgut images from control and MT *dPANK4* knockdown flies at day 5 and day 9 showing posterior-biased $pH3^+$ signal at day 5 and anterior progression by day 9. **l,** Distribution of mitosis signal in anterior (R1-2), middle (R3), and posterior (R4-5) guts of control and MT *dPANK4* knockdown flies at day 5 and day 9. n = 3. **m,** Expression profile of *Drip* and *Prip* in MT cell types, shown as percent of expressing cells and Z-scored expression; data from published MT snRNA-seq dataset. **n,** Quantification of $pH3^+$ cells per midgut in control flies and flies with *Drip* or *Prip* knockdown in principal cells (*MT^{ts}>*). n = 13, 9, and 10 in *MT^{ts}>*, *MT^{ts}>Drip-RNAi*, and *MT^{ts}>Prip-RNAi*, respectively. **o,** Quantification of $pH3^+$ cells per midgut in control flies and flies with *Drip* or *Prip* knockdown in stellate cells (*tsh^{ts}>*). n = 13, 9, and 11 in *tsh^{ts}>*, *tsh^{ts}>Drip-RNAi*, and *tsh^{ts}>Prip-RNAi*, respectively. **p,** Quantification of $pH3^+$ cells per midgut in control flies and flies with *Drip* or *Prip* knockdown in stellate cells (*tsh^{ts}>*) under VB5 supplementation. n = 13, 10, 14 and 7 in *tsh^{ts}>* [VB5 (-)], *tsh^{ts}>* [VB5 (+)], *tsh^{ts}>Drip-RNAi* [VB5 (+)], and *tsh^{ts}>Prip-RNAi* [VB5 (+)], respectively. Statistical significance assessed by unpaired two-sided Student's *t*-test (**a, c, d, g**) and one-way ANOVA (**e, f, h, l, j, n, o, p)**. Error bars indicate s.d., with means at the center. Source data are provided as a Source Data file.

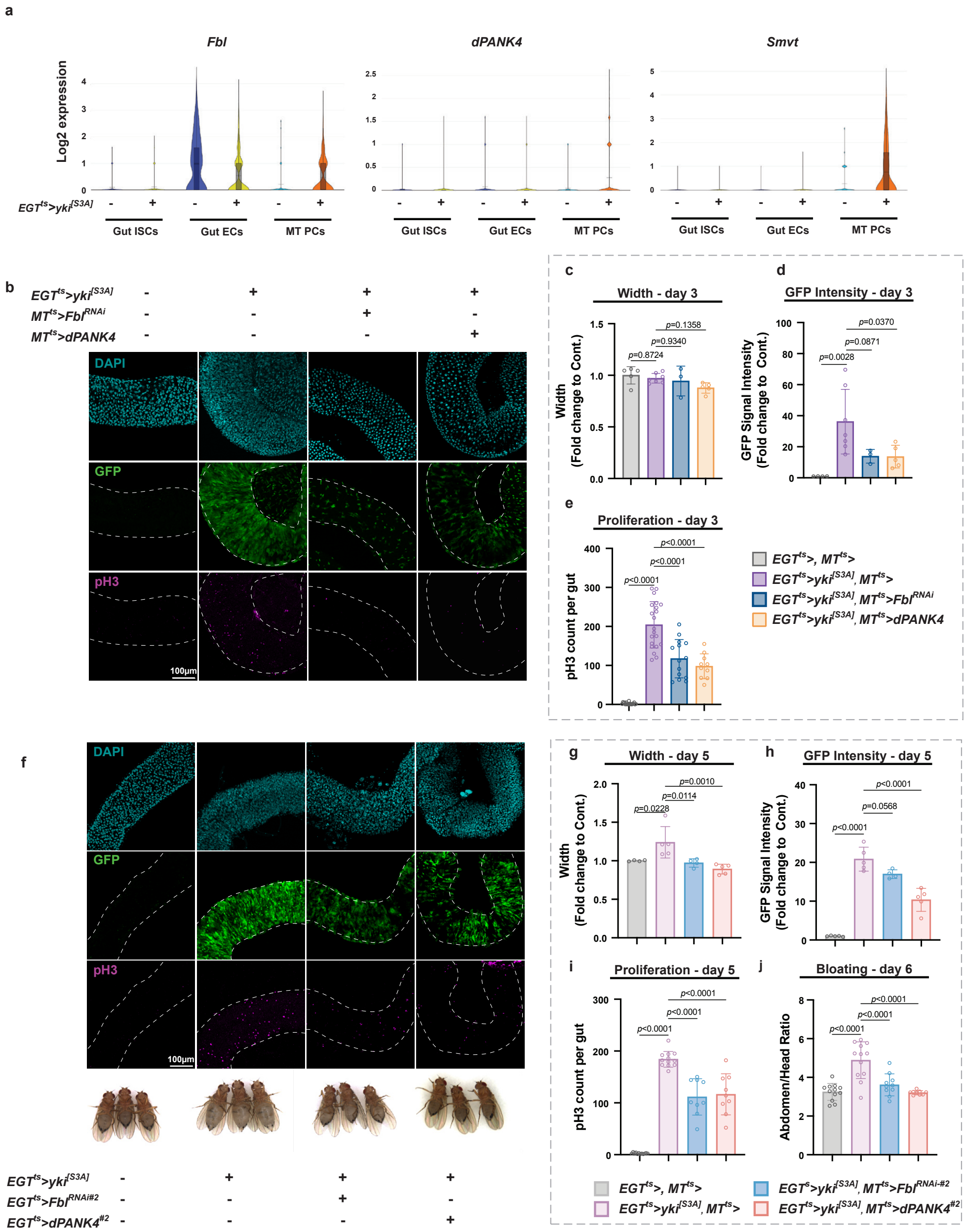

a
Fbl
dPANK4
Smvt
Log2 expression
EGTts>yki[S3A]
Gut ISCs
Gut ECs
MT PCs
b
EGTts>yki[S3A]
MTts>FblRNAi
MTts>dPANK4
DAPI
GFP
pH3
100μm
c
Width - day 3
Width (Fold change to Cont.)
p=0.8724
p=0.9340
p=0.1358
d
GFP Intensity - day 3
GFP Signal Intensity (Fold change to Cont.)
p=0.0028
p=0.0871
p=0.0370
e
Proliferation - day 3
pH3 count per gut
p<0.0001
p<0.0001
p<0.0001
EGTts>, MTts>
EGTts>yki[S3A], MTts>
EGTts>yki[S3A], MTts>FblRNAi
EGTts>yki[S3A], MTts>dPANK4
f
DAPI
GFP
pH3
100μm
EGTts>yki[S3A]
EGTts>FblRNAi#2
EGTts>dPANK4#2
g
Width - day 5
Width (Fold change to Cont.)
p=0.0228
p=0.0114
p=0.0010
h
GFP Intensity - day 5
GFP Signal Intensity (Fold change to Cont.)
p<0.0001
p=0.0568
p<0.0001
i
Proliferation - day 5
pH3 count per gut
p<0.0001
p<0.0001
p<0.0001
j
Bloating - day 6
Abdomen/Head Ratio
p<0.0001
p<0.0001
p<0.0001
EGTts>, MTts>
EGTts>yki[S3A], MTts>
EGTts>yki[S3A], MTts>FblRNAi-#2
EGTts>yki[S3A], MTts>dPANK4#2

k

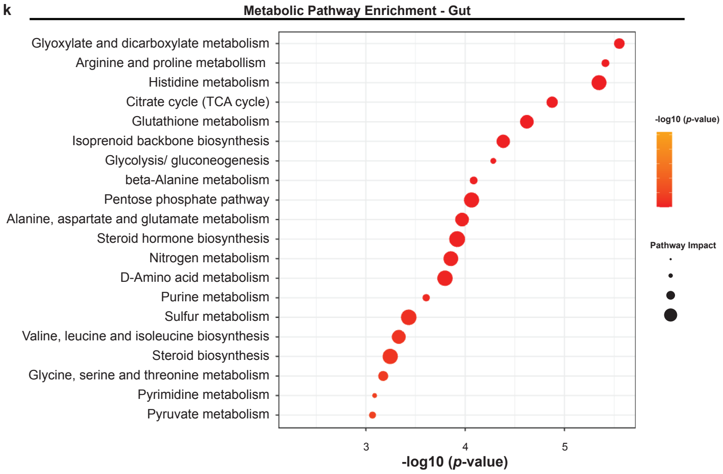

Metabolic Pathway Enrichment - Gut
Glyoxylate and dicarboxylate metabolism
Arginine and proline metabollism
Histidine metabolism
Citrate cycle (TCA cycle)
Glutathione metabolism
Isoprenoid backbone biosynthesis
Glycolysis/ gluconeogenesis
beta-Alanine metabolism
Pentose phosphate pathway
Alanine, aspartate and glutamate metabolism
Steroid hormone biosynthesis
Nitrogen metabolism
D-Amino acid metabolism
Purine metabolism
Sulfur metabolism
Valine, leucine and isoleucine biosynthesis
Steroid biosynthesis
Glycine, serine and threonine metabolism
Pyrimidine metabolism
Pyruvate metabolism
3
4
5
-log10 (p-value)
-log10 (p-value)
Pathway Impact

l

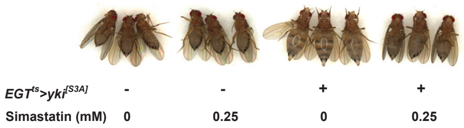

$EGT^{ts}>yki^{[S3A]}$ - - + +
Simastatin (mM) 0 0.25 0 0.25

m

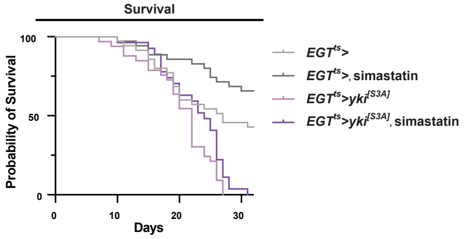

Survival
Probability of Survival
100
50
0
0
10
20
30
Days
$EGT^{ts}>$
$EGT^{ts}>$, simastatin
$EGT^{ts}>yki^{[S3A]}$
$EGT^{ts}>yki^{[S3A]}$, simastatin

Fig. S3 | Related to Figure 4. a, Log2 expression of *dPANK4*, *Fbl*, and *Smvt* in gut ISCs, gut ECs and MT PCs of control and Yki flies; data from published whole-body Yki snRNA-seq dataset. **b,** Representative images of guts from control flies and Yki flies with or without MT-specific *Fbl* knockdown or *dPANK4* overexpression at day 3. In the GFP and pH3 panels, the guts are outlined with dashed lines. **c–e,** Quantification of gut width (**c**), GFP intensity (**d**), and $pH3^+$ cell counts (**e**). n = 5, 7, 3, and 5 in EGT^{ts}>, MT^{ts}>, EGT^{ts}>$yki^{[S3A]}$, MT^{ts}>, EGT^{ts}>$yki^{[S3A]}$, MT^{ts}>*Fbl-RNAi*, and EGT^{ts}>$yki^{[S3A]}$, MT^{ts}>*dPANK4* in **c**, respectively. n = 5, 7, 3, and 5 in EGT^{ts}>, MT^{ts}>, EGT^{ts}>$yki^{[S3A]}$, MT^{ts}>, EGT^{ts}>$yki^{[S3A]}$, MT^{ts}>*Fbl-RNAi*, and EGT^{ts}>$yki^{[S3A]}$, MT^{ts}>*dPANK4* in **d**, respectively. n = 24, 21, 14, and 10 in EGT^{ts}>, MT^{ts}>, EGT^{ts}>$yki^{[S3A]}$, MT^{ts}>, EGT^{ts}>$yki^{[S3A]}$, MT^{ts}>*Fbl-RNAi*, and EGT^{ts}>$yki^{[S3A]}$, MT^{ts}>*dPANK4* in **e**, respectively. **f,** Representative images of guts (day 5) and representative images of bloating phenotypes (day 6) from control flies and Yki flies with or without MT-specific *Fbl* knockdown or *dPANK4* overexpression using independent fly lines. **g-i,** Quantification of gut width (**g**), GFP intensity (**h**), and $pH3^+$ cell counts (**i**). n = 4, 5, 4, and 5 in EGT^{ts}>, MT^{ts}>, EGT^{ts}>$yki^{[S3A]}$, MT^{ts}>, EGT^{ts}>$yki^{[S3A]}$, MT^{ts}>*Fbl-RNAi-#2*, and EGT^{ts}>$yki^{[S3A]}$, MT^{ts}>*dPANK4-#2* in **g**, respectively. n = 5, 5, 4, and 5 in EGT^{ts}>, MT^{ts}>, EGT^{ts}>$yki^{[S3A]}$, MT^{ts}>, EGT^{ts}>$yki^{[S3A]}$, MT^{ts}>*Fbl-RNAi-#2*, and EGT^{ts}>$yki^{[S3A]}$, MT^{ts}>*dPANK4-#2* in **h**, respectively. n = 15, 14, 9, and 9 in EGT^{ts}>, MT^{ts}>, EGT^{ts}>$yki^{[S3A]}$, MT^{ts}>, EGT^{ts}>$yki^{[S3A]}$, MT^{ts}>*Fbl-RNAi-#2*, and EGT^{ts}>$yki^{[S3A]}$, MT^{ts}>*dPANK4-#2* in **i**, respectively. **j,** Bloating quantification via abdomen-to-head ratio of panel **f**. n = 12, 12, 10, and 12 in EGT^{ts}>, MT^{ts}>, EGT^{ts}>$yki^{[S3A]}$, MT^{ts}>, EGT^{ts}>$yki^{[S3A]}$, MT^{ts}>*Fbl-RNAi-#2*, and EGT^{ts}>$yki^{[S3A]}$, MT^{ts}>*dPANK4-#2*, respectively. **k,** Pathway enrichment analysis of gut metabolomics profiling of flies with Yki flies versus controls at day 6. The top enriched KEGG pathways are shown, ranked by statistical significance (-log10(*p*-value)) and colored accordingly. Dot size represents pathway value. n = 4. **l,** Representative images of bloating phenotypes in control and Yki flies with or without simvastatin treatment (0.25 mM) at day 6. Flies were fed on DMSO or simvastatin-containing diet for 6 days. **m**, Survival curve of control and Yki flies with or without simvastatin treatment (0.25 mM). n = 27-35. Statistical significance assessed by one-way ANOVA (**c-e, g-j**), two-tailed Fisher's exact test (**k**), and log-rank (Mantel–Cox) test (**m**). Error bars indicate s.d., with means at the center. Source data are provided as a Source Data file.

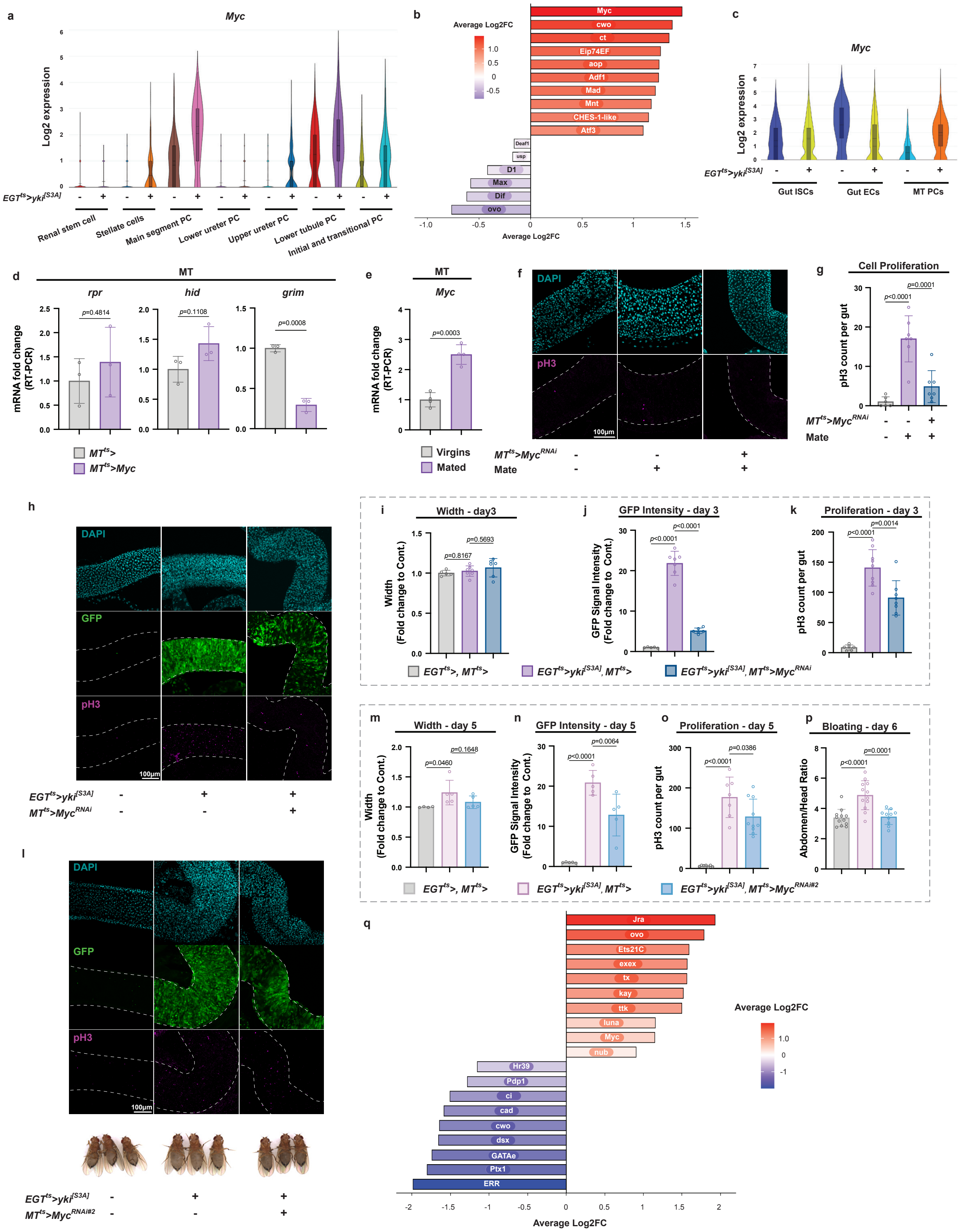

a
Myc
Log2 expression
EGT^ts>yki^[S3A]
Renal stem cell
Stellate cells
Main segment PC
Lower ureter PC
Upper ureter PC
Lower tubule PC
Initial and transitional PC
b
Average Log2FC
Myc
cwo
ct
Eip74EF
aop
Adf1
Mad
Mnt
CHES-1-like
Atf3
Deaf1
usp
D1
Max
Dif
ovo
c
Myc
Log2 expression
Gut ISCs
Gut ECs
MT PCs
d
MT
rpr
hid
grim
mRNA fold change (RT-PCR)
p=0.4814
p=0.1108
p=0.0008
MT^ts>
MT^ts>Myc
e
MT
Myc
mRNA fold change (RT-PCR)
p=0.0003
Virgins
Mated
f
DAPI
pH3
100µm
MT^ts>Myc^RNAi
Mate
g
Cell Proliferation
pH3 count per gut
p<0.0001
p=0.0001
h
DAPI
GFP
pH3
100µm
EGT^ts>yki^[S3A]
MT^ts>Myc^RNAi
i
Width - day3
Width (Fold change to Cont.)
p=0.8167
p=0.5693
j
GFP Intensity - day 3
GFP Signal Intensity (Fold change to Cont.)
p<0.0001
p<0.0001
k
Proliferation - day 3
pH3 count per gut
p<0.0001
p=0.0014
EGT^ts>, MT^ts>
EGT^ts>yki^[S3A], MT^ts>
EGT^ts>yki^[S3A], MT^ts>Myc^RNAi
m
Width - day 5
Width (Fold change to Cont.)
p=0.0460
p=0.1648
n
GFP Intensity - day 5
GFP Signal Intensity (Fold change to Cont.)
p<0.0001
p=0.0064
o
Proliferation - day 5
pH3 count per gut
p<0.0001
p=0.0386
p
Bloating - day 6
Abdomen/Head Ratio
p<0.0001
p=0.0001
EGT^ts>, MT^ts>
EGT^ts>yki^[S3A], MT^ts>
EGT^ts>yki^[S3A], MT^ts>Myc^RNAi#2
l
DAPI
GFP
pH3
100µm
EGT^ts>yki^[S3A]
MT^ts>Myc^RNAi#2
q
Jra
ovo
Ets21C
exex
tx
kay
ttk
luna
Myc
nub
Hr39
Pdp1
ci
cad
cwo
dsx
GATAe
Ptx1
ERR
Average Log2FC

Fig. S4 | Related to Figure 5. a, Log2 expression of *Myc* in gut ISCs, gut ECs and MT PCs of control and Yki flies; data from published Yki MT snRNA-seq dataset. **b,** Differential regulon activity analysis of transcription factors in the MTs with published Yki fly snRNA-seq data. A high Log2fc corresponds to increased activity/expression of positively regulated target genes of the regulon in Yki versus control. **c,** Log2 expression of *dPANK4*, *Fbl*, and *Smvt* in gut ISCs, gut ECs and MT PCs of control and Yki flies; data from published Yki snRNA-seq dataset. **d,** qRT-PCR analysis of apoptosis genes (*rpr, hid,* and *grim*) mRNA levels in MTs from control and MT-specific *Myc* overexpression flies at day 7. n = 3. **e,** qRT-PCR analysis of *Myc* in the MTs of virgin or mated female flies. **f, g,** Representative images (**f**) and quantification (**g**) of pH3+ cells per midgut in virgin or mated female flies with or without MT *Myc* knockdown at day 10. In the pH3 panel, the guts are outlined with dashed lines. n = 6, 7 and 7 in MT^{ts}> (virgins), MT^{ts}> (mated), and MT^{ts}>*Myc-RNAi* (mated), respectively. **h,** Representative images of guts from control flies and Yki flies with or without MT-specific *Myc* knockdown at day 3. **i-k,** Quantification of gut width (**i**), GFP intensity (**j**), and pH3+ cell counts (**k**). n = 5, 8, and 6 in EGT^{ts}>, MT^{ts}>, EGT^{ts}>$yki^{[S3A]}$,MT^{ts}>, and EGT^{ts}>$yki^{[S3A]}$, MT^{ts}>*Myc-RNAi* in **i**, respectively. n = 5, 8, and 6 in EGT^{ts}>, MT^{ts}>, EGT^{ts}>$yki^{[S3A]}$,MT^{ts}>, and EGT^{ts}>$yki^{[S3A]}$, MT^{ts}>*Myc-RNAi* in **j**, respectively. n = 6, 9, and 9 in EGT^{ts}>, MT^{ts}>, EGT^{ts}>$yki^{[S3A]}$,MT^{ts}>, and EGT^{ts}>$yki^{[S3A]}$, MT^{ts}>*Myc-RNAi* in **k**, respectively. **l,** Representative images of guts and representative images of bloating phenotypes from control flies and Yki flies with or without MT-specific *Myc* knockdown at day 5 using independent fly lines. **m-o,** Quantification of gut width (**m**), GFP intensity (**n**), and pH3+ cell counts (**o**). n = 4, 5, and 5 in EGT^{ts}>, MT^{ts}>, EGT^{ts}>$yki^{[S3A]}$,MT^{ts}>, and EGT^{ts}>$yki^{[S3A]}$, MT^{ts}>*Myc-RNAi-#2* in **m**, respectively. n = 5, 5, and 5 in EGT^{ts}>, MT^{ts}>, EGT^{ts}>$yki^{[S3A]}$,MT^{ts}>, and EGT^{ts}>$yki^{[S3A]}$, MT^{ts}>*Myc-RNAi-#2* in **n**, respectively. n = 7, 10, and 10 in EGT^{ts}>, MT^{ts}>, EGT^{ts}>$yki^{[S3A]}$,MT^{ts}>, and EGT^{ts}>$yki^{[S3A]}$, MT^{ts}>*Myc-RNAi-#2* in **o**, respectively. **p,** Bloating quantification via abdomen-to-head ratio from panel **l**. n = 12, 12, and 10 in EGT^{ts}>, MT^{ts}>, EGT^{ts}>$yki^{[S3A]}$,MT^{ts}>, and EGT^{ts}>$yki^{[S3A]}$, MT^{ts}>*Myc-RNAi-#2*, respectively. **q,** Differential regulon activity analysis of transcription factors in MTs with published snRNA-seq data. A high Log2fc corresponds to increased activity/expression of positively regulated target genes of the regulon in MT-specific Pvr activation versus control. Statistical significance assessed by wilcox test (**b, q**), unpaired two-sided Student's *t*-test (**d, e**) and one-way ANOVA (**g, i-k, m-p**). Error bars indicate s.d., with means at the center. Source data are provided as a Source Data file.

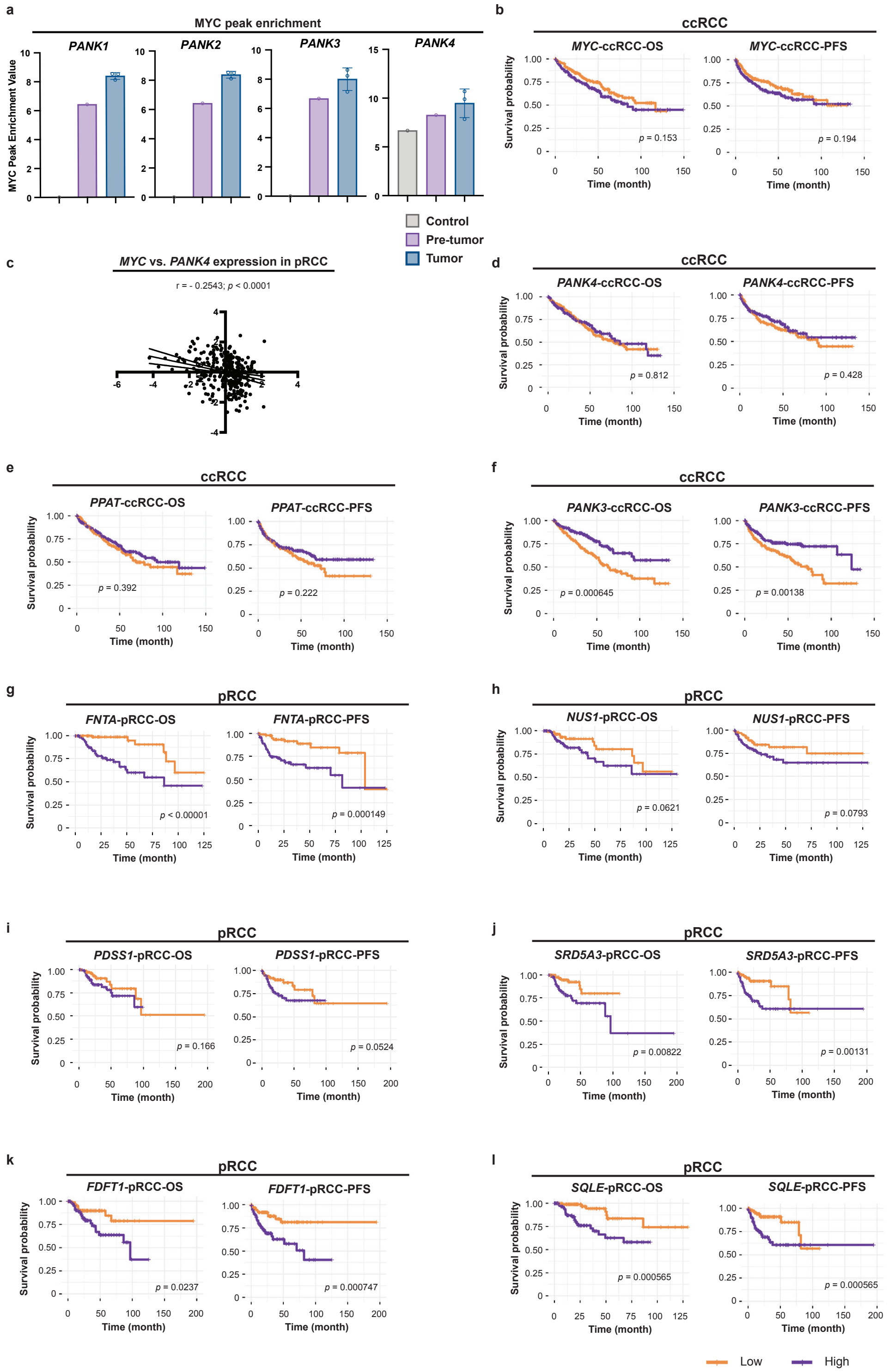
a
MYC peak enrichment
PANK1
PANK2
PANK3
PANK4
MYC Peak Enrichment Value
Control
Pre-tumor
Tumor
b
ccRCC
MYC-ccRCC-OS
MYC-ccRCC-PFS
p = 0.153
p = 0.194
c
MYC vs. PANK4 expression in pRCC
r = - 0.2543; p < 0.0001
d
ccRCC
PANK4-ccRCC-OS
PANK4-ccRCC-PFS
p = 0.812
p = 0.428
e
ccRCC
PPAT-ccRCC-OS
PPAT-ccRCC-PFS
p = 0.392
p = 0.222
f
ccRCC
PANK3-ccRCC-OS
PANK3-ccRCC-PFS
p = 0.000645
p = 0.00138
g
pRCC
FNTA-pRCC-OS
FNTA-pRCC-PFS
p < 0.00001
p = 0.000149
h
pRCC
NUS1-pRCC-OS
NUS1-pRCC-PFS
p = 0.0621
p = 0.0793
i
pRCC
PDSS1-pRCC-OS
PDSS1-pRCC-PFS
p = 0.166
p = 0.0524
j
pRCC
SRD5A3-pRCC-OS
SRD5A3-pRCC-PFS
p = 0.00822
p = 0.00131
k
pRCC
FDFT1-pRCC-OS
FDFT1-pRCC-PFS
p = 0.0237
p = 0.000747
l
pRCC
SQLE-pRCC-OS
SQLE-pRCC-PFS
p = 0.000565
p = 0.000565
Survival probability
Time (month)
Low
High

Fig. S5 | Related to Figure 6. a, MYC peak enrichment values in the promoter regions of PANK1-4 from published ChIP-seq datasets. Enrichment is calculated as Log2(ChIP - Input), where ChIP and Input represent the number of reads in the peak region normalized by total library size (in millions). A value of 0 is assigned to promoters with no peak called. **b,** Kaplan–Meier (KM) survival analyses of overall survival (OS) and progression-free survival (PFS) in 336 Clear Cell Renal Cell Carcinoma (ccRCC/KIRC) patients comparing low (bottom third) versus high (top third) expression of *MYC*. **c,** Correlation plot showing the negative relationship between MYC and PANK4 expression in 280 Papillary Renal Cell Carcinoma (pRCC/KIRP) patients from the TCGA PanCancer Atlas. **d-f,** KM survival analyses of OS and PFS in 512 ccRCC/KIRC patients comparing low (bottom third) versus high (top third) expression of *PANK4* (**d**), and *PANK3* (**e**), and *PPAT* (**f**); data from the TCGA PanCancer Atlas. **g-l,** KM survival analyses (OS and PFS) for 188 pRCC patients stratified by expression of *FNTA* (**g**), *NUS1* (**h**), *PDSS1* (**i**), *SRD5A3* (**j**), *FDFT1* (**k**), and *SQLE* (**l**), comparing bottom versus top expression tertiles. Statistical significance assessed by Pearson correlation analysis (**b**), and log-rank (Mantel-Cox) test for survival curves (**c-l**). Source data are provided as a Source Data file.

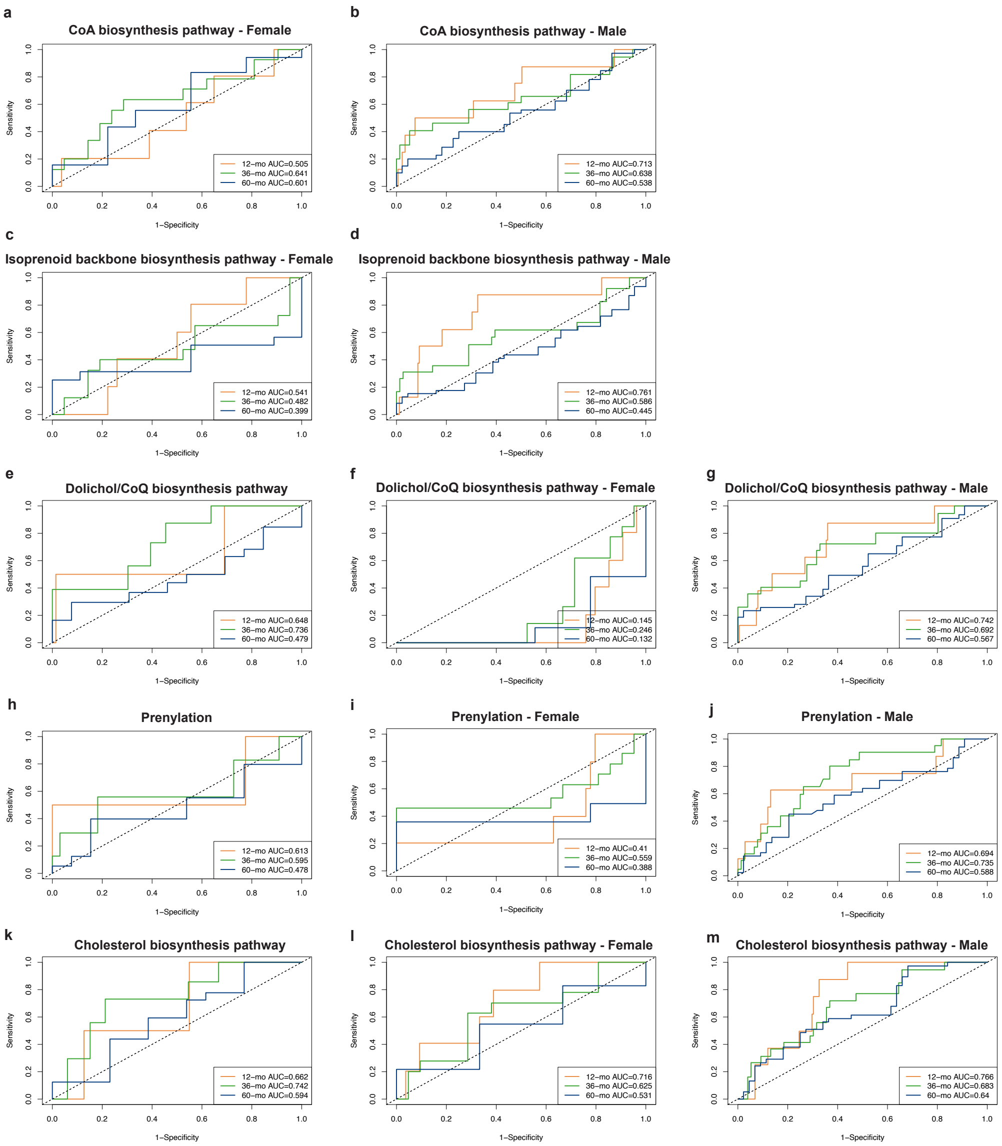
a
CoA biosynthesis pathway - Female
b
CoA biosynthesis pathway - Male
c
Isoprenoid backbone biosynthesis pathway - Female
d
Isoprenoid backbone biosynthesis pathway - Male
e
Dolichol/CoQ biosynthesis pathway
f
Dolichol/CoQ biosynthesis pathway - Female
g
Dolichol/CoQ biosynthesis pathway - Male
h
Prenylation
i
Prenylation - Female
j
Prenylation - Male
k
Cholesterol biosynthesis pathway
l
Cholesterol biosynthesis pathway - Female
m
Cholesterol biosynthesis pathway - Male
Sensitivity
1−Specificity
12−mo AUC=0.505
36−mo AUC=0.641
60−mo AUC=0.601
12−mo AUC=0.713
36−mo AUC=0.638
60−mo AUC=0.538
12−mo AUC=0.541
36−mo AUC=0.482
60−mo AUC=0.399
12−mo AUC=0.761
36−mo AUC=0.586
60−mo AUC=0.445
12−mo AUC=0.648
36−mo AUC=0.736
60−mo AUC=0.479
12−mo AUC=0.145
36−mo AUC=0.246
60−mo AUC=0.132
12−mo AUC=0.742
36−mo AUC=0.692
60−mo AUC=0.567
12−mo AUC=0.613
36−mo AUC=0.595
60−mo AUC=0.478
12−mo AUC=0.41
36−mo AUC=0.559
60−mo AUC=0.388
12−mo AUC=0.694
36−mo AUC=0.735
60−mo AUC=0.588
12−mo AUC=0.662
36−mo AUC=0.742
60−mo AUC=0.594
12−mo AUC=0.716
36−mo AUC=0.625
60−mo AUC=0.531
12−mo AUC=0.766
36−mo AUC=0.683
60−mo AUC=0.64

Fig. S6 | Related to Figure 6. a-d, Time-dependent ROC (receiver operating characteristic) curve analyses showing AUC (area under the curve) values at 12, 24, and 36 months of metastasis stage M0. Panels **a-b** show AUC for CoA biosynthesis gene signatures in female (**m**) (n = 72) and male (**n**) (n = 208) pRCC patients; panels **c-d** show AUC for isoprenoid backbone biosynthesis gene signatures in female (**o**) (n = 72) and male (**p**) (n = 208) patients. Pathway gene sets include: CoA biosynthesis: *PANK1-4, PPCS, PPCDC, PPAT* and *COASY*; isoprenoid backbone synthesis: *MVK, PMVK, MVD, IDI1, IDI2, FDPS* and *GGPS1*. **e-m,** Time-dependent ROC analysis of dolichol and ubiquinone (CoQ) biosynthesis gene signatures in pRCC patients showing AUC performance across both genders (**e**) (n = 91), females (**f**) (n = 72), and males (**g**) (n = 208). Pathway gene sets include: *DHDDS, NUS1, SRD5A1* and *PDSS1/2*. **h-j,** Time-dependent ROC analysis of protein prenylation gene signatures in pRCC patients showing AUC performance across both genders (**h**) (n = 91), females (**i**) (n = 72), and males (**j**) (n = 208). Pathway gene sets include: *FNTA, FNTB* and *PGGT1B*. **k-m,** Time-dependent ROC analysis of cholesterol biosynthesis gene signatures in pRCC patients showing AUC performance across both genders (**k**) (n = 91), females (**l**) (n = 72), and males (**m**) (n = 208). Pathway gene sets include: *FDFT1, SQLE, LSS, CYP51A1* and *DHCG7*. Statistical significance assessed by AUC analyses (**a-m**). Source data are provided as a Source Data file.

**Supplementary table1. Information about vitamin supplementation**		
	Supplier and Catalog number	Final concentration
Vitamin B1 (thiamine)	Sigma, T4625	0.2 mM
Vitamin B2 (riboflavin)	Sigma, R4500	0.1 mM
Vitamin B3 (nicotinic acid)	Sigma, N4126	5 mM
Vitamin B5 (pantothenate acid)	Sigma, 21210	2.5 mM
Vitamin B6 (pyridoxine)	Sigma, P9755	0.5 mM
Vitamin B7 (Biotin)	Sigma, B4501	0.01 mM

Supplementary table2. Transcription factor prediction

Query gene	TF FBgn	TF Symbol	Peak Count	Motif Count	Location	Protein-Protein Interactors	Genetic Interactors
ppcs	FBgn0259750	ab	0	3	upstream		
Ppcdc	FBgn0259750	ab	0	1	upstream		
fbl	FBgn0259750	ab	0	1	upstream		
Ppat-Dpck	FBgn0000022	ac	0	2	upstream	da	sc, h (pubmed)
Ppat-Dpck	FBgn0000413	ac	0	6	upstream		
CG5828	FBgn0000022	ac	0	2	upstream	da	sc, h (pubmed)
fbl	FBgn0000022	ac	0	1	upstream		sc, ttk (pubmed)
Ppat-Dpck	FBgn0005694	Aef1	0	2	upstream		
Ppcdc	FBgn0003270	amos	0	1	upstream		da (pubmed)
ppcs	FBgn0000097	aop	0	1	upstream		pnt, ttk (pubmed)
Ppcdc	FBgn0000097	aop	0	1	upstream		H (pubmed)
CG5828	FBgn0000097	aop	0	1	upstream		H, pnt (pubmed)
Ppat-Dpck	FBgn0000137	ase	0	2	upstream	da	
CG5828	FBgn0000137	ase	0	2	upstream	da	
fbl	FBgn0000137	ase	0	1	upstream		
ppcs	FBgn0004870	bab1	0	3	upstream		
Ppcdc	FBgn0004870	bab1	0	7	upstream		
Ppat-Dpck	FBgn0004870	bab1	0	1	upstream		
Dpck	FBgn0004870	bab1	0	2	upstream		
CG5828	FBgn0004870	bab1	0	2	upstream		
fbl	FBgn0004870	bab1	0	2	upstream		
Ppcdc	FBgn0015602	BEAF-32	0	1	upstream	Dref	
CG5828	FBgn0015602	BEAF-32	0	2	upstream	Dref	
fbl	FBgn0015602	BEAF-32	0	1	upstream	Dref	
ppcs	FBgn0045759	bin	0	2	upstream		

ppcs	FBgn0045759	bin	0	2	intron		
Dpck	FBgn0045759	bin	0	1	upstream	prd, D	
CG5828	FBgn0045759	bin	1	1	upstream	D	
fbl	FBgn0045759	bin	0	4	intron	D	
fbl	FBgn0045759	bin	0	1	upstream	D	
ppcs	FBgn0035625	Blimp-1	0	8	upstream		
ppcs	FBgn0035625	Blimp-1	0	4	intron		
CG5828	FBgn0035625	Blimp-1	0	1	upstream		
ppcs	FBgn0004893	bowl	0	1	upstream		
CG5828	FBgn0004893	bowl	0	1	upstream		
ppcs	FBgn0283451	br	2	0	upstream	Rel, rib	Met (pubmed)
Ppat-Dpck	FBgn0283451	br	1	0	upstream	rib	
Dpck	FBgn0283451	br	1	0	upstream	rib	
Dpck	FBgn0000210	br	0	1	upstream		
CG5828	FBgn0283451	br	1	0	upstream	rib	
fbl	FBgn0283451	br	1	0	upstream	rib	
fbl	FBgn0000210	br	0	4	intron		
ppcs	FBgn0000210	br-PE	0	1	upstream		
ppcs	FBgn0263108	BtbVII	0	2	upstream		
ppcs	FBgn0263108	BtbVII	0	1	intron		
Dpck	FBgn0263108	BtbVII	0	1	upstream		
CG5828	FBgn0263108	BtbVII	0	1	upstream		
fbl	FBgn0263108	BtbVII	0	1	upstream		
fbl	FBgn0263108	BtbVII	0	1	intron		
Ppat-Dpck	FBgn0025679	Bteb2	0	2	upstream		
fbl	FBgn0025679	Bteb2	0	3	upstream		
Ppat-Dpck	FBgn0000286	Cf2-PA	0	1	upstream		

Dpck	FBgn0000286	Cf2-PA	0	2	upstream	bin	
fbl	FBgn0000286	Cf2-PA	0	1	upstream	bin	
ppcs	FBgn0000286	Cf2-PB	0	8	upstream	bin	
fbl	FBgn0000286	Cf2-PB	0	1	intron	bin	
ppcs	FBgn0037446	CG10267	0	2	upstream	Dll, toy	
Ppat-Dpck	FBgn0037446	CG10267	0	2	upstream	Dll	
fbl	FBgn0037446	CG10267	0	2	upstream		
Ppcdc	FBgn0034945	CG10904	0	1	upstream		
fbl	FBgn0030532	CG11071	0	1	upstream		
ppcs	FBgn0035454	CG12029	0	3	intron		
Ppcdc	FBgn0035454	CG12029	0	1	upstream		
Ppat-Dpck	FBgn0035454	CG12029	0	1	upstream		
Dpck	FBgn0035454	CG12029	0	3	upstream		
CG5828	FBgn0035454	CG12029	0	3	upstream		
fbl	FBgn0035454	CG12029	0	2	upstream		
CG5828	FBgn0029957	CG12155	0	1	upstream	Trl	
fbl	FBgn0029822	CG12236	0	1	intron		
ppcs	FBgn0037206	CG12768	0	1	intron	Mes2	
Dpck	FBgn0037206	CG12768	0	1	upstream		
Ppcdc	FBgn0035160	CG13897	0	2	upstream		
Ppat-Dpck	FBgn0035160	CG13897	0	1	upstream		
fbl	FBgn0035160	CG13897	0	1	upstream		
Ppat-Dpck	FBgn0035407	CG14962	0	3	upstream		
Dpck	FBgn0003715	CG16778	0	1	upstream		
CG5828	FBgn0003715	CG16778	0	1	upstream		
fbl	FBgn0003715	CG16778	0	2	intron		
ppcs	FBgn0037735	CG16899	0	1	upstream		

ppcs	FBgn0037735	CG16899	0	1	intron		
Dpck	FBgn0037735	CG16899	0	1	upstream		
CG5828	FBgn0037735	CG16899	0	1	upstream		
fbl	FBgn0037735	CG16899	0	3	intron		
fbl	FBgn0037735	CG16899	0	1	upstream		
Ppat-Dpck	FBgn0035144	CG17181	0	1	upstream	Aef1	
CG5828	FBgn0035144	CG17181	0	1	upstream	pnt	
ppcs	FBgn0039905	CG2052	0	3	upstream		
ppcs	FBgn0039905	CG2052	0	4	intron		
Ppcdc	FBgn0039905	CG2052	0	1	intron		
Ppat-Dpck	FBgn0039905	CG2052	0	3	upstream		
Dpck	FBgn0039905	CG2052	0	3	upstream		
CG5828	FBgn0039905	CG2052	0	11	upstream		
fbl	FBgn0039905	CG2052	0	2	upstream		
Ppat-Dpck	FBgn0034946	CG3065	0	3	upstream		
Dpck	FBgn0034946	CG3065	0	8	upstream		
CG5828	FBgn0034946	CG3065	0	4	upstream		
fbl	FBgn0034946	CG3065	0	4	upstream		
Ppcdc	FBgn0031375	CG31670	0	1	upstream		klu (pubmed)
Dpck	FBgn0031375	CG31670	0	4	upstream		klu, Doc2 (pubmed)
fbl	FBgn0031375	CG31670	0	2	intron		
ppcs	FBgn0052105	CG32105	0	1	upstream		
CG5828	FBgn0053980	CG33980	0	1	upstream		
Dpck	FBgn0031573	CG3407	0	1	upstream		
ppcs	FBgn0036423	CG3919	0	1	intron		
Ppat-Dpck	FBgn0036423	CG3919	0	1	upstream		
fbl	FBgn0036423	CG3919	0	2	upstream		

fbl	FBgn0036423	CG3919	0	1	intron		
CG5828	FBgn0038787	CG4360	0	1	upstream		
Ppat-Dpck	FBgn0038787	CG4360-F1-3	0	4	upstream		
ppcs	FBgn0030432	CG4404	0	1	intron		
Ppcdc	FBgn0030432	CG4404	0	2	upstream		
fbl	FBgn0030432	CG4404	0	1	upstream		
ppcs	FBgn0038766	CG4854	0	2	intron		
Ppcdc	FBgn0038766	CG4854	0	1	upstream		
Ppat-Dpck	FBgn0038766	CG4854	0	2	upstream		
CG5828	FBgn0043457	CG5180	0	1	upstream		
ppcs	FBgn0039169	CG5669	0	1	upstream		
Dpck	FBgn0039169	CG5669	0	6	upstream		
CG5828	FBgn0039169	CG5669	0	3	upstream		
fbl	FBgn0039169	CG5669	0	3	upstream		
ppcs	FBgn0032587	CG5953	0	1	upstream	knrl, sens	
Ppcdc	FBgn0032587	CG5953	0	1	upstream	sens	
Dpck	FBgn0032587	CG5953	0	2	upstream	knrl	
CG5828	FBgn0032587	CG5953	0	1	upstream		
fbl	FBgn0032587	CG5953	0	2	intron		
ppcs	FBgn0038316	CG6276	0	2	upstream		
ppcs	FBgn0038316	CG6276	0	1	intron		
Ppcdc	FBgn0038316	CG6276	0	1	upstream		
Dpck	FBgn0038316	CG6276	0	2	upstream		
CG5828	FBgn0038316	CG6276	0	2	upstream		
fbl	FBgn0038316	CG6276	0	1	upstream		
Ppat-Dpck	FBgn0036179	CG7368	0	1	upstream		
ppcs	FBgn0033616	CG7745	0	2	upstream		

ppcs	FBgn0033616	CG7745	0	1	intron		
Dpck	FBgn0033616	CG7745	0	1	upstream		
CG5828	FBgn0033616	CG7745	0	1	upstream		
Ppat-Dpck	FBgn0039740	CG7928	0	1	upstream		
ppcs	FBgn0035824	CG8281	0	1	upstream		
ppcs	FBgn0035824	CG8281	0	1	intron		
fbl	FBgn0037722	CG8319	0	2	upstream		
Ppcdc	FBgn0036900	CG8765	0	1	upstream		
CG5828	FBgn0036900	CG8765	0	1	upstream		
fbl	FBgn0036900	CG8765	0	2	intron		
ppcs	FBgn0034810	CG9895	0	2	intron		
Ppat-Dpck	FBgn0034810	CG9895	0	1	upstream		
Dpck	FBgn0034810	CG9895	0	6	upstream		
CG5828	FBgn0034810	CG9895	0	3	upstream		
fbl	FBgn0034810	CG9895	0	2	upstream		
ppcs	FBgn0086758	chinmo	0	3	intron		
Ppcdc	FBgn0086758	chinmo	0	2	upstream		
CG5828	FBgn0086758	chinmo	0	1	upstream		
fbl	FBgn0086758	chinmo	0	1	intron		
ppcs	FBgn0000370	Crc	0	3	upstream	EcR	EcR (pubmed)
ppcs	FBgn0036126	Crc	0	2	upstream	Xrp1, crc	
Ppat-Dpck	FBgn0000370	Crc	0	1	upstream	EcR	EcR (pubmed)
ppcs	FBgn0014143	croc	0	1	upstream	opa	
ppcs	FBgn0014143	croc	0	1	intron	opa	
Ppcdc	FBgn0014143	croc	0	2	upstream		
Ppat-Dpck	FBgn0014143	croc	0	1	upstream		
Dpck	FBgn0014143	croc	0	1	upstream	prd	

fbl	FBgn0014143	croc	0	1	upstream		
ppcs	FBgn0020309	crol-F7-16	0	1	upstream		
CG5828	FBgn0020309	crol-F7-16	0	1	upstream		
CG5828	FBgn0001994	crp	0	1	upstream		
ppcs	FBgn0023094	cyc	1	0	intron		
Ppcdc	FBgn0023094	cyc	1	0	upstream		
Ppat-Dpck	FBgn0023094	cyc	1	0	upstream		
Dpck	FBgn0023094	cyc	3	0	upstream		
CG5828	FBgn0023094	cyc	2	0	upstream		
fbl	FBgn0023094	cyc	4	0	intron		
Ppcdc	FBgn0000411	D	3	0	upstream	vnd	
Ppat-Dpck	FBgn0000411	D	3	0	upstream		
Dpck	FBgn0000411	D	2	0	upstream	Doc1, bin	
CG5828	FBgn0000411	D	2	0	upstream	bin	
fbl	FBgn0000411	D	1	0	upstream	vnd, ftz-f1, ttk, bin	
ppcs	FBgn0022935	D19A	0	2	upstream		
Dpck	FBgn0022935	D19A	0	1	upstream		
CG5828	FBgn0022935	D19A	0	1	upstream		
fbl	FBgn0022935	D19A	0	1	upstream		
fbl	FBgn0022935	D19A	0	1	intron		
ppcs	FBgn0022699	D19B-F10-12	0	1	intron		
ppcs	FBgn0267821	da	1	0	upstream	ey, Fer3	
ppcs	FBgn0000413	da	0	1	upstream		
ppcs	FBgn0000413	da	0	4	intron		
Ppcdc	FBgn0267821	da	1	0	upstream	dimm, ey, Fer3	amos (pubmed)
Ppat-Dpck	FBgn0267821	da	3	0	upstream	ac, sc, l(1)sc, ase, HLH54F, ey, Fer3	sc, l(1)sc (pubmed)
CG5828	FBgn0000413	da	0	4	upstream		

CG5828	FBgn0267821	da	2	0	upstream	ac, sc, l(1)sc, ase, Fer3	sc, l(1)sc (pubmed)
Ppcdc	FBgn0000413	dei	0	5	upstream		
Ppcdc	FBgn0008649	dei	0	2	upstream		
Ppcdc	FBgn0023091	dimm	0	1	upstream	da, sqz	
Dpck	FBgn0000413	dimm	0	1	upstream		
Dpck	FBgn0023091	dimm	0	2	upstream	sqz	
ppcs	FBgn0040465	Dip3	0	1	upstream	CG4854	
Ppcdc	FBgn0040465	Dip3	0	1	intron	dl, CG4854	
Ppat-Dpck	FBgn0040465	Dip3	0	2	upstream	CG4854	
Dpck	FBgn0040465	Dip3	0	2	upstream		
CG5828	FBgn0040465	Dip3	0	2	upstream		
Ppcdc	FBgn0000462	dl	0	2	upstream		
ppcs	FBgn0000157	Dll	1	0	upstream	Dref	
Ppat-Dpck	FBgn0000157	Dll	2	0	upstream	Dref	
CG5828	FBgn0000157	Dll	1	0	upstream	Dref	
Dpck	FBgn0028789	Doc1	0	1	intron	Doc2, D	Doc2 (pubmed)
Dpck	FBgn0035956	Doc2	0	1	intron	Doc1	Doc1 (pubmed)
Dpck	FBgn0035954	Doc3	0	1	intron		
Ppat-Dpck	FBgn0010109	dpn	0	2	upstream	h, Hey	
ppcs	FBgn0000492	Dr	0	1	upstream		
ppcs	FBgn0015664	Dref	0	2	upstream	Dll	
ppcs	FBgn0015664	Dref	0	1	intron	Dll	
Ppcdc	FBgn0015664	Dref	0	1	upstream	BEAF-32	
Ppat-Dpck	FBgn0015664	Dref	0	3	upstream	Dll	
CG5828	FBgn0015664	Dref	0	2	upstream	BEAF-32, Dll	
fbl	FBgn0015664	Dref	0	1	upstream	BEAF-32	
ppcs	FBgn0015381	dsf	0	1	upstream		

Ppat-Dpck	FBgn0000504	dsx-F	0	1	upstream	ey	
CG5828	FBgn0000504	dsx-F	0	1	upstream		
ppcs	FBgn0039411	dys	0	4	upstream	tgo	
Dpck	FBgn0039411	dys	0	1	upstream	tgo	
CG5828	FBgn0015014	dys	0	1	upstream		
CG5828	FBgn0039411	dys	0	2	upstream		
Ppcdc	FBgn0000591	E(spl)	0	1	upstream	da, h	Dl, H (pubmed)
ppcs	FBgn0000546	EcR	0	1	upstream	usp, Met, tai, crc	Hr39, crc (pubmed)
ppcs	FBgn0000546	EcR	0	1	intron	usp, Met, tai, crc	Hr39, crc (pubmed)
Ppat-Dpck	FBgn0000546	EcR	2	0	upstream	usp, crc	crc (pubmed)
ppcs	FBgn0000560	eg	0	2	upstream		
ppcs	FBgn0000568	Eip75B	0	2	intron	Hr51	Kr (pubmed)
Dpck	FBgn0000568	Eip75B	0	1	upstream		
fbl	FBgn0000568	Eip75B	0	2	upstream	Hr51	
ppcs	FBgn0004865	Eip78C	0	1	upstream		
ppcs	FBgn0004865	Eip78C	0	1	intron		
ppcs	FBgn0013948	Eip93F	0	3	upstream		
ppcs	FBgn0013948	Eip93F	0	1	intron		
Dpck	FBgn0013948	Eip93F	0	1	upstream		
CG5828	FBgn0013948	Eip93F	0	2	upstream		
ppcs	FBgn0035849	ERR	0	1	intron		
Ppat-Dpck	FBgn0001981	esg-F3-5	0	2	upstream		ase (pubmed)
CG5828	FBgn0001981	esg-F3-5	0	3	upstream	Sp1	ase (pubmed)
fbl	FBgn0001981	esg-F3-5	0	2	upstream	Sp1	ase (pubmed)
ppcs	FBgn0000591	Espl	0	2	intron	da	
Ppat-Dpck	FBgn0000591	Espl	0	3	upstream	da, dpn, h	l(1)sc, H (pubmed)
Dpck	FBgn0000591	Espl	0	1	upstream	h	H (pubmed)

fbl	FBgn0000591	Espl	0	2	intron		l(1)sc (pubmed)
ppcs	FBgn0005660	Ets21c	0	2	upstream		
Ppcdc	FBgn0005660	Ets21c	0	1	upstream		
CG5828	FBgn0005660	Ets21c	0	2	upstream		
ppcs	FBgn0005658	Ets65A	0	1	upstream		
Ppcdc	FBgn0005658	Ets65A	0	1	upstream		
CG5828	FBgn0005658	Ets65A	0	1	upstream		
ppcs	FBgn0039225	Ets96B	0	1	upstream		
CG5828	FBgn0039225	Ets96B	0	1	upstream		
ppcs	FBgn0004510	Ets97D	0	2	upstream	Myc	
CG5828	FBgn0004510	Ets97D	0	2	upstream	Myc	
fbl	FBgn0005659	Ets98B	0	1	upstream		
ppcs	FBgn0005558	ey	0	3	intron	da	toy (pubmed)
Ppcdc	FBgn0005558	ey	0	1	upstream	da, hth	toy (pubmed)
Ppat-Dpck	FBgn0005558	ey	0	2	upstream	da, hth	
fbl	FBgn0005558	ey	0	1	upstream		
ppcs	FBgn0037475	Fer1	0	2	intron		
Ppcdc	FBgn0037475	Fer1	0	2	upstream		
CG5828	FBgn0037475	Fer1	0	2	upstream		
ppcs	FBgn0038402	Fer2	0	1	upstream		
CG5828	FBgn0038402	Fer2	0	2	upstream		
ppcs	FBgn0037937	Fer3	1	0	upstream	da	
Ppcdc	FBgn0037937	Fer3	0	2	upstream	da	
Ppat-Dpck	FBgn0037937	Fer3	2	0	upstream	da	
CG5828	FBgn0037937	Fer3	1	0	upstream	da	
ppcs	FBgn0000659	fkh	0	1	upstream		
ppcs	FBgn0000659	fkh	0	1	intron		

Dpck	FBgn0000659	fkh	0	3	upstream		
fbl	FBgn0000659	fkh	0	2	intron		
fbl	FBgn0001078	ftz-f1	0	1	upstream	D	
ppcs	FBgn0032223	GATAd	0	2	upstream		
ppcs	FBgn0032223	GATAd	0	1	intron		
Ppat-Dpck	FBgn0032223	GATAd	0	1	upstream		
CG5828	FBgn0032223	GATAd	0	1	upstream		
fbl	FBgn0032223	GATAd	0	2	intron		
Ppcdc	FBgn0038391	GATAe	1	0	upstream		
ppcs	FBgn0261703	gce	0	1	upstream	Met	Met (pubmed)
Ppcdc	FBgn0261703	gce	0	1	upstream		
Ppat-Dpck	FBgn0261703	gce	0	1	upstream		
ppcs	FBgn0004618	gl	0	1	upstream		
Dpck	FBgn0004618	gl	0	2	upstream		
ppcs	FBgn0259211	grh	1	0	upstream	Rel	
Ppat-Dpck	FBgn0259211	grh	1	0	upstream		
CG5828	FBgn0259211	grh	1	0	upstream		
Dpck	FBgn0001138	grn	0	1	upstream		
Ppat-Dpck	FBgn0001148	gsb	0	1	upstream		
ppcs	FBgn0001150	gt	0	1	upstream	ttk	
Dpck	FBgn0001150	gt	0	1	upstream		
CG5828	FBgn0001150	gt	1	2	upstream		
fbl	FBgn0001150	gt	0	1	intron	ttk	
Ppcdc	FBgn0001168	h	1	0	upstream		
Ppat-Dpck	FBgn0001168	h	1	0	upstream	dpn	ac, sc, l(1)sc (pubmed)
Dpck	FBgn0001168	h	0	1	upstream		
CG5828	FBgn0001168	h	2	0	upstream		ac, sc, l(1)sc (pubmed)

Ppat-Dpck	FBgn0032209	Hand	0	2	upstream		
fbl	FBgn0032209	Hand	0	1	intron		
ppcs	FBgn0001180	hb	0	13	upstream		Kr (pubmed)
ppcs	FBgn0001180	hb	0	5	intron		Kr (pubmed)
Ppcdc	FBgn0001180	hb	0	1	upstream		
Ppcdc	FBgn0001180	hb	0	3	intron		
Ppat-Dpck	FBgn0001180	hb	0	6	upstream		
Dpck	FBgn0001180	hb	0	16	upstream		
CG5828	FBgn0001180	hb	0	19	upstream		Kr (pubmed)
fbl	FBgn0001180	hb	0	3	upstream		
fbl	FBgn0001180	hb	0	3	intron		
fbl	FBgn0001185	her	0	1	intron		
Ppcdc	FBgn0027788	Hey	0	1	upstream		
Ppat-Dpck	FBgn0027788	Hey	0	1	upstream	dpn	
ppcs	FBgn0001204	hkb	0	2	intron		
Ppcdc	FBgn0001204	hkb	0	2	upstream		
Dpck	FBgn0001204	hkb	0	2	upstream		
fbl	FBgn0261283	HLH106	0	1	intron		
Ppat-Dpck	FBgn0011277	HLH4C	0	2	upstream		
Ppat-Dpck	FBgn0022740	HLH54F	0	3	upstream	da	
Ppcdc	FBgn0002609	HLHm3	0	2	upstream	h	
Ppat-Dpck	FBgn0002609	HLHm3	0	2	upstream	ac, sc, h	
Ppcdc	FBgn0002631	HLHm5	0	1	upstream	da	
Ppat-Dpck	FBgn0002631	HLHm5	0	3	upstream	da	
Ppat-Dpck	FBgn0002633	HLHm7	0	2	upstream	ac, sc, da	
ppcs	FBgn0002733	HLHmbeta	0	1	upstream	da, eg	
Ppcdc	FBgn0002733	HLHmbeta	0	1	upstream	da, tap	

Ppat-Dpck	FBgn0002733	HLHmbeta	0	1	upstream	sc, da	sc (pubmed)
Dpck	FBgn0002733	HLHmbeta	0	2	upstream		
CG5828	FBgn0002733	HLHmbeta	0	2	upstream	sc, da	sc (pubmed)
ppcs	FBgn0002734	HLHmd	0	2	upstream		
ppcs	FBgn0002734	HLHmd	0	1	intron		
Ppcdc	FBgn0002734	HLHmdelta	0	2	upstream		
Ppat-Dpck	FBgn0002734	HLHmdelta	0	3	upstream		
ppcs	FBgn0002735	HLHmg	0	2	upstream	da	
ppcs	FBgn0002735	HLHmg	0	1	intron	da	
Ppcdc	FBgn0002735	HLHmg	0	1	upstream	da	
Ppat-Dpck	FBgn0002735	HLHmgamma	0	3	upstream	sc, da, dpn, ac	
ppcs	FBgn0004914	Hnf4	0	1	upstream		
Ppcdc	FBgn0004914	Hnf4	0	1	upstream		
CG5828	FBgn0004914	Hnf4	0	1	upstream		
ppcs	FBgn0261239	Hr39	0	1	intron		EcR (pubmed)
ppcs	FBgn0000448	Hr46	0	2	upstream	toy	
ppcs	FBgn0000448	Hr46	0	2	intron	toy	
Ppcdc	FBgn0000448	Hr46	0	2	upstream	toy	
ppcs	FBgn0034012	Hr51	0	1	intron	Eip75B	
Ppat-Dpck	FBgn0034012	Hr51	0	1	upstream		
CG5828	FBgn0034012	Hr51	0	2	upstream		
fbl	FBgn0034012	Hr51	0	1	upstream	Eip75B	
ppcs	FBgn0015239	Hr78	0	1	upstream		
Ppcdc	FBgn0015239	Hr78	0	1	upstream		
Ppat-Dpck	FBgn0015239	Hr78	1	0	upstream		
CG5828	FBgn0015239	Hr78	1	1	upstream		
Dpck	FBgn0037436	Hr83	0	2	upstream	kni	

fbl	FBgn0037436	Hr83	0	1	intron		
Ppcdc	FBgn0001235	hth	1	0	upstream	ey	
Ppat-Dpck	FBgn0001235	hth	1	0	upstream	ey	
ppcs	FBgn0039350	jigr1	0	2	upstream		
Ppat-Dpck	FBgn0039350	jigr1	0	2	upstream		
Dpck	FBgn0039350	jigr1	0	2	upstream		
CG5828	FBgn0039350	jigr1	0	2	upstream		
fbl	FBgn0039350	jigr1	0	1	intron		
ppcs	FBgn0027339	jim	0	6	upstream		
Ppcdc	FBgn0027339	jim	0	1	upstream		
Ppat-Dpck	FBgn0027339	jim	0	3	upstream		
Dpck	FBgn0027339	jim	0	1	upstream		
CG5828	FBgn0027339	jim	0	15	upstream		
fbl	FBgn0027339	jim	0	4	upstream		
fbl	FBgn0001291	Jra	0	1	intron	kay	kay (pubmed)
ppcs	FBgn0001291	kay	0	3	upstream	Stat92E, kay	pnt, kay (pubmed)
ppcs	FBgn0001297	kay	0	3	upstream	vri	
Dpck	FBgn0001291	kay	0	1	upstream	kay	kay (pubmed)
Dpck	FBgn0001297	kay	0	1	upstream		
fbl	FBgn0001297	kay	0	1	intron	vri, Jra	Jra (pubmed)
ppcs	FBgn0011236	ken	0	2	upstream		
ppcs	FBgn0011236	ken	0	1	intron		
Ppcdc	FBgn0011236	ken	0	1	upstream		
CG5828	FBgn0011236	ken	0	1	upstream	Trl	
fbl	FBgn0011236	ken	0	2	upstream		
ppcs	FBgn0013469	klu	0	3	intron		
Ppcdc	FBgn0013469	klu	0	7	upstream		H (pubmed)

Dpck	FBgn0013469	klu	0	3	upstream		H (pubmed)
CG5828	FBgn0013469	klu	0	4	upstream		H (pubmed)
ppcs	FBgn0001320	kni	0	3	upstream		knrl (pubmed)
Dpck	FBgn0001320	kni	0	2	intron	Hr83	knrl (pubmed)
ppcs	FBgn0001323	knrl	0	3	upstream	CG5953	kni (pubmed)
Dpck	FBgn0001323	knrl	0	2	intron	CG5953	kni (pubmed)
ppcs	FBgn0001325	Kr	0	1	upstream		Eip75B, hb (pubmed)
CG5828	FBgn0001325	Kr	1	0	upstream		hb (pubmed)
Ppat-Dpck	FBgn0002561	l(1)sc	0	2	upstream	da	da, h (pubmed)
CG5828	FBgn0002561	l(1)sc	0	2	upstream	da	da, h (pubmed)
fbl	FBgn0002561	l(1)sc	0	1	upstream		
ppcs	FBgn0086910	l(3)neo38	0	1	upstream		
ppcs	FBgn0086910	l(3)neo38	0	1	intron		
Ppat-Dpck	FBgn0086910	l(3)neo38	0	1	upstream		
CG5828	FBgn0040918	Lag1	0	1	upstream		
ppcs	FBgn0039039	lmd	0	2	upstream		
CG5828	FBgn0039039	lmd	0	2	upstream		
Ppcdc	FBgn0005630	lola	0	1	upstream		
Dpck	FBgn0005630	lola-PJ	0	1	upstream		
Dpck	FBgn0040765	luna	0	2	upstream		
CG5828	FBgn0040765	luna	0	1	upstream		
ppcs	FBgn0017578	Max	0	1	upstream	Myc	Myc (pubmed)
Ppat-Dpck	FBgn0017578	Max	0	1	upstream	Myc	Myc (pubmed)
Dpck	FBgn0023215	Max	0	1	upstream	Max	Myc (pubmed)
fbl	FBgn0023215	Max	0	1	upstream	Max	Myc (pubmed)
ppcs	FBgn0011655	Med	1	0	upstream		
Ppcdc	FBgn0011655	Med	3	0	upstream		

Ppat-Dpck	FBgn0011655	Med	3	0	upstream		
Dpck	FBgn0011655	Med	3	0	upstream		
CG5828	FBgn0011655	Med	2	0	upstream		
ppcs	FBgn0037207	Mes2	0	2	upstream	CG12768	
fbl	FBgn0037207	Mes2	0	1	upstream		
fbl	FBgn0037207	Mes2	0	1	intron		
ppcs	FBgn0002723	Met	0	1	upstream	usp, EcR, gce	gce, br (pubmed)
ppcs	FBgn0023076	Met	0	2	upstream	cyc	
ppcs	FBgn0002723	Met	0	1	intron	usp, EcR, gce	gce, br (pubmed)
ppcs	FBgn0023076	Met	0	3	intron	cyc	
ppcs	FBgn0032940	Mio	0	1	upstream		
ppcs	FBgn0039509	Mio	0	1	upstream		
Ppat-Dpck	FBgn0032940	Mio	0	1	upstream		
Ppat-Dpck	FBgn0039509	Mio	0	1	upstream		
ppcs	FBgn0262656	Myc	2	1	upstream	Max, Ets97D	Max (pubmed)
Ppcdc	FBgn0262656	Myc	1	0	upstream		
Ppat-Dpck	FBgn0262656	Myc	0	1	upstream	Max	Max (pubmed)
Dpck	FBgn0262656	Myc	2	0	upstream	Max	prd, Max (pubmed)
CG5828	FBgn0262656	Myc	1	0	upstream	Ets97D	
fbl	FBgn0262656	Myc	1	0	upstream	Max	Max (pubmed)
Ppcdc	FBgn0002922	nau	0	2	upstream		
ppcs	FBgn0030505	NFAT	0	2	intron		pnr (pubmed)
ppcs	FBgn0085424	nub	0	1	upstream	pdm2	
ppcs	FBgn0085424	nub	0	11	intron	pdm2	
ppcs	FBgn0002985	odd	0	1	upstream		
fbl	FBgn0032651	Oli	0	1	upstream		
ppcs	FBgn0003002	Opa	0	1	upstream	croc	

Ppat-Dpck	FBgn0003028	ovo	0	1	upstream		
ppcs	FBgn0004394	pdm2	0	6	intron	nub	
Ppcdc	FBgn0016694	Pdp1	0	6	upstream		
Dpck	FBgn0016694	Pdp1	0	2	upstream		
Dpck	FBgn0003053	peb-F5-7	0	1	upstream		
fbl	FBgn0003053	peb-F5-7	0	1	intron		Jra (pubmed)
Ppcdc	FBgn0002521	pho	0	1	upstream	Sp1	
Dpck	FBgn0002521	pho	0	4	upstream	Sp1	
CG5828	FBgn0002521	pho	0	1	upstream	Sp1	
fbl	FBgn0002521	pho	0	1	intron	Sp1	
ppcs	FBgn0035997	phol	0	1	intron		
Ppcdc	FBgn0035997	phol	0	2	upstream		
Dpck	FBgn0035997	phol	0	6	upstream		
fbl	FBgn0035997	phol	0	2	upstream		
ppcs	FBgn0003117	pnr	0	1	upstream	tin, tup	NFAT (pubmed)
CG5828	FBgn0003117	pnr	0	1	upstream	tup	
fbl	FBgn0003117	pnr	0	3	intron		
ppcs	FBgn0003118	pnt	0	2	upstream		aop (pubmed)
CG5828	FBgn0003118	pnt	0	2	upstream		aop (pubmed)
Dpck	FBgn0003145	prd	1	0	upstream	bin, croc	Myc (pubmed)
ppcs	FBgn0014018	Rel	0	2	intron	br, grh, sqz	
ppcs	FBgn0004795	retn	0	2	upstream		
ppcs	FBgn0004795	retn	0	2	intron		
Ppcdc	FBgn0004795	retn	0	2	upstream		
ppcs	FBgn0003254	rib	0	1	upstream	ttk, br	
ppcs	FBgn0003254	rib	0	1	intron	ttk, br	
Ppat-Dpck	FBgn0003254	rib	0	2	upstream	br	

Dpck	FBgn0003254	rib	0	2	upstream	br	
CG5828	FBgn0003254	rib	0	3	upstream	br	
fbl	FBgn0003254	rib	0	2	upstream	ttk, br	
fbl	FBgn0003254	rib	0	1	intron	ttk, br	
ppcs	FBgn0259172	rn	0	2	upstream		
Ppat-Dpck	FBgn0259172	rn	0	6	upstream		
Dpck	FBgn0259172	rn	0	2	upstream		
CG5828	FBgn0259172	rn	0	13	upstream		
ppcs	FBgn0003300	run	0	1	upstream		ttk (pubmed)
ppcs	FBgn0013753	run	0	1	upstream	run	
ppcs	FBgn0003300	run	0	1	intron		ttk (pubmed)
ppcs	FBgn0013753	run	0	1	intron	run	
Ppcdc	FBgn0003300	run	0	1	upstream	H	
Ppcdc	FBgn0013753	run	0	1	upstream	run	
Dpck	FBgn0003300	run	0	1	upstream	H	
Dpck	FBgn0013753	run	0	1	upstream	run	
fbl	FBgn0003300	run	0	1	intron		ttk (pubmed)
fbl	FBgn0013753	run	0	1	intron	run	
Ppcdc	FBgn0037672	sage	0	2	upstream		
Ppat-Dpck	FBgn0000287	salr-F3-5	0	2	upstream		
Dpck	FBgn0000287	salr-F3-5	0	2	upstream		
Ppat-Dpck	FBgn0004170	sc	0	2	upstream	da	ac, da, h (pubmed)
CG5828	FBgn0004170	sc	0	2	upstream	da	ac, da, tup, h (pubmed)
fbl	FBgn0004170	sc	0	1	upstream		ac (pubmed)
ppcs	FBgn0002573	sens	0	1	upstream	CG5953	
Ppcdc	FBgn0002573	sens	0	1	upstream	CG5953	Dl (pubmed)
ppcs	FBgn0051632	sens2	0	1	upstream		

Ppcdc	FBgn0051632	sens2	0	1	upstream		
ppcs	FBgn0003396	shn-F1-2	0	1	intron		
Ppcdc	FBgn0003396	shn-F1-2	0	1	upstream		
Ppcdc	FBgn0032741	Side	0	2	upstream		
ppcs	FBgn0005638	slbo	0	1	upstream		Stat92E (pubmed)
Ppat-Dpck	FBgn0005638	slbo	0	1	upstream		
fbl	FBgn0005638	slbo	0	2	upstream		
ppcs	FBgn0003430	slp1	0	2	upstream		
ppcs	FBgn0003430	slp1	0	1	intron		
Ppcdc	FBgn0003430	slp1	0	1	upstream		
Ppat-Dpck	FBgn0003430	slp1	0	1	upstream		
fbl	FBgn0003430	slp1	0	5	intron		
fbl	FBgn0003430	slp1	0	1	upstream		
ppcs	FBgn0004567	slp2	0	2	upstream		
ppcs	FBgn0004567	slp2	0	1	intron		
Ppcdc	FBgn0004567	slp2	0	1	upstream		
Ppat-Dpck	FBgn0004567	slp2	0	1	upstream		
fbl	FBgn0004567	slp2	0	6	intron		
fbl	FBgn0004567	slp2	0	2	upstream		
ppcs	FBgn0004892	sob	0	1	upstream		
ppcs	FBgn0005612	Sox14	0	1	intron		
Ppat-Dpck	FBgn0005612	Sox14	0	2	upstream		
ppcs	FBgn0020378	Sp1	0	1	upstream		
Ppcdc	FBgn0020378	Sp1	0	1	upstream	pho	
Dpck	FBgn0020378	Sp1	0	6	upstream	pho	
CG5828	FBgn0020378	Sp1	0	3	upstream	pho	
fbl	FBgn0020378	Sp1	0	3	upstream	pho	

ppcs	FBgn0010768	sqz	0	25	upstream	Rel	
ppcs	FBgn0010768	sqz	0	20	intron	Rel	
Ppcdc	FBgn0010768	sqz	0	1	intron	dimm	
Ppat-Dpck	FBgn0010768	sqz	0	9	upstream		
Dpck	FBgn0010768	sqz	0	23	upstream	dimm	
CG5828	FBgn0010768	sqz	0	56	upstream		
fbl	FBgn0010768	sqz	0	7	upstream		
fbl	FBgn0010768	sqz	0	2	intron		
ppcs	FBgn0003499	sr	0	1	intron		
Ppcdc	FBgn0003499	sr	0	6	upstream		
ppcs	FBgn0003507	srp	0	1	upstream		
Ppcdc	FBgn0003507	srp	0	2	upstream		
Ppat-Dpck	FBgn0003507	srp	0	1	upstream		
Dpck	FBgn0003507	srp	0	1	upstream		
fbl	FBgn0003507	srp	0	1	upstream		
fbl	FBgn0003507	srp	0	1	intron		
ppcs	FBgn0016917	STAT92E	0	2	intron	toy, ttk	slbo (pubmed)
ppcs	FBgn0033782	sug	0	1	upstream		
Dpck	FBgn0033782	sug	0	1	upstream		
CG5828	FBgn0033782	sug	0	1	upstream		
ppcs	FBgn0003651	svp	0	1	upstream		
Ppcdc	FBgn0003651	svp	0	1	upstream		
CG5828	FBgn0003651	svp	0	1	upstream		
ppcs	FBgn0041092	tai	0	1	upstream	usp, ab, EcR	
ppcs	FBgn0041092	tai	0	2	intron	usp, ab, EcR	
Ppcdc	FBgn0023076	tai	0	1	upstream	cyc	
Ppcdc	FBgn0041092	tai	0	1	upstream	usp, ab	

Ppcdc	FBgn0015550	tap	0	2	upstream		
ppcs	FBgn0264075	tgo	1	0	intron		
ppcs	FBgn0004666	tgo	0	4	upstream	tgo	tgo (pubmed)
ppcs	FBgn0015014	tgo	0	10	upstream		
ppcs	FBgn0015542	tgo	0	4	upstream		
ppcs	FBgn0262139	tgo	0	2	upstream	tgo	tgo (pubmed)
Ppat-Dpck	FBgn0264075	tgo	1	0	upstream		
Ppat-Dpck	FBgn0003513	tgo	0	1	upstream	tgo	Dll, tgo (pubmed)
Ppat-Dpck	FBgn0015014	tgo	0	3	upstream		
Dpck	FBgn0004666	tgo	0	2	upstream	D, tgo	tgo (pubmed)
Dpck	FBgn0015014	tgo	0	1	upstream		
fbl	FBgn0015014	tgo	0	1	upstream		
ppcs	FBgn0004110	tin	0	1	intron	pnr	
Ppcdc	FBgn0004110	tin	0	1	upstream		
Ppcdc	FBgn0000964	tj	0	2	upstream		
CG5828	FBgn0000964	tj	0	1	upstream		
Dpck	FBgn0003720	tll	0	1	upstream		
fbl	FBgn0003720	tll	0	1	upstream		
ppcs	FBgn0019650	toy	0	2	intron	Stat92E	ey (pubmed)
Ppcdc	FBgn0019650	toy	0	2	upstream		ey (pubmed)
Ppat-Dpck	FBgn0013263	Trl	1	0	upstream		
CG5828	FBgn0013263	Trl	1	0	upstream	CG12155, ken	
ppcs	FBgn0003870	ttk	0	1	intron	gt, Stat92E, rib	run, aop (pubmed)
fbl	FBgn0003870	ttk	0	3	upstream	gt, D, rib	ac, run (pubmed)
ppcs	FBgn0003896	tup	0	1	upstream	pnr	
Ppcdc	FBgn0003896	tup	0	2	upstream		
CG5828	FBgn0003896	tup	0	2	upstream	pnr	sc (pubmed)

ppcs	FBgn0029711	Usf	0	1	intron		
ppcs	FBgn0003964	usp	0	1	upstream	Met, tai, EcR	
Ppcdc	FBgn0003964	usp	0	1	intron	tai	
Ppat-Dpck	FBgn0003964	usp	0	1	upstream	EcR	
ppcs	FBgn0003986	Vnd	0	2	intron		
Ppcdc	FBgn0003986	Vnd	0	1	intron		
fbl	FBgn0003986	Vnd	0	1	upstream		
ppcs	FBgn0016076	vri	0	2	upstream	kay	
fbl	FBgn0016076	vri	0	2	upstream	kay	
ppcs	FBgn0021872	Xbp1	0	1	upstream		
ppcs	FBgn0261113	Xrp1	0	1	upstream		
Ppat-Dpck	FBgn0036126	Xrp1	0	1	upstream	Xrp1, crc	
Ppat-Dpck	FBgn0261113	Xrp1	0	1	upstream		
fbl	FBgn0036126	Xrp1	0	4	upstream	Xrp1	
fbl	FBgn0261113	Xrp1	0	4	upstream		
ppcs	FBgn0004050	z	0	1	intron		
Ppat-Dpck	FBgn0004050	z	1	0	upstream		
fbl	FBgn0004050	z	0	1	upstream		
Ppcdc	FBgn0004053	zen	0	1	upstream		
Ppat-Dpck	FBgn0004606	zfh1	0	1	upstream		
CG5828	FBgn0004606	zfh1	1	0	upstream		
fbl	FBgn0004606	zfh1	0	1	upstream		
fbl	FBgn0004606	zfh1	0	1	intron		
ppcs	FBgn0259789	zld	2	1	intron		
ppcs	FBgn0259789	zld	1	1	upstream		
Ppat-Dpck	FBgn0259789	zld	1	0	upstream		
Dpck	FBgn0259789	zld	1	0	upstream		

CG5828	FBgn0259789	zld	1	0	upstream		
fbl	FBgn0259789	zld	1	0	upstream		

**Supplementary table3. Genotypes used in this study**	
**Figure**	
1, 2c, d, h, S4b	*w1118*
2e, f, g, I, j, k; 3a, c, d; S1e, f, g, j; S2a, b c, d, f, g	*CG31272 (MT)-GAl4, Tub-GAL80TS > +*
	CG31272-GAl4, Tub-GAL80TS > CG5828 (dPANK4)-RNAi
S1b	*esg-GAL4, tub-GAL80TS > +*
	esg-GAL4, tub-GAL80TS > Fbl-RNAi
S1h, i	*CG31272-GAl4, Tub-GAL80TS > +*
	CG31272-GAl4, Tub-GAL80TS > CG5828
3e	*esg-GAL4, tub-GAL80TS > +*
	esg-GAL4, tub-GAL80TS > Hmgcr-RNAi
	esg-GAL4, tub-GAL80TS > Qm-RNAi
	esg-GAL4, tub-GAL80TS > beta GGT-I-RNAi
3f	*CG31272-GAl4, Tub-GAL80TS > +*
	CG31272-GAl4, Tub-GAL80TS > Drip-RNAi
S2e	*esg-GAL4, tub-GAL80TS > +*
	esg-GAL4, tub-GAL80TS > Mof-RNAi
	esg-GAL4, tub-GAL80TS > Hat1-RNAi
	esg-GAL4, tub-GAL80TS > Gcn5-RNAi
S2i	*tsh-GAl4, Tub-GAL80TS > +*
	tsh-GAl4, Tub-GAL80TS > Drip-RNAi
4a, 5f	*esg-GAL4, tub-GAL80TS > UAS-GFP*
	esg-GAL4, tub-GAL80TS > UAS-GFP, UAS-yki3SA
4b	*esg- LexA, tub-GAL80TS > +; CG31272>+*
	esg- LexA, tub-GAL80TS > LexAop-yki3SA-GFP 2nd; CG31272>+
	esg- LexA, tub-GAL80TS > LexAop-yki3SA-GFP 2nd; CG31272>Smvt-RNAi
4c, d, e, f, g, I, j, k, l, m; S3b, c, d, e, m, l	*esg- LexA, tub-GAL80TS > +; CG31272>+*
	esg- LexA, tub-GAL80TS > LexAop-yki3SA-GFP 2nd; CG31272>+
	esg- LexA, tub-GAL80TS > LexAop-yki3SA-GFP 2nd; CG31272>Fbl-RNAi
	esg- LexA, tub-GAL80TS > LexAop-yki3SA-GFP 2nd; CG31272>CG5828
S3f, g, h, I, j, k	*esg- LexA, tub-GAL80TS > +; CG31272>+*
	esg- LexA, tub-GAL80TS > LexAop-yki3SA-GFP 3rd; CG31272>+

	esg- LexA, tub-GAL80TS > LexAop-yki3SA-GFP 3rd; CG31272>Fbl-RNAi
	esg- LexA, tub-GAL80TS > LexAop-yki3SA-GFP 3rd; CG31272>CG5828
	CG31272-GAl4, Tub-GAL80TS > +
5a	*CG31272-GAl4, Tub-GAL80TS > Myc*
5c	*CG31272-GAl4, Tub-GAL80TS > Myc-HA*
	CG31272-GAl4, Tub-GAL80TS > +
S4c, d	*CG31272-GAl4, Tub-GAL80TS > Myc-RNAi*
	esg- LexA, tub-GAL80TS > +; CG31272>+
	esg- LexA, tub-GAL80TS > LexAop-yki3SA-GFP 2nd; CG31272>+
5g, h, I, j, k, l, m n , S4e, f, g, h	*esg- LexA, tub-GAL80TS > LexAop-yki3SA-GFP 2nd; CG31272>Myc-RNAi*
	esg- LexA, tub-GAL80TS > +; CG31272>+
	esg- LexA, tub-GAL80TS > LexAop-yki3SA-GFP 3rd; CG31272>+
S4i, j, k, l, m, n	*esg- LexA, tub-GAL80TS > LexAop-yki3SA-GFP 3rd; CG31272>Myc-RNAi*
	CG31272-GAl4, Tub-GAL80TS > +
5o	*CG31272-GAl4, Tub-GAL80TS > PvR[Act]*
	esg- LexA, tub-GAL80TS > +; CG31272>+
	esg- LexA, tub-GAL80TS > LexAop-yki3SA-GFP 2nd; CG31272>+
5p	*esg- LexA, tub-GAL80TS > LexAop-yki3SA-GFP 2nd; CG31272>Pvr-RNAi*

Supplementary table4. Primers used in this study		
		**RT-qPCR primers**
dPANK4 (CG5828)	Forward	TTACAGATCCCTGGCTGAGAC
	Reverse	CCACCTTGTGTCCTCATCCG
Fbl	Forward	TTCTCTTCGCCGATCTGCATA
	Reverse	GAACTGCTGCTTTTCGCTTTTTA
FASN1	Forward	GACATGGTCAACGATGATCCC
	Reverse	ACCGAAGAACTGTTGGTCAAAG
ACC	Forward	ACAAGATGAAGAACCATGCCAT
	Reverse	TTCGCGGGACTTCTGTTGC
AcCoAS	Forward	CCATGATTCTGGAGCTGCCTA
	Reverse	GCCTTCAGGTACAGGGGTTTC
Atpcl	Forward	TTTCCACAGTAAATTCCACGACA
	Reverse	GGCGCTTGATAAGTTGATCGG
Gcn5	Forward	GGTGGAAACAAGAGGACCAGTG
	Reverse	CCAAATTCTCACTGCTTGGA
Elp3	Forward	AATTCTGCTTCCAAAGCTGAGG
	Reverse	GCCGGGACAATAGACGCATA
Hat1	Forward	TGGTAGACTTTAAGCTGATCCGT
	Reverse	CTCCCCGAAAATCTGGTGGG
Mof	Forward	GAGCCAACCGATGCGTACA
	Reverse	TCCTCCGAAATGGGACTGATG
Nej	Forward	ATGATGGCCGATCACTTAGACG
	Reverse	GATTTGTGGTTACACCGGAGG
Fpps	Forward	GCAACGCCTGATCTCTACCAG
	Reverse	TTGGAGCGTCGATAAGGTTCT
Hmgcr	Forward	GCTGCACTGCCGTACTGTA
	Reverse	AATGCCCAGCACATATTTGGA
Qm	Forward	TAAATGCGGCCAACTATGCAC
	Reverse	CATCAGCTTGTAATCCGACTCG
beta GGT-I	Forward	ATGGCCTCGCACGATAACAC
	Reverse	GCAATGAGTTTAGCACATCCAGG
CG13200	Forward	GCATATGCGACAAAGTGGGCC
	Reverse	AACATTCACCGCAAGGGCTCC
RP49	Forward	AAGAAGCGCACCAAGCACTTCATC
	Reverse	TCTGTTGTCGATACCCTTGGGCTT
		**ChIP-qPCR primers**
dPANK4 (CG5828)	Forward	CCAGCTGAGGTGTGCTGG
	Reverse	TTGAACAGTCTTATTGCAACTATCG

Fbl	Forward	GGTGCACATAAAATGTGTGGGA
	Reverse	ATCGAAAAGCGCAGTGTTGG
Tll (Neg)	Forward	CCTTCTTGAATTTCCAGGTCGC
	Reverse	CGTCTTGTCCACCACACAGA
		**CG5828 into TOPO clone**
dPANK4 (CG5828)	Forward	caccATGTACAGTAGCAGCTTGCTGCCG
	Reverse	CTAGCTGGGCGCAGCCGGTTCGAA
		**CG5828 into pGEX-4T2 InFusion**
dPANK4 (CG5828)	Forward	TCCCCAGGAATTCCCATGTACAGTAGCAGCTTGCTGCC
	Reverse	CGCTCGAGTCGACCCCTAGCTGGGCGCAGCCGG
dPANK4 (CG5828)-D209A	Forward	GGTCTTTGTGGCGAACAGCGGCG
	Reverse	ACGGCGCACTTGTGT
dPANK4 (CG5828)-D245A	Forward	TGCTCTAAATGCGGTGACCAGCG
	Reverse	GGTTCACTGTTGGCG
