# Supplementary material for: Renal coenzyme A (CoA) production from VB5 fuels stem cell proliferation and tumor growth
Source: Nat Commun. 2026 Apr 18;17:5383. doi: 10.1038/s41467-026-71716-1 (PMC13276073; doi:10.1038/s41467-026-71716-1)
Supplement: Supplementary file 2 — Description of Additional Supplementary Files [file 41467_2026_71716_MOESM2_ESM.pdf]

Description of Additional Supplementary Files

File Name: Supplementary Data 1
Description: **Targeted metabolomics data from whole-body control and MT-specific *dPANK4* knockdown flies.**
Processed targeted LC–MS/MS metabolomics data from whole adult flies are provided as integrated peak-area values for individual metabolites across biological replicates. Columns Ctrl_1–Ctrl_5 correspond to control samples, and dPANK4KD_1–dPANK4KD_5 correspond to MT-specific dPank4 knockdown samples. Values are reported as integrated total ion current (TIC) peak areas. Missing or undetected values are indicated as N/A.

File Name: Supplementary Data 2
Description: **Targeted metabolomics data from guts of control and MT-specific *dPANK4* knockdown flies.**
Processed targeted LC–MS/MS metabolomics data from dissected adult guts are provided as integrated peak-area values for individual metabolites across biological replicates. Columns Ctrl_1–Ctrl_4 correspond to control gut samples, and dPANK4KD_1–dPANK4KD_4 correspond to MT-specific dPank4 knockdown gut samples. Values are reported as integrated total ion current (TIC) peak areas. Missing or undetected values are indicated as N/A.

File Name: Supplementary Data 3
Description: **Targeted metabolomics data from guts of control and Yki tumor flies.**
Processed targeted LC–MS/MS metabolomics data from dissected adult guts are provided as integrated peak-area values for individual metabolites across biological replicates. Columns Ctrl_1–Ctrl_4 correspond to control gut samples, and Yki_1–Yki_4 correspond to Yki gut samples. Values are reported as integrated total ion current (TIC) peak areas. Missing or undetected values are indicated as N/A.
