# Supplementary material for: Renal coenzyme A (CoA) production from VB5 fuels stem cell proliferation and tumor growth
Source: Nat Commun. 2026 Apr 18;17:5383. doi: 10.1038/s41467-026-71716-1 (PMC13276073; doi:10.1038/s41467-026-71716-1)
Supplement: Supplementary file 7 — Transparent Peer Review file [file 41467_2026_71716_MOESM7_ESM.pdf]

nature portfolio

Peer Review File

# Renal Coenzyme A (CoA) Production from VB5 Fuels Stem Cell Proliferation and Tumor Growth

Corresponding Author: Professor Norbert Perrimon

**This file contains all reviewer reports in order by version, followed by all author rebuttals in order by version.**

Version 0:

Reviewer comments:

Reviewer #1

(Remarks to the Author)
This article by Miao et al. presents compelling evidence for a fascinating role of kidney-derived Coenzyme A in regulating intestinal stem cell proliferation and tumourigenesis through inter-organ metabolic communication. Briefly, dietary supplementation of Vitamin B5 promotes CoA synthesis specifically in the Malpighian tubules (MTs) through upregulating Fbl and downregulating dPANK4, which in turn activates ISC proliferation. This MT CoA biosynthesis-driven ISC proliferation can be induced by physiological cues, such as mating, or by oncogenic transformation, notably via Yorkie hyperactivation. Mechanistically, CoA produced in MTs enhances the activity of both the MVA and isoprenoid backbone biosynthesis pathway in the gut to increase ISC proliferation. In addition, the authors identified Fbl and dPANK4 as direct transcriptional targets of Myc, which is highly upregulated in MTs in response to mating, or tumour-derived PDGF/VEGF signalling. Finally, the authors shown evidence suggesting a potentially conserved role of the Myc-CoA-isoprenoid axis in human papillary renal cell carcinoma. These suggest the potential therapeutic relevance of targeting this metabolic pathway in renal cancers.

Overall, this study provides a comprehensive framework for understanding how renal-intestinal metabolic coordination governs ISC proliferation. However, there are critical aspects of the study that require further clarification and/or solidification through experimental work. Beyond those, the authors need to provide more detailed description of experimental protocols, genetic drivers used and data analysis methods. All figure numbers, genotype labels, legends, and other related annotations should be carefully reviewed and corrected, as multiple inconsistencies and errors were spotted throughout the manuscript and figure panels.

Major comments:

Q1: related to the following statement by authors: “Interestingly, we observed a modest increase in pH3+ signal with 1 mM VB5 supplementation and a more pronounced effect at 2.5 mM (Fig. 1e), indicating a linear and dose-dependent response to dietary VB5 supplementation.”
What is the maximum concentration of VB5 for promoting ISC proliferation? With only two doses, it is not sufficient to conclude that it has a linear and dose-dependent response.

Q2: related to the following statement by authors: “Notably, VB5 supplementation increased the number of ISCs and EBs, suggesting an elevated proportion of EBs resulted from ISC division.”
The statement here is confusing --- the increased number of ISCs and EBs could not suggest an elevated proportion of EBs (although your quantification did show this). Moreover, the shape of EBs seems to be quite different from what has been published previously. What is the region that is being imaged and quantified? Do you see the increase in ISCs and EBs throughout the whole midgut, or is it limited to R4/R5? What happens to EE cells upon VB5 supplementation?

Q3: The possibility of the gut directly absorbing VB5 and synthesizing CoA has not been properly addressed, and it is a key point: Have the authors tried to knockdown Fbl specifically in ECs and check its effect on VB5-induced ISC proliferation? Is it possible that interfering VB5 absorption from ECs can have a non-cell-autonomous effect on ISC proliferation? In Extended Data Fig. 1b, knockdown of Fbl in ISCs and EBs surprisingly promoted ISC proliferation upon VB5 feeding, what is the possible explanation of this? Would it cause a similar increase in homeostatic ISC proliferation (without VB5 supplementation) since it is not shown in the figure?

Q4: Have you checked the changes in the protein levels of Fbl or dPANK4 upon VB5 supplementation?

Q5: What is the MT-gal4 driver? What cells are being targeted? Unless I am missing something, I cannot see this very important information in the description of the experimental work and results. Related to this, have you checked the effects of knocking down Fbl in MTs on VB5-induced ISC proliferation? In addition, have you tried to check whether overexpressing Fbl in MTs is also sufficient to increase ISC proliferation?

Q6: related to the following statement by authors: "These mitotic signals first appeared in the posterior R4-R5 region, which lies adjacent to the MT-gut junction, and progressively spread anteriorly upon prolonged dPANK4 knockdown (Fig. 1a and Extended Data Fig. 2g)."
The images shown here (Extended Data Fig. 2g) are not clear enough to prove that the pro-mitosis signal is "progressively spreading anteriorly". There should be at least quantifications of pH3+ cells for both the anterior and posterior midguts at different time points following dPANK4 KD induction to demonstrate that pro-proliferative signal originates locally at the R5-MT junction rather than being disseminated through circulation.

Q7: According to Cabrero et al. (Reference 45), Drip protein is only highly expressed in stellate cells. In addition, the snRNA-seq data (Reference 35) also showed that Drip expression is very minimal in principal cells except for lower ureter PCs. I don't know why the expression data you showed in Extended Data Figure 2h seems to be very different from the published one, although you used the same dataset. Could you please explain why knocking down Drip in principal cells, where it is not highly expressed, could have such a strong effect on inhibiting VB5-induced proliferation, whilst the knockdown in stellate cells almost had no effects?

Q8: How would knocking down Drip in principal cells affect homeostatic ISC proliferation (without VB5 feeding)? Is it possible that Drip is required for its role other than transporting metabolites? Apart from Drip, any other aquaporins might be involved in this? Have you checked the levels of CoA-related metabolites in the guts when Drip is knocked down in MTs by VB5 isotope tracing?

Q9: related to the following statement by authors: "We observed significant transcriptional changes in CoA biosynthesis genes, Fbl and dPANK4, specifically in the MT principal cells, but not in the gut ISCs/tumors (Fig. 4a and Extended Data Fig. 3a), suggesting a potential contribution of MT-derived CoA synthesis to gut tumor growth."
Have you tried to validate the gene expression of Fbl and dPANK4 in principal cells and ISCs by doing qRT-PCR on sorted cells, or even dissected tissues?

Q10: related to the following statement by authors "Both interventions significantly suppressed tumor growth, as evidenced by reduced gut width, decreased Yki-GFP signal, and lower mitotic (pH3+) cell counts (Fig. 4c-f and Extended Data Fig. 3b-i)."
The corresponding figures are really confusing. I can see that Extended Figure 3 f-j validated the same thing using a different RNAi, but it seems that Extended data figure 3 b-e showed the same experiments again as Figure 4c-f?
Another question is why there are no significant changes in the gut width in extended figure 3c, while the difference is quite big in figure 4d and extended figure 3g?

Q11: Knocking down Fbl in MTs significantly extended the survival of yki tumour flies. Have you observed the same thing with MT-specific dPANK4 overexpression?

Q12: Have you tested whether CoQ/dolichol might also be a downstream pathway of MVA?

Q13: Is it possible to identify the metabolites transported between MTs and guts that mediate the role of MT CoA biosynthesis in ISC proliferation

Q14: Have you checked whether CoA biosynthesis is also playing a conserved role in human gastrointestinal cancer patients?

Q15: The physiological role and expression domain of Myc in the system requires further experimentation to substantiate the conclusions/working model. a) Where in the MT is Myc upregulated? This should be addressed using Myc antibody staining combined with markers of MT cell sub-types. It is highly surprising that Myc from the tubules is sufficient to decrease ISC proliferation in Yki tumours. I would expect Yki would upregulate Myc in the gut itself. Have the authors investigated this? This is very important information missing. B) I would be very cautious with the interpretation of the Myc overexpression results presented in Fig 5d,e. Because of the lack of information on the MT-gal4 driver (Q5) I can't tell where Myc is being overexpressed. Ectopic overexpression of Myc at high levels highly toxic to cells and can trigger apoptosis https://www.sciencedirect.com/science/article/pii/S153561080800370X. We have experienced the same in flies (non-published work by Reviewer). As such, close analysis of the impact of this manipulation to MT physiology and organismal function is essential. For example, the hyperproliferation of the gut upon MT overexpression of Myc can be secondary to damage in the tubule leading to the production of stress signals that remotely activate gut hyperproliferation. Furthermore, in all experiments involving loss of function of Myc in the paper, there is no analysis of the effect of that alone in the tubules, whole animal and gut. In other words, MT>MycRNAi only controls need to be included in the study.

Materials and Methods:

In general, the number of flies used for pH3 or GFP quantification is too low.

For VB5 isotope tracing experiments, have you checked the labelling efficiency at 48h?
There is a lack of detailed description on quantification/analysis methods in general. For example, how did you measure the gut width in the R4/R5 region? Did you perform a single measurement at a randomly selected section within this region, or did you take three to five measurements across the R4/R5 and calculate the mean value? In addition, how did you exactly measure the number of GFP+ cells in the gut? Because it does not make sense to quantify "by measuring the average GFP signaling strength from individual flies". Please clearly state the approach used, including how ROIs were selected and whether averaging was applied to ensure accuracy and reproducibility.

For confocal imaging, there is no description on imaging parameters. Excitation/emission wavelengths? Z-stacks or single plane? If you also did tile scan, what is the relative parameters?

For lifespan experiment, which day is considered to be Day 0? Did you start to count the death of flies immediately after you transfer flies to 29 °C, or you started to count a few days after transgene induction? How often did you flip the flies onto fresh food and how many flies were in each vial?

Please also note my comments on genetic drivers used in some of the points above.

Minor comments:

Figure 1 legend- Line 17, 1) it is not the GFP intensity that should be quantified here; 2) Figure 1 f' VB5 condition has 7 dots on the plot but the n stated here is 4-6. Please check the n number carefully.

Figure 2i and Extended Data Figure 1i, the purple group should be MTts>+, mated

Figure 2g, the genotype should be MTts>dPANK4-RNAi.
"Consistently, VB5 levels were significantly reduced in dPANK4 knockdown flies (Extended Data Fig. 1i), suggesting altered CoA synthesis."
Figure number here should be Extended Data Fig. 1j.
"Moreover, there were also elevated labelled fractions of CoA derivatives, including acetyl-CoA, malonyl-CoA, and HMG-CoA (Fig. 2k)."
The sentence here is redundant. Same information has been described just before it.

"Additionally, MYC expression showed a strong inverse correlation with PANK4 expression in pRCC patients (Extended Data Fig. 5d)…"
Should be Extended figure 5b.

"Notably, these trends were not observed in ccRCC, where PANK4 and PPAT expression did not correlate with survival (Extended Data Fig. 5d-e), and higher PANK3 expression was unexpectedly associated with better outcomes (Extended Data Fig. 5f)."
Figure numbers did not match the results described here.

Reviewer #2

(Remarks to the Author)
This manuscript investigates how a renal–intestinal metabolic axis, mediated by vitamin B5/CoA supply from the Malpighian tubules (MT), regulates intestinal stem cell (ISC) proliferation and tumor growth in Drosophila. The authors show that Myc upregulates CoA biosynthesis in MTs by modulating Fbl and dPANK4, thereby enhancing CoA production. This systemic increase in CoA availability drives mevalonate (MVA) pathway activity in the intestine, promotes isoprenoid biosynthesis, and subsequently enhances ISC proliferation and Yki-driven tumorigenesis. Human data analyses further link MYC and CoA–isoprenoid pathway gene expression to poor prognosis in papillary renal cell carcinoma (pRCC).

The study is conceptually novel, providing a mechanistic model for multi-organ coordination of metabolite regulation to control stem cell function and tumorigenesis, and will thus be of significance to multiple scientific fields. The integration of tissue-specific genetics, metabolomics, isotope tracing, pharmacology, and cross-species comparative analyses is a major strength and highlights strong potential impact of the data provided in the manuscript. However, there are just a few concerns (experimentally and conceptually) in the current version of this intriguing study.

Primary Concerns:

1. pH3 staining is used as the primary readout for ISC proliferation, but the mechanistic link between MT-derived CoA and ISC division remains unclear. What are the direct downstream effectors of the isoprenoid pathway in this context that impact ISC proliferation? Is this a specific regulation (targeting distinct growth / proliferation / differentiation pathways) or more general? Demonstrating effects on known prenylated signaling proteins (such as Ras, Rho, Rab, etc.) would strengthen the mechanistic conclusions.

2. In the presence of intestinal tumorigenesis, Myc is upregulated in MTs in response to Pvf1–Pvr signaling, reprogramming specific aspects of host metabolism to support tumor growth. The authors also propose that CoA biosynthesis in MTs is

activated after mating to support gut homeostasis and renewal (non-tumor conditions). However, dPANK4 appears to exert opposite effects on intestinal cell proliferation before and after mating. This discrepancy requires explanation. Additionally, to improve clarity, “MTts>, mated” should be explicitly included in Fig. 2.

3. MT-specific knockdown of the aquaporin Drip suppresses tumor-dependent phenotypes, but this is unlikely to represent direct transport of CoA intermediates and more likely reflects general solute transfer and altered fluid dynamics.

Other Concerns:

Most metabolomic analyses are performed on whole bodies (Fig. 2), which obscures the link between MTs and intestine, and there is no quantification of metabolite B5/CoA levels in intestinal samples after MT-specific attenuation of CoA biosynthesis. Experimentally, it’s unclear why certain metabolomic/metabolite assays were not performed in whole tissues.

Throughout the manuscript, “EGTGal4” and “GutGal4” are used inconsistently. Please revise for clarity.

Page 6, lines 12–13: “Extended Data Fig. 1I” should be corrected to “Extended Data Fig. 1J.”

Figure 3J and Page 9, line 32: “including Hmgcr, Fpps, Qm, and β-GGT-I” is misleading, as Hmgcr is not significantly upregulated.

The tissue source used in each experiment, especially metabolomic or metabolite measurement, needs to be clearly stated in figures.

Reviewer #3

(Remarks to the Author)
Dear Editor,

I have now thoroughly read the study by Miao et al. titled ‘Renal Coenzyme A (CoA) Production Fuels Stem Cell Proliferation and Tumor Growth’. In this study, the authors uncover a novel axis in which dietary vitamin B5 induces CoA biosynthesis in the Malpighian tubules (MT) of D. melanogaster. In a feedback loop driven by, probably, diffusible metabolites, this leads to growth of intestinal stem cells in the fly gut. Importantly, the increase in CoA production in MTs is induced by the transcription factor Myc. It induces the mevalonate–isoprenoid pathway in the gut, which in turn is important for cell growth. Further, the authors show that this axis, including the involvement of Myc, is important in a subset of human renal cancers and that the mevalonate–isoprenoid pathway is important for the growth of these cancers.

Overall, the authors uncover several very novel pathways; the study is thus impactful and original. It adds significantly to our understanding in terms of cofactor-driven local and inter-organ cellular programmes. There are a few points that need some further consideration. Up front, I would like to note that not all of them need addressing necessarily to meet the impact for this journal, but in some cases, the wording might need to be somewhat adjusted.

The main point is that the axis between the gut and the MT is not as clear as portrayed. In particular, Fig. 5p is not fully backed by data. The authors depict an axis of tumour-derived Pvf1, which activates the Pvr in the MT. This is plausible, but as far as I can tell, the authors never show it directly. Instead, they rely on published data proving Pvf1 can induce Myc in the MT, as well as the utilisation of a constitutively active Pvr in the MT. However, the correct way of showing this would be to have tumours with a knockdown of Pvf1, which should reduce Myc activity and CoA biosynthesis, as well as the mevalonate–isoprenoid pathway. The authors could thus either prove formally that the gut–Pvf1→MT–Pvr axis exists or tone down the wording accordingly.

In the same vein, the notion that it is MT-derived metabolites driving ISC and/or tumour growth in the gut is very plausible but not proven. MT-restricted knockdown of Drip is indeed a good indicator of fluid transfer between the sites, but that can also be true for signalling molecules rather than metabolites. I acknowledge the difficulty in identifying the precise molecules that transmit this signal, so a clear and transparent assessment of the data is, in my opinion, sufficient. Alternatively, as the mevalonate pathway seems to play such a crucial role in this process, intermediates of this pathway could be fed to the animals to test if their presence is sufficient to mediate the same effect. To summarise the two points above, my feeling is that the MT–gut axis clearly exists, but that the manuscript should be very clear about what conclusions can unambiguously be drawn from the current data.

The data on human tumours presented by the authors are strong, interesting, and harbour therapeutic potential. However, it does not seem to have much to do with the inter-organ axis seen in the fly. Instead, this seems to be a cell-autonomous or at least organ-autonomous effect in the kidneys. In reading the manuscript, the findings from the fly and the human sometimes seem to be a little conflated. I would suggest the authors carefully read the manuscript to point out not only the similarities but also the clear differences between the two systems.

The fly Pank4 identified by the authors is likely indeed to have analogous activity. However, the authors never directly show the phosphatase activity. In vitro assays with recombinant proteins are often tricky to establish; however, the authors could use the metabolomics dataset presented under Fig. 2j to verify the levels of the dPANK4 product pantetheine. If their assumption is correct, pantetheine levels should drop in MTTS>dPANK4RNAi flies.

Referring to Fig. 2k, there seems to be some confusion with the terminology, and possibly the data. The data displayed seem to be a fold change over the control. Accordingly, most grey control bars hover around the 1.0 mark. For CoA, however, it rises to 1.1, with all data points well above 1.0. If the control is normalised to 1, this should not be possible. Please check this data. Also, the authors refer to these values as fractional enrichment. Usually, when performing isotope tracing, the fractional enrichment refers to values of labelled over unlabelled forms of any given metabolite. Here I cannot see the non-labelled values. Or are these fractions that are then further normalised to the control, which would be a rather unusual thing to do? Please clarify this point.

Minor points:

- The H3 signal is quite noisy. An overlay with arrows pointing at those cells counted for quantification would be of great help.
- Does simvastatin also increase survival?
- Transcriptional levels of Myc as a readout for its activity are often difficult due to its short half-life. Using Myc signatures often yields better results. The authors might consider this.
- In panels such as 2j, the p-value is displayed in two ways, position in the graph and colour. Personally, I do not think the same information should be displayed twice, but this is ultimately an editorial decision.

I hope the comments can be of help to the authors.

Reviewer #4

(Remarks to the Author)
I co-reviewed this manuscript with one of the reviewers who provided the listed reports. This is part of the Nature Communications initiative to facilitate training in peer review and to provide appropriate recognition for Early Career Researchers who co-review manuscripts.

Version 1:

Reviewer comments:

Reviewer #1

(Remarks to the Author)
The authors have comprehensively address my major comments, including new experimental data. I have no further concerns.

Reviewer #2

(Remarks to the Author)
In this revised version of their manuscript, the authors have addressed almost all of my concerns from the previous submission (experimentally and conceptually). Any major concerns that would prohibit publication have been corrected.

The study is conceptually and mechanistically novel, providing a putatively conserved model for multi-organ coordination of metabolite regulation to control stem cell function, tissue regeneration, and perhaps even tumorigenesis. The methodology is rigorous, and these data are likely to be of significance to multiple scientific fields.

Reviewer #3

(Remarks to the Author)
Dear Editor,
I have now reviewed the latest version of the manuscript ‘Renal Coenzyme A (CoA) Production from VB5 Fuels Stem Cell Proliferation and Tumor Growt’ by Miao et al.
The authors have taken my comments very seriously and addressed them very well. In particular, proving that the putative dPANK4 (CG5828) is indeed a fly-orthologue of mammalian PANK4 is of great value and to a certain extend goes even beyond what I asked for. I also feel that the existence of the MT–gut axis has become clearer thanks to the new data, and that the authors more clearly state that while such an axis might exist in humans, it still needs further evaluation.
I have also looked over the points raised by my fellow reviewers and conclude that those concerns pointed out by them, and shared by me, have also been addressed adequately.
Overall, I am thus happy for this manuscript to be accepted for publication.

**Open Access** This Peer Review File is licensed under a Creative Commons Attribution 4.0 International License, which permits use, sharing, adaptation, distribution and reproduction in any medium or format, as long as you give appropriate credit to the original author(s) and the source, provide a link to the Creative Commons license, and indicate if changes were made.
In cases where reviewers are anonymous, credit should be given to 'Anonymous Referee' and the source.
The images or other third party material in this Peer Review File are included in the article's Creative Commons license, unless indicated otherwise in a credit line to the material. If material is not included in the article's Creative Commons license and your intended use is not permitted by statutory regulation or exceeds the permitted use, you will need to obtain permission directly from the copyright holder.
To view a copy of this license, visit https://creativecommons.org/licenses/by/4.0/

Reviewer #1 (Remarks to the Author):

This article by Miao et al. presents compelling evidence for a fascinating role of kidney-derived Coenzyme A in regulating intestinal stem cell proliferation and tumourigenesis through inter-organ metabolic communication. Briefly, dietary supplementation of Vitamin B5 promotes CoA synthesis specifically in the Malpighian tubules (MTs) through upregulating Fbl and downregulating dPANK4, which in turn activates ISC proliferation. This MT CoA biosynthesis-driven ISC proliferation can be induced by physiological cues, such as mating, or by oncogenic transformation, notably via Yorkie hyperactivation. Mechanistically, CoA produced in MTs enhances the activity of both the MVA and isoprenoid backbone biosynthesis pathway in the gut to increase ISC proliferation. In addition, the authors identified Fbl and dPANK4 as direct transcriptional targets of Myc, which is highly upregulated in MTs in response to mating, or tumour-derived PDGF/VEGF signalling. Finally, the authors shown evidence suggesting a potentially conserved role of the Myc-CoA-isoprenoid axis in human papillary renal cell carcinoma. These suggest the potential therapeutic relevance of targeting this metabolic pathway in renal cancers.

Overall, this study provides a comprehensive framework for understanding how renal-intestinal metabolic coordination governs ISC proliferation. However, there are critical aspects of the study that require further clarification and/or solidification through experimental work. Beyond those, the authors need to provide more detailed description of experimental protocols, genetic drivers used and data analysis methods. All figure numbers, genotype labels, legends, and other related annotations should be carefully reviewed and corrected, as multiple inconsistencies and errors were spotted throughout the manuscript and figure panels.

Thank you very much for your insightful comments, which have guided us to substantially improve the quality of our manuscript. We sincerely appreciate your time and effort. Point-by-point responses are provided below.

Major comments:

Q1: related to the following statement by authors: “Interestingly, we observed a modest increase in pH3+ signal with 1 mM VB5 supplementation and a more pronounced effect at 2.5 mM (Fig. 1e), indicating a linear and dose-dependent response to dietary VB5 supplementation.”
What is the maximum concentration of VB5 for promoting ISC proliferation? With only two doses, it is not sufficient to conclude that it has a linear and dose-dependent response.

Thank you for the insightful comment. We did test higher concentration 5mM, which did not promote ISC proliferation. Since the standard cornmeal food contains some VB5, we further validated our findings using a *Drosophila* holidic diet [1-3] with VB5 depleted and

supplemented with VB5 at 0, 1, 2.5, 5, or 10 mM. In these settings, we observed the strongest induction of $pH3^+$ signal at 2.5 mM VB5, with a statistically significant but smaller increase at 5 mM. Notably, 1 mM VB5 did not induce the $pH3^+$ signal in the defined food, likely due to the absence of background VB5 present in cornmeal-based standard food. For the same reason, the induction of $pH3^+$ signal was diminished at the higher dose of 10 mM VB5. Together, these results reflect a dose-dependent response upon VB5 supplementation with the most significant induction of ISC proliferation at 2.5mM VB5 on both standard cornmeal food and holidic diet. We appreciate the reviewer's comment, which prompted clarification of the VB5 dose-response. We have updated **Fig. 1e**, included new data in **Extended Data Fig.1a**, and revised the corresponding text in the manuscript at **p. 5, lines 17-31**:

*"Interestingly, we observed a modest increase in pH3+ signal with 1 mM VB5 supplementation and a more pronounced effect at 2.5 mM, whereas no induction was observed at the higher concentration at 5 mM (Fig. 1e). Because the cornmeal-based fly diet naturally contains VB5, we next examined the dose-dependence using a Drosophila holidic medium [35-37] depleted of VB5 and supplemented with 0, 1, 2.5, 5, or 10 mM VB5 (Extended Data Fig. 1a). Under these conditions, the strongest induction of $pH3^+$ signal was observed at 2.5 mM VB5 supplementation, with a statistically significant but smaller increase at 5 mM VB5 supplementation. Notably, 1 mM VB5 did not induce the $pH3^+$ signal in the defined food, likely due to the absence of baseline VB5 present in cornmeal-based standard food. Consistently, the induction of $pH3^+$ signal was diminished at the higher dose of 10 mM VB5. Together, these results indicate a dose-dependent effect of dietary VB5 supplementation on cell proliferation. Accordingly, we used 2.5 mM VB5 supplementation for subsequent experiments."*

Q2: related to the following statement by authors: "Notably, VB5 supplementation increased the number of ISCs and EBs, suggesting an elevated proportion of EBs resulted from ISC division."
The statement here is confusing --- the increased number of ISCs and EBs could not suggest an elevated proportion of EBs (although your quantification did show this). Moreover, the shape of EBs seems to be quite different from what has been published previously. What is the region that is being imaged and quantified? Do you see the increase in ISCs and EBs throughout the whole midgut, or is it limited to R4/R5? What happens to EE cells upon VB5 supplementation?

Thank you for your comments. Here are our responses to your questions.

Q2-1: The statement here is confusing --- the increased number of ISCs and EBs could not suggest an elevated proportion of EBs (although your quantification did show this).

We have revised the text regarding the statement to avoid confusion **at p. 5, lines 37-38**:

*"Notably, VB5 supplementation increased the proportion of ISCs and EBs, suggesting that VB5 enhances stem cell proliferation or self-renewal, thus stimulates differentiation toward early progenitor stages."*

Q2-2: Moreover, the shape of EBs seems to be quite different from what has been published previously.

We note that most prior studies focusing on EB morphology or protein co-localization typically image at higher magnification (e.g., 40x–60x oil) to resolve subcellular features. By contrast, our primary goal here was EB quantification, so we imaged at 20x to capture larger fields and maximize the number of cells per frame. In addition, we acquired z-stacks across multiple optical planes and quantified from maximum-intensity projections; consequently, some EBs are not in the same focal plane and may appear "upright" or oblique, which can subtly alter apparent outline/shape after projection. In many published figures, by comparison, representative images often show a single optical slice chosen where cells are co-planar and well oriented, yielding more uniform morphology. Thus, the perceived differences most likely reflect magnification and z-stack/projection choices rather than true morphological divergence. We appreciate the reviewer for highlighting this point and have added explicit acquisition and analysis parameters accordingly (objective, pixel size, z-step, projection method, ROI policy) to the Methods **(p. 22, lines 19–36)**:

*"Confocal images were acquired on an Olympus IX83 system with FV3000 scan unit (FV31S-SW) using a UPLSAPO 20×/0.75 NA, dry objective. Acquisition parameters were kept constant across genotypes within each experiment. Images were collected as z-stacks with z-step (voxel depth = 2.50 µm); in-plane sampling was 0.795 × 0.795 µm/pixel (as recorded in Fiji/ImageJ). The excitation/detection windows were: DAPI: 405 nm excitation, detection 430–470 nm; GFP and BODIPY: 488 nm excitation, detection 500–547 nm; pH3 (Alexa 594): 561 nm excitation; detection 570–670 nm. All matched genotypes were imaged in the same session with identical laser power, detector gain/offset, pinhole, scan speed, and pixel size. Unless noted, guts were imaged in the R4-R5 region. For display and all quantifications, we used maximum-intensity projections spanning the epithelium. Scale bars were rendered from embedded metadata in Fiji. For each image, ROIs were placed using anatomical landmarks blinded to genotype/condition and applied identically across channels. Within the saved R4-R5 ROI, gut width was measured once per gut at a random region perpendicular to the gut axis using the Straight Line tool followed by Analyze→ Measure. pH3+ cells of the whole gut were counted manually; counting was performed blinded. Percentage of GFP+ cells was calculated as (number of GFP+ cells/ total cell) x 100, where total cells were defined by nuclear (DAPI) counts within an identical ROI using Fiji-imageJ."*

Q2-3: What is the region that is being imaged and quantified?

For all pH3 staining experiments (vitamin B5 supplementation, *dPank4* knockdown, Yki activation, etc.), pH3[+] cells were quantified across the entire gut. In this VB5-supplementation condition, the proliferative increase was most pronounced in the R4–R5 region; therefore, cell-type composition (ISC/EB/EC) and GFP-based percentages were analyzed specifically at R4–R5 to resolve regional effects. We have updated the Methods to state this explicitly (**p. 22, lines 19–36**, see also the acquisition/analysis details noted in Q2-2).

Q2-4: Do you see the increase in ISCs and EBs throughout the whole midgut, or is it limited to R4/R5?

We observed increase in ISC proliferation across the entire gut, with the largest effect in the R4–R5. Accordingly, ISC/EB imaging/quantification was performed in R4-R5 to capture the maximal regional response. Outside R4–R5, the increase was present but more modest.

Q2-5: What happens to EE cells upon VB5 supplementation?

EE accounts for a small portion of the whole gut cell population (~10%), with a major role in hormone secretion, which is not very relevant in our current story, therefore we did not check EE cells. We thank for the suggestion of investigating the changes in EE population upon VB5 supplementation, which is an interesting question and could be followed up in our future studies.

Q3: The possibility of the gut directly absorbing VB5 and synthesizing CoA has not been properly addressed, and it is a key point: Have the authors tried to knockdown Fbl specifically in ECs and check its effect on VB5-induced ISC proliferation? Is it possible that interfering VB5 absorption from ECs can have a non-cell-autonomous effect on ISC proliferation? In Extended Data Fig. 1b, knockdown of Fbl in ISCs and EBs surprisingly promoted ISC proliferation upon VB5 feeding, what is the possible explanation of this? Would it cause a similar increase in homeostatic ISC proliferation (without VB5 supplementation) since it is not shown in the figure?

Thank you for the insightful comment. Here are our responses to your questions.

Q3-1: Have the authors tried to knockdown Fbl specifically in ECs and check its effect on VB5-induced ISC proliferation? Is it possible that interfering VB5 absorption from ECs can have a non-cell-autonomous effect on ISC proliferation?

We agree with you that it is possible VB5 absorbed by ECs could non–cell-autonomously stimulate ISC proliferation. Following your suggestion, we tested whether EC-intrinsic CoA biosynthesis is required for the VB5 response by knocking down *Fbl* specifically in ECs and

feeding VB5-supplemented diets. Quantification of pH3$^{+}$ cells across the gut showed that EC-specific *Fbl* knockdown did not block VB5-induced ISC proliferation, suggesting interfering CoA biosynthesis in the EC cells do not affect ISC proliferation. Consistent with this, our whole-body snRNA-seq dataset [4] indicated that the VB5 transporter *Smvt* was minimally expressed in the ISCs and ECs but highly enriched in MT principal cells, arguing against substantial EC uptake of VB5 for *de novo* CoA biosynthesis. We have added these results to **Extended Data Fig.1e-f** and clarified the text at **p. 6, lines 37-38; p. 7, lines 1-10**:

*"Next, we knocked down Fbl in the gut using the temperature-sensitive EGT-Gal4 (EGTts>) or Myo1A-Gal4 (Mexts>) driver to test whether gut-intrinsic CoA biosynthesis is required for VB5-induced ISC proliferation. Intriguingly, Fbl knockdown in neither ISC/EBs nor ECs suppressed VB5-induced ISC proliferation (Extended Data Fig. 1e). In contrast, these flies exhibited elevated baseline level of pH3+ signal, and VB5 supplementation further increased the ISC proliferation. This elevated baseline proliferation likely reflects metabolic imbalance and gut damage caused by loss of Fbl function in the gut. In addition, our previous whole-body single-nucleus RNA-seq (snRNA-seq) analysis revealed that VB5 transporter Smvt (the Drosophila homolog of SLC5A6/SMVT), which is known to stimulate CoA biosynthesis [17], was minimally expressed in the ISCs and ECs (Extended Data Fig. 2f) [39]. Collectively, these results suggest that gut-intrinsic CoA biosynthesis may not be required for VB5-induced ISC proliferation."*

Furthermore, in the Yki tumor context, *Smvt* and *Fbl* are induced in MT principal cells but not in ISCs (**Extended Data Fig. 3a**). Additional analysis of the same dataset shows no induction of *Smvt* or *Fbl* in ECs of Yki flies, either, reinforcing an MT-specific role for CoA production under conditions of elevated CoA demand. We updated **Extended Data Fig. 3a** to display gene expression across ISC, EC, and principal-cell populations in Yki flies.

Altogether, these data indicate that renal, rather than gut-intrinsic, CoA biosynthesis is the primary driver of ISC proliferation in response to VB5 supplementation.

Q3-2: In Extended Data Fig. 1b, knockdown of Fbl in ISCs and EBs surprisingly promoted ISC proliferation upon VB5 feeding, what is the possible explanation of this? Would it cause a similar increase in homeostatic ISC proliferation (without VB5 supplementation) since it is not shown in the figure?

We did test baseline conditions (without VB5 supplementation). *Fbl* knockdown in the ISC/EBs did not significantly increase homeostatic ISC proliferation; however, we noticed that EC-specific *Fbl* knockdown elevated baseline proliferation. Nevertheless, under VB5 supplementation, *Fbl* knockdown in either ISCs/EBs or ECs further increased ISC proliferation. A parsimonious explanation is that loss of PanK activity perturbs CoA pools and lipid/energy metabolism, eliciting stress that secondarily stimulates ISC divisions (e.g., JNK/Upd/EGF stress signaling and compensatory regeneration). We will follow up this interesting observation and dissect more of intestinal Fbl function in the future studies. We

included these new results in **Extended Data Fig. 1e** and edited the text at **p. 5, lines 37-38; p. 6, lines 1-5** (see also details noted in Q3-1).

Q4: Have you checked the changes in the protein levels of Fbl or dPANK4 upon VB5 supplementation?

Thank you for this suggestion. We agree that assessing protein levels of CoA-pathway enzymes after VB5 supplementation would further strengthen our conclusions. At present, we are limited by the availability of suitable antibodies/reagents and therefore cannot perform these measurements. Nevertheless, multiple independent lines of evidence support activation of the CoA biosynthetic pathway with VB5: (i) VB5 increases/decreases expression of key CoA-pathway genes **(Fig. 2e)**; (ii) functionally, VB5 deprivation abrogates the ISC-proliferative effect caused by *dPank4* knockdown, indicating VB5 dependence **(Fig. 2h)**; and (iii) VB5 isotope-tracing demonstrates elevated CoA production from VB5 in *dPank4* knockdown **(Fig. 2l)**. We noted protein-level validation as a priority for our future work.

Q5: What is the MT-gal4 driver? What cells are being targeted? Unless I am missing something, I cannot see this very important information in the description of the experimental work and results. Related to this, have you checked the effects of knocking down Fbl in MTs on VB5-induced ISC proliferation? In addition, have you tried to check whether overexpressing Fbl in MTs is also sufficient to increase ISC proliferation?

Thank you for your comments. Here are our responses to your questions.

Q5-1: What is the MT-gal4 driver? What cells are being targeted? Unless I am missing something, I cannot see this very important information in the description of the experimental work and results.

We agree that the information of the MT-Gal4 is very important for our manuscript and have described this driver in Results, Methods, and Supplemental Table of the manuscript with related reference cited:

- In the **Results (p.7, lines 23-25)**, *"we knocked down dPANK4 using the temperature-sensitive, MT principal cell-specific driver (hereafter referred to as MTts>) [42]"*
- In the **Methods-Drosophila husbandry, diet, and strains (p.21, line 34)**, *"tub-Gal80ts, CG31272-Gal4 (MT driver) [42]"*
- In the **Supplemental Table 3- Genotypes used in the study**:
  *2e, f, g, I, j, k; 3a, c, d; S1e, f, g, j; S2a, b c, d, f, g*
  *CG31272 (MT)-GAl4, Tub-GAL80TS > +*

*CG31272-GAl4, Tub-GAL80TS > CG5828 (dPANK4)-RNAi*

This driver was validated in our recent study, which was cited:

*Reference [42]: Xu, J., et al., Mechanistic characterization of a Drosophila model of paraneoplastic nephrotic syndrome. Nature Communications, 2024. 15(1).*

Q5-2: Related to this, have you checked the effects of knocking down Fbl in MTs on VB5-induced ISC proliferation? In addition, have you tried to check whether overexpressing Fbl in MTs is also sufficient to increase ISC proliferation?

We did not specifically test whether manipulating *Fbl* alters ISC proliferation under VB5 supplementation. Our results showed that across conditions that elevate nutrient precursor availability or growth signaling, or increase CoA demand, *dPank4* showed a markedly stronger transcriptional response than *Fbl* **(Fig. 2e; Fig. 2i; Fig. 5n-o)**, suggesting that dPANK4 plays the dominant regulotory role in renal CoA production. In addition, Fbl (PanK) function in *Drosophila* CoA biosynthesis is well characterized, whereas dPank4 (CG5828) had not been functionally defined. We therefore focused on dPank4 to probe pathway activation or inhibition. Notably, in the tumor context where *Fbl* is robustly induced **(Fig. 5a)**, MT-specific *Fbl* knockdown reduced ISC/tumor cell proliferation **(Fig. 4c-f; Extended Data Fig. 3b-i)**. We appreciate your comments, and we believe our current results support a role for MT CoA biosynthesis in promoting ISC proliferation.

Q6: related to the following statement by authors: “These mitotic signals first appeared in the posterior R4-R5 region, which lies adjacent to the MT-gut junction, and progressively spread anteriorly upon prolonged dPANK4 knockdown (Fig. 1a and Extended Data Fig. 2g).” The images shown here (Extended Data Fig. 2g) are not clear enough to prove that the pro-mitosis signal is “progressively spreading anteriorly”. There should be at least quantifications of pH3+ cells for both the anterior and posterior midguts at different time points following dPANK4 KD induction to demonstrate that pro-proliferative signal originates locally at the R5-MT junction rather than being disseminated through circulation.

Thank you for your comments. It is a very good suggestion to quantify the mitosis signal at anterior and posterior midguts to strengthen our statement. As suggested, we quantified the pH3+ signal of *dPANK4* knockdown flies at day 5 and day 9 and included the results in **Extended Data Fig. 2l**. We also improved the images in **Extended Data Fig. 2k** for better visualization.

Q7: According to Cabrero et al. (Reference 45), Drip protein is only highly expressed in stellate cells. In addition, the snRNA-seq data (Reference 35) also showed that Drip expression is very minimal in principal cells except for lower ureter PCs. I don’t know why

the expression data you showed in Extended Data Figure 2h seems to be very different from the published one, although you used the same dataset. Could you please explain why knocking down Drip in principal cells, where it is not highly expressed, could have such a strong effect on inhibiting VB5-induced proliferation, whilst the knockdown in stellate cells almost had no effects?

Thank you for the comment. Here are our responses to your concerns and questions.

Q7-1: According to Cabrero et al. (Reference 45), Drip protein is only highly expressed in stellate cells. In addition, the snRNA-seq data (Reference 35) also showed that Drip expression is very minimal in principal cells except for lower ureter PCs. I don't know why the expression data you showed in Extended Data Figure 2h seems to be very different from the published one, although you used the same dataset.

Cabrero et al. examined the localization of aquaporin proteins by antibody-based immunostaining, whereas our analysis reports mRNA expression of *Drip* from an MT snRNA-seq dataset. While immunostaining directly demonstrates the protein level, snRNA-seq is more sensitive thus can better detect lower levels of *Drip*. This difference in detection modality can reasonably account for minor inconsistencies between studies.

We also thank you for pointing out the discrepancy between our figure and the previously published version. The updated panel in our manuscript (**Extended Data Fig. 2m**) was generated using the recently established FlyRNAi RNA-seq data distribution and analysis portal (https://www.flyrnai.org/tools/rna_seq_base/web/showProject/38/plot_coord=1/sample_id=all)
, which applies a slightly different processing and visualization pipeline. Nevertheless, the overall expression pattern is consistent across the majority of cell types: in both representations, *Drip* is most enriched in stellate cells and is readily detectable in multiple principal cell (PC) subtypes, including main-segment PC and lower-ureter PC. We have noted the specific data source and analysis tools in the Methods section at **p. 26, lines 10-11**:

*"Cell-type-specific expression data were sourced from the DRSC/Perrimon Lab single-cell RNA-seq portal."*

Q7-2: Could you please explain why knocking down Drip in principal cells, where it is not highly expressed, could have such a strong effect on inhibiting VB5-induced proliferation, whilst the knockdown in stellate cells almost had no effects?

Regarding the reason why principal cell-specific knockdown of *Drip* blocks VB5-induced ISC proliferation despite low baseline *Drip* in this cell type, we hypothesize that VB5 feeding selectively upregulates *Drip* expression in principal cells. This is consistent with our evidence that VB5 transport and CoA-pathway induction is principal cell-centric and

sufficient to modulate VB5-dependent ISC proliferation shown in **Fig.2**, **Fig. 4, Extended Data Fig.1** and **Extended Data Fig.3**.

Q8: How would knocking down Drip in principal cells affect homeostatic ISC proliferation (without VB5 feeding)? Is it possible that Drip is required for its role other than transporting metabolites? Apart from Drip, any other aquaporins might be involved in this? Have you checked the levels of CoA-related metabolites in the guts when Drip is knocked down in MTs by VB5 isotope tracing?

Thank you for the comment. Here are our responses.

Q8-1: How would knocking down Drip in principal cells affect homeostatic ISC proliferation (without VB5 feeding)? Is it possible that Drip is required for its role other than transporting metabolites?

We did test the role of Drip in homeostatic ISC proliferation without VB5 supplementation. Our results showed that knocking down *Drip* in principal cells did not alter pH3 signal, suggesting that *Drip* knockdown does not affect homeostatic ISC proliferation. We included the results in **Extended Data Fig. 2n**.

Q8-2: Apart from Drip, any other aquaporins might be involved in this?

We also assessed the role of another well-established aquaporin Prip in this MT-gut interaction. Knockdown of Prip in either principal cells or stellate cells blocked VB5-induced ISC proliferation, and Prip knockdown didn't influence the baseline ISC proliferation. These observations are consistent with a previous report showing that Drip, rather than Prip, mediates renal-gut countercurrent flow that delivers renal-derived cytokines to the gut [5]. These data suggest that CoA-derived metabolites reach the gut from the MTs with Drip-dependent water influx. We included these results in the revised manuscript **(Fig. 3f; Extended Data Fig. 2n-p; p. 11, lines 12-26)**:

*"To determine whether aquaporin-mediated water influx is involved in the inter-organ metabolite exchange, we knocked down Drip or Prip in stellate cells (tshts>) or principal cells (MTts>). Knockdown of either aquaporin in these MT cell types did not affect baseline ISC proliferation (Extended Data Fig. 2n-o). In line with the role of CoA biosynthesis activation in principal cells in driving ISC proliferation (Fig. 2f-h and Extended Data Fig. 1i-k), knockdown of Drip in principal cells completely abolished VB5-induced ISC proliferation (Fig. 3f). In contrast, knockdown of Drip in stellate cells had no effect (Extended Data Fig. 2o). However, knockdown of Prip in neither stellate cells nor principal cells blocked VB5-induced ISC proliferation (Fig. 3f; Extended Data Fig. 2p). These observations are consistent with previous reports showing that Drip, rather than Prip, mediates renal-gut countercurrent flow that delivers renal-derived cytokines to the gut*

*[52]. Altogether, these results suggest that CoA-dependent metabolites could reach the gut from the MTs through Drip-mediated water influx, which facilitates bulk fluid flow and enhances delivery, thereby activates intestinal MVA and isoprenoid backbone biosynthesis pathways to promote ISC proliferation."*

Q8-3: Have you checked the levels of CoA-related metabolites in the guts when Drip is knocked down in MTs by VB5 isotope tracing?

We did not perform isotope tracing in gut samples upon Drip knockdown. Our platform can trace VB5 into CoA, some CoA biosynthesis intermediates, and several acyl-CoAs, but it does not currently resolve metabolites or lipids in the mevalonate/isoprenoid pathway, the downstream intestinal products that promote stem/tumor cell proliferation. In addition, tracer studies on dissected tissues are technically demanding and costly, requiring large input material and substantial tracer amounts. Given these limitations, we are not able to conduct this experiment at present. We agree it would provide valuable support for the model and have added this point to the **Limitations of the study**. Nevertheless, we have convergent results showing intestinal isoprenoids production acts downstream of renal CoA biosynthesis to drive ISC proliferation **(Fig. 3 and Extended Data Fig. 2)**, supporting a model of aquaporin-mediated MT-gut metabolite exchange.

Q9: related to the following statement by authors: "We observed significant transcriptional changes in CoA biosynthesis genes, Fbl and dPANK4, specifically in the MT principal cells, but not in the gut ISCs/tumors (Fig. 4a and Extended Data Fig. 3a), suggesting a potential contribution of MT-derived CoA synthesis to gut tumor growth."
Have you tried to validate the gene expression of Fbl and dPANK4 in principal cells and ISCs by doing qRT-PCR on sorted cells, or even dissected tissues?

Thank you for the suggestion. We did not perform qRT-PCR validation from sorted cells for the two genes. Instead, we used cell-type–specific genetic perturbations, which provide more direct evidence than transcript measurements. As addressed in Q5, we used a MT principal cell-specific driver in this study. In the Yki tumor model, knocking down *Fbl* or overexpressing *dPANK4* specifically in the principal cells reduced tumor growth, tumor-related bloating phenotype, and extends lifespan **(Fig. 4c-i; Extended Data Fig. 3b-j)**. We tested two independent RNAi lines in the Yki background for each gene knockdown/overexpression and examined tumor-related phenotypes at two time points (days 3 and 5 post–tumor induction). Collectively, these consistent results provide strong evidence that restricting CoA biosynthesis specifically in principal cells attenuates tumor expansion and ISC proliferation.

Q10: related to the following statement by authors "Both interventions significantly

suppressed tumor growth, as evidenced by reduced gut width, decreased Yki-GFP signal, and lower mitotic (pH3+) cell counts (Fig. 4c-f and Extended Data Fig. 3b-i)."
The corresponding figures are really confusing. I can see that Extended Figure 3 f-j validated the same thing using a different RNAi, but it seems that Extended data figure 3 b-e showed the same experiments again as Figure 4c-f?
Another question is why there are no significant changes in the gut width in extended figure 3c, while the difference is quite big in figure 4d and extended figure 3g?

Thank you for the comment. Here are our responses.

Q10-1: The corresponding figures are really confusing. I can see that Extended Figure 3 f-j validated the same thing using a different RNAi, but it seems that Extended data figure 3 b-e showed the same experiments again as Figure 4c-f?

**Extended Data Fig. 3b-e** presents phenotypes at day 3 post tumor induction, whereas **Fig. 4c-f** shows phenotypes at day 5 post tumor induction. We had noted this in the legend of **Fig. 4** ("*See also Extended Data Fig. 3 for phenotypes at day 3 and validations using independent fly lines*"). To prevent confusion, we have explicitly labeled all time points in the revised figures.

Q10-2: Another question is why there are no significant changes in the gut width in extended figure 3c, while the difference is quite big in figure 4d and extended figure 3g?

**Extended Data Fig. 3b-e** captures early tumor progression at day 3, where gut width has not yet expanded significantly. By at day 5 **(Fig. 4c–f)**, the phenotype is pronounced, reflecting expected tumor progression.

Q11: Knocking down Fbl in MTs significantly extended the survival of yki tumour flies. Have you observed the same thing with MT-specific dPANK4 overexpression?

Thank you for the comments. MT-specific *dPANK4* overexpression produced a negligible survival benefit in Yki tumor–bearing flies (Kaplan–Meier, log-rank $p = 0.0699$). The effect was most evident early: in Yki flies, mortality began at day 4 with 19% dead by day 10, whereas with *dPank4* overexpression, mortality onset shifted to day 10 with 9% dead at day 10 (results attached below).

We quantified induction efficiency and observed an approximately 6-fold increase in *dPank4* transcripts after 3 days of overexpression (qRT-PCR; results attached below). We anticipate that prolonged overexpression is detrimental and may elicit side physiological effects. Consistent with expectations that chronic suppression of the CoA pathway can impair tissue maintenance, *dPank4* overexpression restricted ISC proliferative capacity required for epithelial renewal (**Fig. 2j; Extended Data Fig. 1l–m**). Accordingly, the modest

survival benefit observed in Yki flies with *dPank4* overexpression is likely limited by these side effects of sustained CoA-pathway suppression.

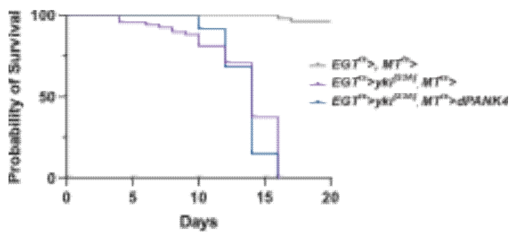


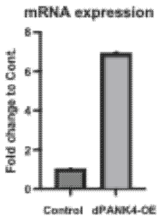


Q12: Have you tested whether CoQ/dolichol might also be a downstream pathway of MVA?

Thanks for the comments. Following your suggestion, we examined whether CoQ and dolichol biosynthesis contribute to the proliferative response downstream of renal CoA/MVA activation as well. Dietary supplementation with 4-nitrobenzoic acid (4-NB), a competitive inhibitor of CoQ biosynthesis, significantly suppressed the elevated pH3$^+$ signal in the gut of *MTts>dPANK4RNAi* flies, suggesting that CoQ biosynthesis is required for ISC proliferation induced by enhanced renal CoA production. Consistently, gut-specific knockdown of *qless* (fly homolog of PDSS1), a CoQ biosynthesis enzyme, completely abolished VB5-induced ISC proliferation. Because no specific pharmacological inhibitor of dolichol biosynthesis is available, we instead genetically perturbed this pathway by knocking down *Pprd* (Polyprenal reductase, fly homology of SRD5A3). Inhibition of dolichol biosynthesis in the gut modestly attenuated VB5-induced ISC proliferation. These findings are consistent with our analysis on human kidney cancer dataset, where we showed that higher expressions of enzymes in CoQ and dolichol biosynthesis pathway significantly correlates with shorter survival of pRCC patients (**Extended Data Figure 5**). We included these results in **Extended Data Figure 2h-j** and clarified the text at **p.10, lines 16-33**:

*"We also tested the potential involvement of CoQ and dolichol biosynthesis, two additional major downstream outputs of MVA-isoprenoid pathway (Fig. 3b). Dietary supplementation with 4-nitrobenzoic acid (4-NB), a competitive inhibitor of CoQ biosynthesis (Fig. 3b) [51] significantly suppressed the elevated pH3$^+$ signal in the gut of MTts>dPANK4RNAi flies (Extended Data Fig. 2h-i), suggesting that CoQ biosynthesis is required for ISC proliferation induced by enhanced renal CoA production. Consistently, gut-specific knockdown of qless (fly homolog of PDSS1), a CoQ biosynthesis enzyme, completely abolished VB5-induced ISC proliferation (Extended Data Figure 2j). Because no specific pharmacological inhibitor of dolichol biosynthesis is available, we instead genetically perturbed this pathway by knocking down Pprd (Polyprenal reductase, fly homology of SRD5A3). Inhibition of dolichol biosynthesis in the gut modestly attenuated VB5-induced ISC proliferation (Extended Data Figure 2j). Collectively, these results demonstrate that MT-derived CoA pool influences gut homeostasis through the MVA-isoprenoid biosynthesis pathway. Multiple downstream outputs of this pathway, including Rho prenylation-mediated YAP/TAZ activation, as well as*

*CoQ and dolichol biosynthesis, are likely engaged upon increased isoprenoid production and contribute to the stimulation of ISC proliferation."*

In addition, in our initial submission, we established protein prenylation as a key downstream effector of renal CoA/MVA-driven proliferation (**Fig. 3e**). In the revision, we further explored the consequences of protein prenylation and found that prenylated signaling proteins, such as Rho GTPases, are likely activated in the gut in response to increased isoprenoids flux, leading to YAP/TAZ activation and ISC proliferation. This is consistent with prior reports that YAP/TAZ promote cell proliferation and organ overgrowth in *Drosophila* [6]. We included these results in **Extended Data Figure 2g** and clarified the text at **p.10, line 5-16**:

*"Previous studies have shown that β-GGT-I-mediated prenylation activates Rho GTPases, which are required for the activation of the Hippo pathway mediators YAP and TAZ (YAP/TAZ) and thereby promote cell proliferation and organ overgrowth in Drosophila [49]. To test whether YAP/TAZ signaling is activated upon enhanced renal CoA production, we examined the expression of two well-established target genes of Yorkie (Yki; the Drosophila YAP/TAZ homolog), Diap1 and expanded (ex) [49, 50] in the gut of MTts>dPANK4RNAi flies. Consistent with prior studies showing downregulation of these genes upon inhibition of β-GGT-I-mediated prenylation [49], we observed significant upregulation of both Diap1 and ex (Extended Data Fig. 2g). These findings suggest that prenylated signaling proteins, such as Rho GTPases, may be activated in the gut in response to increased isoprenoids output driven by renal CoA biosynthesis, leading to enhanced YAP/TAZ activity and ISC proliferation."*

Together, these mechanistic dissections show that elevated isoprenoid production downstream of CoA biosynthesis engages multiple effector branches, including prenylation, CoQ biosynthesis, and dolichol biosynthesis. These findings are highly concordant with our analyses of human pRCC datasets (**Fig. 6 and Extended Data Fig. 5**) and provide new mechanistic insight into the CoA-isoprenoid axis in tissue homeostasis and cancer progression.

Q13: Is it possible to identify the metabolites transported between MTs and guts that mediate the role of MT CoA biosynthesis in ISC proliferation

Thanks for the comments. We agree this is an important question. At present, technical constraints preclude definitive identification of the transported metabolites. We have outlined testable directions in the Discussion. Briefly, 4'-phosphopantetheine (a VB5-derived CoA precursor) is a plausible candidate because it can circulate systemically and could be imported by recipient tissues to "jump-start" CoA biosynthesis. Another possibility is that acyl-CoAs generated from CoA by acyl-CoA synthetases, enabling redistribution of CoA-dependent metabolites through transportable intermediates such as

fatty acids or acyl-carnitines. We have added this as a in the **Limitations of the study** and will address this import question in future work.

Q14: Have you checked whether CoA biosynthesis is also playing a conserved role in human gastrointestinal cancer patients?

Thank you for the insightful comment. We indeed examined multiple common cancers (breast, liver, and gastrointestinal) and only found strong clinical association of the CoA–MVA–isoprenoid axis occurs in papillary renal cell carcinoma (pRCC) **(Fig. 6)**. Notably, the same association is not evident in the closely related kidney cancer subtype, clear cell RCC (ccRCC). pRCC preserves tubular architecture and is therefore more structurally and functionally similar to renal tubules, the mammalian counterpart of *Drosophila* Malpighian tubules, and this makes our MT findings particularly relevant to this subtype. These results underscore cancer-type specificity in metabolic rewiring. Given that pRCC remains under-studied and lacks subtype-specific therapies (current regimens are largely adapted from ccRCC), our data provide mechanistic insight that could inform pRCC-targeted strategies.

Q15: The physiological role and expression domain of Myc in the system requires further experimentation to substantiate the conclusions/working model. a) Where in the MT is Myc upregulated? This should be addressed using Myc antibody staining combined with markers of MT cell sub-types. It is highly surprising that Myc from the tubules is sufficient to decrease ISC proliferation in Yki tumours. I would expect Yki would upregulate Myc in the gut itself. Have the authors investigated this? This is very important information missing. B) I would be very cautious with the interpretation of the Myc overexpression results presented in Fig 5d,e. Because of the lack of information on the MT-gal4 driver (Q5) I can't tell where Myc is being overexpressed. Ectopic overexpression of Myc at high levels highly toxic to cells and can trigger apoptosis https://www.sciencedirect.com/science/article/pii/S153561080800370X. We have experienced the same in flies (non-published work by Reviewer). As such, close analysis of the impact of this manipulation to MT physiology and organismal function is essential. For example, the hyperproliferation of the gut upon MT overexpression of Myc can be secondary to damage in the tubule leading to the production of stress signals that remotely activate gut hyperproliferation. Furthermore, in all experiments involving loss of function of Myc in the paper, there is no analysis of the effect of that alone in the tubules, whole animal and gut. In other words, MT>MycRNAi only controls need to be included in the study.

Thank you for the insightful comment. Here are our responses.

Q15-A: Where in the MT is Myc upregulated? This should be addressed using Myc antibody staining combined with markers of MT cell sub-types. It is highly surprising that Myc from the tubules is sufficient to decrease ISC proliferation in Yki tumours. I would expect Yki would upregulate Myc in the gut itself. Have the authors investigated this? This is very important information missing.

We appreciate the suggestion of the Myc immunostaining experiments. We attempted MT staining with anti-Myc antibody (DSHB- P4C4-B10), but signal quality was insufficient for reliable cell-type assignment. We therefore did not include these images. To address this, we analyzed our previously generated MT snRNA-seq dataset from Yki flies, we found that *Myc* is upregulated across multiple MT cell types, with the strongest induction in principal cells. In contrast, *Myc* transcription was not increased in ISCs or ECs of the guts in Yki flies, in agreement with the unchanged expression of *Fbl*, *dPANK4*, and *Smvt* in the gut **(Extended Data Fig. 3a)**. These patterns support a principal-cell–centric role for Myc in modulating pathways relevant to CoA metabolism in the tumor context and is consistent with our functional validation that knocking down *Myc* specifically in the principal cells reduced tumor growth and bloating, and extends survival of tumor-bearing flies **(Fig. 5h-5m)**.  Given the robust and cell-resolved snRNA-seq evidence together with functional genetics, we view the transcriptomic data as an appropriate line of evidence here. Altogether, our data suggest a principal-cell-centric Myc-CoA pathway model in gut cell proliferation/tumor expansion. These new results about cell-type specificity are included to the revised manuscript **(Extended Data Fig. 4a, c)** and we clarified in the text at **p.13, lines 15-21**:

*"Consistently, analysis of MT snRNA-seq data revealed that Myc expression was elevated in the principal cells and stellate cells of the MTs in Yki flies (Extended Data Fig. 4a) [42]. In addition, TF regulon analysis identified Myc as the most significantly activated TF in the MTs of Yki flies (Extended Data Fig. 4b) [39]. Notably, Myc transcription was not increased in ISCs or ECs of the guts in Yki flies (Extended Data Fig. 4c) [39], in agreement with the unchanged expression of CoA biosynthesis genes in the gut (Extended Data Fig. 3a)."*

Q15-B: I would be very cautious with the interpretation of the Myc overexpression results presented in Fig 5d,e. Because of the lack of information on the MT-gal4 driver (Q5) I can't tell where Myc is being overexpressed. Ectopic overexpression of Myc at high levels highly toxic to cells and can trigger apoptosis https://www.sciencedirect.com/science/article/pii/S153561080800370X. We have experienced the same in flies (non-published work by Reviewer). As such, close analysis of the impact of this manipulation to MT physiology and organismal function is essential. For example, the hyperproliferation of the gut upon MT overexpression of Myc can be secondary to damage in the tubule leading to the production of stress signals that remotely activate gut hyperproliferation. Furthermore, in all experiments involving loss of function of Myc in the paper, there is no analysis of the effect of that alone in the tubules, whole animal and gut. In other words, MT>MycRNAi only controls need to be included in the study.

The questions regarding the MT driver are addressed in Q5. We thank the reviewer for including the reference paper. This paper examines role of Akt in cellular senescence and ROS-mediated apoptosis and shows that Akt activation can accelerate senescence and sensitize cells to apoptosis. We did not, however, find evidence in that work for the specific claim that "*ectopic overexpression of Myc at high levels is highly toxic and triggers apoptosis*".

We appreciate the reviewer's point and have reviewed additional literature on MYC and apoptosis. We agree that MYC can elicit apoptosis in certain contexts [7] and therefore assayed apoptosis markers following MT-specific *Myc* overexpression. In dissected MTs, transcripts of canonical apoptosis genes (*rpr*, *hid*, *grim*, *skl*) [8] did not show induction: *rpr* and *hid* were unchanged, *grim* was reduced, and *skl* was undetectable in both control and *Myc*-overexpressing MTs. These data do not support MYC-induced apoptosis in MTs under our conditions. We included these results in **Extended Data Fig. 4d** and clarified in the text at **p. 13, lines 35-40; p.14, lines 1-5**:

*"Because Myc overexpression of can trigger apoptosis in certain conditions [55], it is possible that MT apoptosis releases stress signals to the gut and in turn drives ISC proliferation. We therefore examined the expression of canonical apoptosis-inducible genes in the MTs, including reaper (rpr), hid, grim, and sickle (skl), which are transcriptionally activated in response to diverse pro-apoptotic stimuli [56]. We found that the expression of rpr and hid was unchanged upon Myc overexpression, whereas grim expression was reduced (Extended Data Fig. 4d). skl was undetectable in the MTs of either control or Myc-overexpressing flies. These data suggest apoptosis is not induced by Myc overexpression in the MTs in our experiments, supporting the model that Myc promotes ISC proliferation primarily through activation of CoA biosynthesis rather than secondary tissue damage."*

Moreover, MYC-driven apoptosis is context-dependent, typically requiring co-factors such as limited survival signaling or intact p53/ARF checkpoints. In settings with strong pro-growth cues (e.g., PDGF/VEGF), MYC predominantly drives proliferation and metabolic reprogramming [9-11]. This is consistent with our model in which PDGF/VEGF signaling upregulates *Myc* in the MTs.

We also thank the reviewer for the suggestion on including *Myc*-RNAi only as a control. We attempted *Myc* knockdown in MTs of wild-type flies using three RNAi lines (BDSC 43962, 25783, 36123). None achieved robust knockdown in our hands, likely reflecting low baseline *Myc* expression in MTs under homeostasis (**Extended Data Fig. 4c**).

Altogether, our data suggest apoptosis may not be induced by *Myc* overexpression in the MTs, supporting the model that Myc promotes ISC proliferation primarily through activation of renal CoA biosynthesis rather than secondary tissue damage."

Materials and Methods:

In general, the number of flies used for pH3 or GFP quantification is too low.

Thank you for the comment. For pH3 and GFP quantification, we analyzed 6-18 guts per condition, sample sizes that are standard in *Drosophila* gut studies [12, 13]. To ensure robustness, all key experiments were repeated with at least one independent line and evaluated at multiple time points (e.g., three *UAS-dPANK4 RNAi* lines, five time points measured, both males and females tested; two *UAS-dPANK4* lines; two *EGT>yki; MT>dPank4* lines, two time points measured; two *EGT>yki; MT>fbl-RNAi* lines, two time points measured). Across these validations, the effects were consistent in direction and magnitude. Together with appropriate statistical testing, these controls support the strength of our conclusions.

For VB5 isotope tracing experiments, have you checked the labelling efficiency at 48h?

Thanks for the comments. The labeling efficiency is over 90%. The results shown in Fig.2l was normalized against labeling efficiency of each group.

There is a lack of detailed description on quantification/analysis methods in general. For example, how did you measure the gut width in the R4/R5 region? Did you perform a single measurement at a randomly selected section within this region, or did you take three to five measurements across the R4/R5 and calculate the mean value? In addition, how did you exactly measure the number of GFP+ cells in the gut? Because it does not make sense to quantify "by measuring the average GFP signaling strength from individual flies". Please clearly state the approach used, including how ROIs were selected and whether averaging was applied to ensure accuracy and reproducibility.

Thanks for the comments. We have included more details in the Methods (**p. 22, line 19-36,** see also Q2 for more details).

For confocal imaging, there is no description on imaging parameters. Excitation/emission wavelengths? Z-stacks or single plane? If you also did tile scan, what is the relative parameters?

Thanks for the comments. We have included more details in the Methods **(p. 22, lines 19–36,** see also Q2 for more details**).**

For lifespan experiment, which day is considered to be Day 0? Did you start to count the death of flies immediately after you transfer flies to 29 °C, or you started to count a few days after transgene induction? How often did you flip the flies onto fresh food and how many flies were in each vial?

Thanks for the comments. We have included more information in the Methods (**p.21, lines 18-20; p.24, lines 24-25**):

*"Unless stated otherwise, twelve female flies and three male flies were put in one vial and were flipped to fresh food every other day unless noted."*

*"Survival was scored daily starting on day 0 at the same time each day."*

Please also note my comments on genetic drivers used in some of the points above.

Thanks for the comments. We have addressed your question regarding the driver in Q5.

Minor comments:

Figure 1 legend- Line 17, 1) it is not the GFP intensity that should be quantified here; 2) Figure 1 f' VB5 condition has 7 dots on the plot but the n stated here is 4-6. Please check the n number carefully.

Thanks for the comments. We have corrected these typos in the legends.

Figure 2i and Extended Data Figure 1i, the purple group should be MTts>+, mated

Thanks for the comments. We have corrected the labelings in the figures.

Figure 2g, the genotype should be MTts>dPANK4-RNAi.

Thanks for the comments. We have corrected this typo in the figure.

“Consistently, VB5 levels were significantly reduced in dPANK4 knockdown flies (Extended Data Fig. 1i), suggesting altered CoA synthesis.”
Figure number here should be Extended Data Fig. 1j.

Thanks for the comments. We have corrected this figure number in the text.

“Moreover, there were also elevated labelled fractions of CoA derivatives, including acetyl-CoA, malonyl-CoA, and HMG-CoA (Fig. 2k).”
The sentence here is redundant. Same information has been described just before it.

Thanks for the comments. We have deleted this redundant sentence in the text.

“Additionally, MYC expression showed a strong inverse correlation with PANK4 expression in pRCC patients (Extended Data Fig. 5d)...”
Should be Extended figure 5b.

Thanks for the comments. We have corrected this figure number in the text.

“Notably, these trends were not observed in ccRCC, where PANK4 and PPAT expression did not correlate with survival (Extended Data Fig. 5d-e), and higher PANK3 expression was unexpectedly associated with better outcomes (Extended Data Fig. 5f).”
Figure numbers did not match the results described here.

Thanks for the comments. We have corrected the figure number labeling in the Extended Data Fig. 5.

Reviewer #2 (Remarks to the Author):

This manuscript investigates how a renal–intestinal metabolic axis, mediated by vitamin B5/CoA supply from the Malpighian tubules (MT), regulates intestinal stem cell (ISC) proliferation and tumor growth in Drosophila. The authors show that Myc upregulates CoA biosynthesis in MTs by modulating Fbl and dPANK4, thereby enhancing CoA production. This systemic increase in CoA availability drives mevalonate (MVA) pathway activity in the intestine, promotes isoprenoid biosynthesis, and subsequently enhances ISC proliferation and Yki-driven tumorigenesis. Human data analyses further link MYC and CoA–isoprenoid pathway gene expression to poor prognosis in papillary renal cell carcinoma (pRCC).

The study is conceptually novel, providing a mechanistic model for multi-organ

coordination of metabolite regulation to control stem cell function and tumorigenesis, and will thus be of significance to multiple scientific fields. The integration of tissue-specific genetics, metabolomics, isotope tracing, pharmacology, and cross-species comparative analyses is a major strength and highlights strong potential impact of the data provided in the manuscript. However, there are just a few concerns (experimentally and conceptually) in the current version of this intriguing study.

Thank you very much for your insightful comments, which have guided us to substantially improve the quality of our manuscript. We sincerely appreciate your time and effort. Point-by-point responses are provided below.

Primary Concerns:

1. pH3 staining is used as the primary readout for ISC proliferation, but the mechanistic link between MT-derived CoA and ISC division remains unclear. What are the direct downstream effectors of the isoprenoid pathway in this context that impact ISC proliferation? Is this a specific regulation (targeting distinct growth / proliferation / differentiation pathways) or more general? Demonstrating effects on known prenylated signaling proteins (such as Ras, Rho, Rab, etc.) would strengthen the mechanistic conclusions.

Thanks for the insightful comments. Following your suggestion, we further tested the downstream targets of prenylated signaling proteins. We have shown that gut-specific knockdown of β-GGT-I (prenylation regulator) completely abolished VB5-induced ISC proliferation (Fig. 3e). Previous studies have shown that β-GGT-I-mediated prenylation activates Rho GTPases, which are required for activation of the Hippo pathway mediators YAP and TAZ (YAP/TAZ) and thereby promote cell proliferation and organ overgrowth in *Drosophila* [6]. They found that knockdown of β-GGT-I downregulated the well-established Yki (the *Drosophila* YAP/TAZ homolog) target genes, *Diap1* and *expanded* (*ex*) [6, 14]. Consistently, we observed significant upregulation of both *Diap1* and *ex* in the gut of *MT*$^{ts}$*>dPANK4*$^{RNAi}$ flies, supporting a model in which prenylated Rho-class GTPases activate Yki and drive proliferation. These findings suggest that prenylated signaling proteins, such as Rho GTPases, are activated in the gut in response to increased isoprenoids output driven by renal CoA biosynthesis, leading to enhanced YAP/TAZ activity and ISC proliferation. These data are included in **Extended Data Fig. 2g**, and the text has been clarified on **p.10, lines 5-16**:

*"Previous studies have shown that β-GGT-I-mediated prenylation activates Rho GTPases, which are required for the activation of the Hippo pathway mediators YAP and TAZ (YAP/TAZ) and thereby promote cell proliferation and organ overgrowth in Drosophila [49]. To test whether YAP/TAZ signaling is activated upon enhanced renal CoA production, we examined the expression of two well-established target genes of Yorkie (Yki; the Drosophila*

*YAP/TAZ homolog), Diap1 and expanded (ex) [49, 50] in the gut of MTts>dPANK4RNAi flies. Consistent with prior studies showing downregulation of these genes upon inhibition of β-GGT-I-mediated prenylation [49], we observed significant upregulation of both Diap1 and ex (Extended Data Fig. 2g). These findings suggest that prenylated signaling proteins, such as Rho GTPases, may be activated in the gut in response to increased isoprenoids output driven by renal CoA biosynthesis, leading to enhanced YAP/TAZ activity and ISC proliferation."*

As our analyses of human kidney cancer datasets show that higher expression of enzymes across multiple isoprenoid-output pathways correlates with shorter survival in pRCC (Fig. 6), we therefore also tested whether CoQ and dolichol biosynthesis contribute to the proliferative response downstream of renal CoA production. We knocked down *dPank4* in MT principal cells to activate renal CoA biosynthesis and fed flies 4-nitrobenzoic acid (4-NB), a competitive inhibitor of CoQ biosynthesis. Inhibiting the CoQ pathway markedly suppressed the ISC hyperproliferation induced by *dPank4* knockdown. Consistently, gut-specific knockdown of *qless* (a CoQ biosynthesis enzyme) completely abolished VB5-induced ISC proliferation. Because no specific dolichol inhibitor is available, we knocked down *Pprd* (polyprenol reductase) and found that dolichol pathway inhibition in the gut partially reduced VB5-induced ISC proliferation. Together, these results indicate that CoQ biosynthesis is required and dolichol biosynthesis is contributory to the downstream effects of renal CoA production on ISC proliferation, aligning with the pRCC survival associations in Fig. 6. We included these results in **Extended Data Figure 2h-j** and clarified the text at **p.10, lines 16-28**:

*"We also tested the potential involvement of CoQ and dolichol biosynthesis, two additional major downstream outputs of MVA-isoprenoid pathway (Fig. 3b). Dietary supplementation with 4-nitrobenzoic acid (4-NB), a competitive inhibitor of CoQ biosynthesis (Fig. 3b) [51] significantly suppressed the elevated pH3$^{+}$ signal in the gut of MTts>dPANK4RNAi flies (Extended Data Fig. 2h-i), suggesting that CoQ biosynthesis is required for ISC proliferation induced by enhanced renal CoA production. Consistently, gut-specific knockdown of qless (fly homolog of PDSS1), a CoQ biosynthesis enzyme, completely abolished VB5-induced ISC proliferation (Extended Data Figure 2j). Because no specific pharmacological inhibitor of dolichol biosynthesis is available, we instead genetically perturbed this pathway by knocking down Pprd (Polyprenal reductase, fly homology of SRD5A3). Inhibition of dolichol biosynthesis in the gut modestly attenuated VB5-induced ISC proliferation (Extended Data Figure 2j)."*

Together, these results suggest multiple downstream outputs of this pathway, including Rho prenylation-mediated YAP/TAZ activation, as well as CoQ and dolichol biosynthesis, are likely engaged upon increased isoprenoid production and contribute to the stimulation of ISC proliferation. These findings in flies are consistent with our analyses of human kidney cancer datasets.

2. In the presence of intestinal tumorigenesis, Myc is upregulated in MTs in response to Pvf1–Pvr signaling, reprogramming specific aspects of host metabolism to support tumor growth. The authors also propose that CoA biosynthesis in MTs is activated after mating to support gut homeostasis and renewal (non-tumor conditions). However, dPANK4 appears to exert opposite effects on intestinal cell proliferation before and after mating. This discrepancy requires explanation. Additionally, to improve clarity, “MTts>, mated” should be explicitly included in Fig. 2.

Thank you for pointing this out. The group labels in Fig. 2i were incorrect: the purple bar corresponds to *MTts>, mated*. As shown, mating-induced ISC proliferation is blocked by *dPank4* overexpression in the MTs. We have corrected the labeling in the revised manuscript and regret the confusion.

3. MT-specific knockdown of the aquaporin Drip suppresses tumor-dependent phenotypes, but this is unlikely to represent direct transport of CoA intermediates and more likely reflects general solute transfer and altered fluid dynamics.

Thank you for the comment. We agree that *Drip* knockdown likely affects tumor-dependent phenotypes by altering water flux, solute movement, and fluid dynamics, rather than mediating direct transport of CoA intermediates. Our intent was not to imply that aquaporins convey metabolites; rather, MT water influx through Drip can facilitate bulk/convective movement of soluble metabolites toward the gut. We have revised the text to avoid ambiguity at **p. 11, lines 23-26**:

*“Altogether, these results suggest that CoA-dependent metabolites could reach the gut from the MTs through Drip-mediated water influx, which facilitates bulk fluid flow and enhances delivery, thereby activates intestinal MVA and isoprenoid backbone biosynthesis pathways to promote ISC proliferation.”*

Other Concerns:

Most metabolomic analyses are performed on whole bodies (Fig. 2), which obscures the link between MTs and intestine, and there is no quantification of metabolite B5/CoA levels in intestinal samples after MT-specific attenuation of CoA biosynthesis. Experimentally, it’s unclear why certain metabolomic/metabolite assays were not performed in whole tissues.

Thank you for the comments. We agree that metabolomic analysis to the gut samples would be important to address the MT-gut communication model. In **Fig. 2a**, we showed

the pathway enrichment analysis on the gut samples after MT-specific knockdown of *dPANK4*, which highlighted the isoprenoid backbone biosynthesis as one of the most impacted pathways in the gut. We also performed gut metabolomics analysis in the Yki tumor context, which consistently identified significant enrichment of isoprenoid backbone biosynthesis pathway (**Extended Data Fig. 3k**). As we hypothesized that it is CoA-derived or CoA-dependnet metabolites reach the gut from the MTs, we did not anticipate changes in VB5 or CoA levels in the intestines when renal CoA biosynthesis is activated. Indeed, our data showed accumulation of several isoprenoid metabolites in the intestines of *MT>dPANK4-RNAi* flies, while VB5 or CoA didn't change (attached below; MVA: mevalonate; GPP: geranyl pyrophosphate; FPP: farnesyl pyrophosphate). To avoid confusion, figure panels are now explicitly labeled as gut versus whole-body metabolomics. Together, these results support a model in which renal CoA production activates intestinal MVA/isoprenoid backbone biosynthesis to promote ISC proliferation.

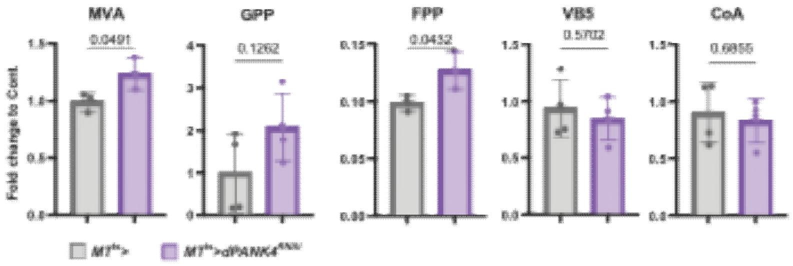


Throughout the manuscript, "EGTGal4" and "GutGal4" are used inconsistently. Please revise for clarity.

Thank you for the comments. We have corrected this and used EGTGal4 for clarity.

Page 6, lines 12–13: "Extended Data Fig. 1I" should be corrected to "Extended Data Fig. 1J."

Thank you for the comments. We have corrected the figure number in the manuscript.

Figure 3J and Page 9, line 32: "including Hmgcr, Fpps, Qm, and β-GGT-I" is misleading, as Hmgcr is not significantly upregulated.

Thank you for the comments. We have removed Hmgcr from the sentence.

The tissue source used in each experiment, especially metabolomic or metabolite

measurement, needs to be clearly stated in figures.

Thank you for the comments. We have stated the tissue source in the figures.

Reviewer #3 (Remarks to the Author):

Dear Editor,

I have now thoroughly read the study by Miao et al. titled 'Renal Coenzyme A (CoA) Production Fuels Stem Cell Proliferation and Tumor Growth'. In this study, the authors uncover a novel axis in which dietary vitamin B5 induces CoA biosynthesis in the Malpighian tubules (MT) of D. melanogaster. In a feedback loop driven by, probably, diffusible metabolites, this leads to growth of intestinal stem cells in the fly gut. Importantly, the increase in CoA production in MTs is induced by the transcription factor Myc. It induces the mevalonate–isoprenoid pathway in the gut, which in turn is important for cell growth. Further, the authors show that this axis, including the involvement of Myc, is important in a subset of human renal cancers and that the mevalonate–isoprenoid pathway is important for the growth of these cancers.

Overall, the authors uncover several very novel pathways; the study is thus impactful and original. It adds significantly to our understanding in terms of cofactor-driven local and inter-organ cellular programmes. There are a few points that need some further consideration. Up front, I would like to note that not all of them need addressing necessarily to meet the impact for this journal, but in some cases, the wording might need to be somewhat adjusted.

The main point is that the axis between the gut and the MT is not as clear as portrayed. In particular, Fig. 5p is not fully backed by data. The authors depict an axis of tumour-derived Pvf1, which activates the Pvr in the MT. This is plausible, but as far as I can tell, the authors never show it directly. Instead, they rely on published data proving Pvf1 can induce Myc in the MT, as well as the utilisation of a constitutively active Pvr in the MT. However, the correct way of showing this would be to have tumours with a knockdown of Pvf1, which should reduce Myc activity and CoA biosynthesis, as well as the mevalonate–isoprenoid pathway. The authors could thus either prove formally that the gut–Pvf1→MT–Pvr axis exists or tone down the wording accordingly.

Thank you for the insightful comment. We agree that we didn't show it directly that tumor-derived Pvf1 activates PvR in the MT in the manuscript. Our prior work demonstrated that tumor-derived Pvf1 activates its receptor Pvr in MTs of Yki flies [15], and in the current study, we showed that MT-specific Pvr knockdown in Yki flies brought the expression of *Myc* and CoA biosynthesis genes to near-baseline levels (**Fig. 5o**), and MT-specific Pvr activation in wild-type flies was sufficient to induce *Myc* and relevant gene expression changes in CoA biosynthesis genes (**Fig. 5n**). To further validate this axis, we

overexpressed *Pvf1* in the gut using EGT (ISC) driver and accessed gene expression of *Myc*, *Fbl*, and *dPANK4* in the MTs. However, the effects of ISC-specific *Pvf1* overexpression were not robust, probably because ISCs are significantly less in the wild-type gut compared with Yki tumor-bearing guts, thereby limiting the overall capacity for Pvf1 secretion. Therefore, we expressed *Pvf1* in the ECs using Mex driver, and we did observe significant transcriptional alterations of those genes, suggesting MT Myc activity can be induced by gut-derived *Pvf1*. These data strengthen the proposed gut–Pvf1→MT–Pvr axis. We have incorporated these new results to **Fig. 5n**, and clarified in the text at **p.14, lines 30-33**.

*"To test if Myc expression is modulated by this axis in the MTs, we overexpressed Pvf1 in the gut of wild-type flies. Intestinal Pvf1 overexpression significantly increased Myc transcription while concomitantly repressing dPANK4 expression in the MTs (Fig. 5n)."*

In the same vein, the notion that it is MT-derived metabolites driving ISC and/or tumour growth in the gut is very plausible but not proven. MT-restricted knockdown of Drip is indeed a good indicator of fluid transfer between the sites, but that can also be true for signalling molecules rather than metabolites. I acknowledge the difficulty in identifying the precise molecules that transmit this signal, so a clear and transparent assessment of the data is, in my opinion, sufficient. Alternatively, as the mevalonate pathway seems to play such a crucial role in this process, intermediates of this pathway could be fed to the animals to test if their presence is sufficient to mediate the same effect. To summarise the two points above, my feeling is that the MT–gut axis clearly exists, but that the manuscript should be very clear about what conclusions can unambiguously be drawn from the current data.

Thank you for the comments. We appreciate the insightful input and agree that signaling molecules, rather than bulk metabolites, might be delivered to the gut. We also agree that identifying the precise molecules is technically challenging. Accordingly, we have added this point as a limitation in the Limitations of the study (**p.21, lines 1-9**) and will address this important question in future work.

*"Limitation of this study. We propose an aquaporin-mediated kidney-gut metabolite exchange model in which CoA-derived metabolites are transported from the MTs to the gut via renal-gut countercurrent flow. However, owing to current technical limitations, we were unable to directly identify or quantify the specific metabolites delivered through this pathway, and we cannot exclude the possibility that signaling molecules are also transported. Thus, the molecular identities and transport dynamics of the CoA-derived metabolites remain unresolved. Nevertheless, our genetic and physiological data support the existence of kidney-gut exchange, and future studies will be needed to definitively characterize the metabolites involved and establish their functional contributions to gut homeostasis."*

We also appreciate the suggestion regarding intermediate-feeding experiments. While treating cultured cells with pathway intermediates is common, we found no reports of MVA/isoprenoid intermediate feeding in *Drosophila*, likely due to low permeability and chemical instability of these compounds. *In vivo* studies instead typically use pharmacological inhibitors to modulate these pathways. We therefore performed chemical feeding assays in combination with genetic perturbations to interrogate the CoA–isoprenoid axis between the MTs and the gut. We believe these complementary approaches provide orthogonal validation and we hope these new results address the reviewer's concerns.

1) We have shown that gut-specific knockdown of β-GGT-I (prenylation regulator) completely abolished VB5-induced ISC proliferation (Fig. 3e). Previous studies have shown that β-GGT-I-mediated prenylation activates Rho GTPases, which are required for activation of the Hippo pathway mediators YAP and TAZ (YAP/TAZ) and thereby promote cell proliferation and organ overgrowth in *Drosophila* [6]. They found that knockdown of β-GGT-I downregulated the well-established Yki (the *Drosophila* YAP/TAZ homolog) target genes, *Diap1* and *expanded* (*ex*) [6, 14]. Consistently, we observed significant upregulation of both *Diap1* and *ex* in the gut of $MT^{ts}$>$dPANK4^{RNAi}$ flies, supporting a model in which prenylated Rho-class GTPases activate Yki and drive proliferation. These findings suggest that prenylated signaling proteins, such as Rho GTPases, are activated in the gut in response to increased isoprenoids output driven by renal CoA biosynthesis, leading to enhanced YAP/TAZ activity and ISC proliferation. These data are included in **Extended Data Fig. 2g**, and the text has been clarified on **p.10, lines 5-16**:

*"Previous studies have shown that β-GGT-I-mediated prenylation activates Rho GTPases, which are required for the activation of the Hippo pathway mediators YAP and TAZ (YAP/TAZ) and thereby promote cell proliferation and organ overgrowth in Drosophila [49]. To test whether YAP/TAZ signaling is activated upon enhanced renal CoA production, we examined the expression of two well-established target genes of Yorkie (Yki; the Drosophila YAP/TAZ homolog), Diap1 and expanded (ex) [49, 50] in the gut of MTts>dPANK4RNAi flies. Consistent with prior studies showing downregulation of these genes upon inhibition of β-GGT-I-mediated prenylation [49], we observed significant upregulation of both Diap1 and ex (Extended Data Fig. 2g). These findings suggest that prenylated signaling proteins, such as Rho GTPases, may be activated in the gut in response to increased isoprenoids output driven by renal CoA biosynthesis, leading to enhanced YAP/TAZ activity and ISC proliferation."*

2) As our analyses of human kidney cancer datasets show that higher expression of enzymes across multiple isoprenoid-output pathways correlates with shorter survival in pRCC (Fig. 6), we therefore also tested whether CoQ and dolichol biosynthesis contribute to the proliferative response downstream of renal CoA production. We knocked down *dPank4* in MT principal cells to activate renal CoA biosynthesis and fed flies 4-nitrobenzoic acid (4-NB), a competitive inhibitor of CoQ biosynthesis. Inhibiting the CoQ pathway

markedly suppressed the ISC hyperproliferation induced by *dPank4* knockdown. Consistently, gut-specific knockdown of *qless* (a CoQ biosynthesis enzyme) completely abolished VB5-induced ISC proliferation. Because no specific dolichol inhibitor is available, we knocked down *Pprd* (polyprenol reductase) and found that dolichol pathway inhibition in the gut partially reduced VB5-induced ISC proliferation. Together, these results indicate that CoQ biosynthesis is required and dolichol biosynthesis is contributory to the downstream effects of renal CoA production on ISC proliferation, aligning with the pRCC survival associations in Fig. 6. We included these results in **Extended Data Figure 2h-j** and clarified the text at **p.10, lines 16-28**:

*"We also tested the potential involvement of CoQ and dolichol biosynthesis, two additional major downstream outputs of MVA-isoprenoid pathway (Fig. 3b). Dietary supplementation with 4-nitrobenzoic acid (4-NB), a competitive inhibitor of CoQ biosynthesis (Fig. 3b) [51] significantly suppressed the elevated pH3$^+$ signal in the gut of MTts>dPANK4RNAi flies (Extended Data Fig. 2h-i), suggesting that CoQ biosynthesis is required for ISC proliferation induced by enhanced renal CoA production. Consistently, gut-specific knockdown of qless (fly homolog of PDSS1), a CoQ biosynthesis enzyme, completely abolished VB5-induced ISC proliferation (Extended Data Figure 2j). Because no specific pharmacological inhibitor of dolichol biosynthesis is available, we instead genetically perturbed this pathway by knocking down Pprd (Polyprenal reductase, fly homology of SRD5A3). Inhibition of dolichol biosynthesis in the gut modestly attenuated VB5-induced ISC proliferation (Extended Data Figure 2j)."*

Together, these results suggest multiple downstream outputs of this pathway, including Rho prenylation-mediated YAP/TAZ activation, as well as CoQ and dolichol biosynthesis, are likely engaged upon increased isoprenoid production and contribute to the stimulation of ISC proliferation. These findings in flies are consistent with our analyses of human kidney cancer datasets.

The data on human tumours presented by the authors are strong, interesting, and harbour therapeutic potential. However, it does not seem to have much to do with the inter-organ axis seen in the fly. Instead, this seems to be a cell-autonomous or at least organ-autonomous effect in the kidneys. In reading the manuscript, the findings from the fly and the human sometimes seem to be a little conflated. I would suggest the authors carefully read the manuscript to point out not only the similarities but also the clear differences between the two systems.

We appreciate your comments and agree that there are body similarities and differences between the two systems.

We have discussed the differences in the Discussion section: *"It is therefore possible that CoA precursor intermediates or CoA derivatives are transported from the kidney to the gut.*

*In Drosophila, the anatomical connection between the MTs and gut enables direct metabolite exchange. In mammals, however, whether renal CoA production contributes to systemic metabolic homeostasis via the circulation or other mechanisms remains an important avenue for future investigation."*

To avoid confusion, we further clarified this in the Results of the revised manuscript at **p. 16, line 38-39; p.17, lines 1-5**:

*"Collectively, analyses on clinical datasets are largely consistent with our Drosophila findings, in which renal CoA biosynthesis promotes intestinal isoprenoid production and activates downstream mechanisms that drive gut tumor expansion. Different from the inter-organ metabolic coordination observed in flies, renal CoA production is likely to activate isoprenoid biosynthesis and utilization locally during pRCC progression."*

The fly Pank4 identified by the authors is likely indeed to have analogous activity. However, the authors never directly show the phosphatase activity. In vitro assays with recombinant proteins are often tricky to establish; however, the authors could use the metabolomics dataset presented under Fig. 2j to verify the levels of the dPANK4 product pantetheine. If their assumption is correct, pantetheine levels should drop in MTTS>dPANK4RNAi flies.

Thank you for the comments. We agree that demonstrating the phosphatase activity of CG5828 (dPANK4) is important given that this enzyme had not been characterized in flies. Accordingly, we expressed and purified recombinant dPANK4 (WT and catalytic mutants) and performed *in vitro* phosphatase assays. CG5828 displayed robust activity toward both the generic phospho-substrate para-nitrophenyl phosphate (PNPP) and the proposed endogenous substrate 4'-phosphopantetheine (p-PaSH), whereas mutation of the predicted catalytic aspartates (D209 or D245) abolished activity. Because human PANK4 and other DUF89 family members require divalent metal cations, we also tested this feature. Consistent with that behavior, CG5828 activity required a divalent cation, with strong activation by $Co^{2+}$ and $Ni^{2+}$, and weaker activation by $Mg^{2+}$ and $Mn^{2+}$; other biologically relevant mono/divalent cations did not support activity. Together, these results establish CG5828 as the functional PANK4 ortholog in *Drosophila*. We have included these data in **Fig. 2c** and **Extended Data Fig. 1c–d**, and revised the text accordingly (**p. 6, lines 20–32**):

*"To determine whether CG5828 functions as an active metabolite phosphatase, we purified recombinant GST-tagged protein from bacteria (Extended Data Fig. 1c) and subjected it to in vitro phosphatase assays. CG5828 exhibited robust phosphatase activity towards both the generic phospho-substrate para-nitrophenyl phosphate (PNPP) and PANK4's proposed endogenous substrate, the CoA synthesis intermediate 4'-phosphopantetheine (p-PaSH). Importantly, mutation of the predicted catalytic aspartates (D209 or D245) abolished activity (Fig. 2c). Consistent with human PANK4 and other DUF89*

*family members, CG5828 phosphatase activity requires a divalent metal cation, with activity robustly promoted by cobalt and nickel and to a lesser extent by magnesium and manganese. In contrast, other biologically relevant monovalent or divalent cations failed to support its enzymatic activity (Extended Data Fig. 1d). Based on the sequence and biochemical features, we designate CG5828 as the functional Drosophila ortholog of PANK4 (dPANK4)."*

Referring to Fig. 2k, there seems to be some confusion with the terminology, and possibly the data. The data displayed seem to be a fold change over the control. Accordingly, most grey control bars hover around the 1.0 mark. For CoA, however, it rises to 1.1, with all data points well above 1.0. If the control is normalised to 1, this should not be possible. Please check this data. Also, the authors refer to these values as fractional enrichment. Usually, when performing isotope tracing, the fractional enrichment refers to values of labelled over unlabelled forms of any given metabolite. Here I cannot see the non-labelled values. Or are these fractions that are then further normalised to the control, which would be a rather unusual thing to do? Please clarify this point.

There was an error in the normalization of these data; thank you for pointing this out. We have reprocessed the replicates, recomputed the normalization, corrected the plots, and updated the figure in the revised manuscript. In addition, we have specified the analysis workflow in the Methods section in **p. 26, lines 11-14**.

And thank you for your question regarding fractional enrichment. We followed the standard calculation protocol used previously in our laboratory [4, 16]. Briefly, isotopologue abundances for each metabolite were normalized to the total pool (labeled + unlabeled) of that metabolite and additionally normalized to the proportion of labeled VB5 within each sample to account for potential differences in tracer intake. Normalized values were then expressed as fold change relative to control. These details have been added to the **Methods** in **p. 23, lines 26-30**:

Minor points:

- The H3 signal is quite noisy. An overlay with arrows pointing at those cells counted for quantification would be of great help.

Thank you for the comments. We have included arrowheads to denote $pH3^+$ cells, except in Yki samples where the extensive signal rendered such annotations impractical.

- Does simvastatin also increase survival?

Thank you for the comments. We tested whether simvastatin feeding extends the survival of tumor-bearing flies and observed a modest lifespan rescue (Kaplan–Meier, log-rank p = 0.0529). However, because simvastatin was delivered in DMSO, we also noted shortened lifespan in control animals fed either DMSO (vehicle control) or simvastatin, consistent with vehicle-associated toxicity. Together with the reductions in tumor burden and tumor-associated phenotypes observed with simvastatin, these data indicate that MVA pathway inhibition suppresses Yki tumor growth. These results are included in **Extended Data Fig. 3m** and clarified in the text (**p. 12, lines 29-30**):

*"Consistent with our hypothesis, simvastatin treatment significantly reduced tumor cell proliferation (Fig. 4l), ameliorated the associated bloating phenotype (Fig. 4m and Extended Data Fig. 3l), and extended their overall survival (Extended Data Fig. 3m)."*

- Transcriptional levels of Myc as a readout for its activity are often difficult due to its short half-life. Using Myc signatures often yields better results. The authors might consider this.

Thank you for the suggestion. We agree that Myc transcript abundance is an inadequate proxy for activity. Accordingly, we performed transcription factor (TF) regulon analyses on our published snRNA-seq datasets, which infer TF activity from the coordinated expression of direct targets. Myc emerged as one of the most significantly activated TFs in MTs of Yki flies (**Extended Data Fig. 4b**) and in MTts > Pvr[Act] flies (**Extended Data Fig. 4n**). These analyses indicate that Myc activity is elevated under these conditions.

- In panels such as 2j, the p-value is displayed in two ways, position in the graph and colour. Personally, I do not think the same information should be displayed twice, but this is ultimately an editorial decision.

We appreciate the comment. For this revision, we did not alter the dual p-value encoding; however, we are happy to conform to a single-format display per the journal's preference at the production stage.

I hope the comments can be of help to the authors.

Thank you very much for your insightful comments, which have substantially improved the quality of our manuscript. We sincerely appreciate your time and effort.

Reviewer #4 (Remarks to the Author):

I co-reviewed this manuscript with one of the reviewers who provided the listed reports. This is part of the Nature Communications initiative to facilitate training in peer review and to provide appropriate recognition for Early Career Researchers who co-review manuscripts.

## References


1. Lee, W.-C. and C.A. Micchelli, *Development and characterization of a chemically defined food for Drosophila.* PloS one, 2013. **8**(7): p. e67308.
2. Piper, M.D., et al., *A holidic medium for Drosophila melanogaster.* Nature methods, 2014. **11**(1): p. 100-105.
3. Troen, A.M., et al., *Lifespan modification by glucose and methionine in Drosophila melanogaster fed a chemically defined diet.* Age, 2007. **29**(1): p. 29-39.
4. Liu, Y., et al., *Hepatic gluconeogenesis and PDK3 upregulation drive cancer cachexia in flies and mice.* Nature Metabolism, 2025. **7**(4): p. 823-841.
5. Liu, Z., H. Zhang, B. Lemaitre, and X. Li, *Duox activation in Drosophila Malpighian tubules stimulates intestinal epithelial renewal through a countercurrent flow.* Cell Reports, 2024. **43**(4).
6. Sorrentino, G., et al., *Metabolic control of YAP and TAZ by the mevalonate pathway.* Nature cell biology, 2014. **16**(4): p. 357-366.
7. Hoffman, B. and D. Liebermann, *Apoptotic signaling by c-MYC.* Oncogene, 2008. **27**(50): p. 6462-6472.
8. Steller, H., *Regulation of apoptosis in Drosophila.* Cell Death & Differentiation, 2008. **15**(7): p. 1132-1138.
9. Prendergast, G.C., *Mechanisms of apoptosis by c-Myc.* Oncogene, 1999. **18**(19): p. 2967-2987.
10. Harrington, E.A., M.R. Bennett, A. Fanidi, and G. Evan, *c-Myc-induced apoptosis in fibroblasts is inhibited by specific cytokines.* The EMBO journal, 1994. **13**(14): p. 3286-3295.
11. Evan, G. and T. Littlewood, *A matter of life and cell death.* Science, 1998. **281**(5381): p. 1317-1322.
12. Khaminets, A., et al., *Cohesin controls intestinal stem cell identity by maintaining association of Escargot with target promoters.* Elife, 2020. **9**: p. e48160.
13. Ren, F., et al., *Hippo signaling regulates Drosophila intestine stem cell proliferation through multiple pathways.* Proceedings of the National Academy of Sciences, 2010. **107**(49): p. 21064-21069.
14. Zhao, B., et al., *Inactivation of YAP oncoprotein by the Hippo pathway is involved in cell contact inhibition and tissue growth control.* Genes & development, 2007. **21**(21): p. 2747-2761.
15. Xu, J., et al., *Mechanistic characterization of a Drosophila model of paraneoplastic nephrotic syndrome.* Nature Communications, 2024. **15**(1).
16. Jouandin, P., et al., *Lysosomal cystine mobilization shapes the response of mTORC1 and tissue growth to fasting.* Science, 2022. **375**(6582).
